# The temporal trend of disease burden attributable to metabolic risk factors in China, 1990–2019: An analysis of the Global Burden of Disease study

**DOI:** 10.3389/fnut.2022.1035439

**Published:** 2023-01-04

**Authors:** Yingzhao Jin, Ho So, Ester Cerin, Anthony Barnett, Sumaira Mubarik, Kamal Hezam, Xiaoqi Feng, Ziyue Wang, Junjie Huang, Chenwen Zhong, Khezar Hayat, Fang Wang, Ai-Min Wu, Suowen Xu, Zhiyong Zou, Lee-Ling Lim, Jiao Cai, Yimeng Song, Lai-shan Tam, Dongze Wu

**Affiliations:** ^1^Department of Medicine and Therapeutics, The Prince of Wales Hospital, The Chinese University of Hong Kong, Hong Kong, China; ^2^Mary MacKillop Institute for Health Research, Australian Catholic University, Melbourne, VIC, Australia; ^3^School of Public Health, The University of Hong Kong, Hong Kong, Hong Kong SAR, China; ^4^Department of Epidemiology and Biostatistics, Wuhan University, Wuhan, China; ^5^Nankai University School of Medicine, Tianjin, China; ^6^Department of Microbiology, Faculty of Applied Science, Taiz University, Taiz, Yemen; ^7^Faculty of Medicine and Health, School of Population Health, University of New South Wales, Sydney, NSW, Australia; ^8^Population Wellbeing and Environment Research Lab (PowerLab), Wollonggong, NSW, Australia; ^9^The George Institute for Global Health, Newtown, NSW, Australia; ^10^Department of Family Medicine, McGill University, Montreal, QC, Canada; ^11^China Centre for Health Development Studies, Peking University, Beijing, China; ^12^Jockey Club School of Public Health and Primary Care, The Chinese University of Hong Kong, Hong Kong, China; ^13^Institute of Pharmaceutical Sciences, University of Veterinary and Animal Sciences, Lahore, Pakistan; ^14^Department of Pharmacy Administration and Clinical Pharmacy, Xi'an Jiaotong University, Xi'an, China; ^15^School of Public Health, Xuzhou Medical University, Xuzhou, China; ^16^Department of Orthopaedics, Wenzhou Medical University, Wenzhou, China; ^17^Department of Endocrinology, University of Science and Technology of China, Hefei, China; ^18^Institute of Child and Adolescent Health, Peking University, Beijing, China; ^19^Department of Medicine, Faculty of Medicine, University of Malaya, Kuala Lumpur, Malaysia; ^20^Institute for Health and Environment, Chongqing University of Science and Technology, Chongqing, China; ^21^School of the Environment, Yale University, New Haven, CT, United States; ^22^Department of Rheumatology and Immunology, Sichuan Provincial People's Hospital, University of Electronic Science and Technology of China, Chengdu, China; ^23^Chinese Academy of Sciences Sichuan Translational Medicine Research Hospital, Chengdu, China

**Keywords:** disease burden, metabolic risk factors, temporal trend, aging, China

## Abstract

**Background and aims:**

The disease burden attributable to metabolic risk factors is rapidly increasing in China, especially in older people. The objective of this study was to (i) estimate the pattern and trend of six metabolic risk factors and attributable causes in China from 1990 to 2019, (ii) ascertain its association with societal development, and (iii) compare the disease burden among the Group of 20 (G20) countries.

**Methods:**

The main outcome measures were disability-adjusted life-years (DALYs) and mortality (deaths) attributable to high fasting plasma glucose (HFPG), high systolic blood pressure (HSBP), high low-density lipoprotein (HLDL) cholesterol, high body-mass index (HBMI), kidney dysfunction (KDF), and low bone mineral density (LBMD). The average annual percent change (AAPC) between 1990 and 2019 was analyzed using Joinpoint regression.

**Results:**

For all six metabolic risk factors, the rate of DALYs and death increased with age, accelerating for individuals older than 60 and 70 for DALYs and death, respectively. The AAPC value in rate of DALYs and death were higher in male patients than in female patients across 20 age groups. A double-peak pattern was observed for AAPC in the rate of DALYs and death, peaking at age 20–49 and at age 70–95 plus. The age-standardized rate of DALYs increased for HBMI and LBMD, decreased for HFPG, HSBP, KDF, and remained stable for HLDL from 1990 to 2019. In terms of age-standardized rate of DALYs, there was an increasing trend of neoplasms and neurological disorders attributable to HFPG; diabetes and kidney diseases, neurological disorders, sense organ diseases, musculoskeletal disorders, neoplasms, cardiovascular diseases, digestive diseases to HBMI; unintentional injuries to LBMD; and musculoskeletal disorders to KDF. Among 19 countries of Group 20, in 2019, the age-standardized rate of DALYs and death were ranked fourth to sixth for HFPG, HSBP, and HLDL, but ranked 10th to 15th for LBMD, KDF, and HBMI, despite the number of DALYs and death ranked first to second for six metabolic risk factors.

**Conclusions:**

Population aging continuously accelerates the metabolic risk factor driven disease burden in China. Comprehensive and tight control of metabolic risk factors before 20 and 70 may help to mitigate the increasing disease burden and achieve healthy aging, respectively.

## Introduction

Increasing life expectancy has led to a global burden of late-life disease and research has recently been focused on ways of avoiding this trend in the general population (1). In China the period from 1950 to 2019 has seen the total fertility rate decrease from 5.91 to 1.43, yet life expectancy has increased from 49.6 to 74.7 years for men and from 52.6 to 80.8 years for women (2). The summary exposure values of metabolic risk factors increased from 14.90 to 22.14 in China from 1990 to 2019 with an annualized rate of change of 1.37% (3).

Understanding the contributions of metabolic risk factors to disease over time is vital to enabling healthy extended lifespans (4). According to a Global Burden of Disease (GBD) study from 2019, metabolic risk factors include high fasting plasma glucose (HFPG), high systolic blood pressure (HSBP), high low-density lipoprotein (HLDL) cholesterol, high body-mass index (HBMI), kidney dysfunction (KDF), and low bone mineral density (LBMD) (3). Metabolic risk factors have become the leading cause of ischemic heart disease in developing countries (5). The associations between type 2 diabetes with different cardiovascular diseases, including peripheral arterial disease, ischemic stroke, stable angina, heart failure, and non-fatal myocardial infarction have been established (6). Around 31.7% and 23.3% of patients with hypertension had blood pressure below 140/90 mm Hg and below 130/80 mm Hg, contributing to heart attack, stroke, and chronic kidney disease (7). HBMI accounted for 4.0 million deaths globally, more than two-thirds of which were due to cardiovascular disease, and nearly 40% of these occurred in people who were not obese (8). LBMD-associated increase in fracture risk affects virtually all skeletal sites, especially among older women and patients treated with glucocorticoid (9, 10). Occurring in a continuum with acute and chronic kidney disease, people with KDF are 5–10 times more likely to die prematurely, largely due to cardiovascular disease and cancer (11, 12). The principal target of lipid-modification therapies is to lower LDL cholesterol to prevent cardiovascular death, although recent research focuses on triglyceride-rich lipoproteins in addition to LDL as the causal risk factor for atherosclerosis (13).

Over the past three decades, the role of metabolic aging in extending a healthy lifespan has been increasingly acknowledged with population aging (14). Available evidence supports the idea that decreased nutrient signaling extends longevity and anabolic signaling accelerates aging (15). Previous study has investigated the mortality, morbidity, and risk factors in China (16). Therefore, our study further analyzed the disease burden of metabolic risk factors in the era of population aging. The Group of 20 (G20) may provide significant research insights that are highly relevant to the Chinese context, as the mean population age in China was lower than most countries in G20 (2).

The objectives of this study were to (1) investigate the pattern of disease burden driven by six metabolic risk factors disaggregated by age and sex, (2) characterize the temporal trend of the six metabolic risk factors, (3) ascertain the temporal trend of metabolic risk attributable cause, (4) determine the association between societal development and metabolic risk factors, and (5) compare metabolic risk factors in China with those of G20 countries.

## Methods

### Data sources and data extraction

Global Burden of Disease study (GBD) 2019 was established to quantify the health loss caused by diseases, injuries, and risk factors, including 369 diseases, injuries, and 87 risk factors [including six metabolic risk factors–HFPG, HSBP, HLDL, HBMI, KDF, and LBMD (Case definition in Supplementary material)] (3, 17). This study was produced as part of the GBD Collaborator Network and in accordance with the GBD Protocol (IHME ID. 1775-GBD2019-012021). The study collected original data from the Global Health Data Exchange (GHDx), including age-sex-year disability adjusted life years (DALYs), years lived with disability (YLDs), years of life lost (YLLs), and death of six metabolic risk factors in terms of absolute number, age-standardized rate, and crude rate per 100,000 population.

### Inclusion and exclusion criteria

According to the comparative risk assessment conceptual framework, the GBD 2019 study established a causal web of hierarchically organized risks or causes that contributed to health outcomes. A set of behavioral, environmental, occupational, and metabolic risk factors-outcome pairs were constructed based on evidence rules. The study included 23, 66, and 61 metabolic risk factor-level 2, 3, and 4 outcome pairs, and 108 risk-most detailed outcome pairs (The GBD metabolic risk factor attributable cause hierarchy in Supplementary material) (3). The study excluded GBD behavioral, environmental, and occupational risk factors attributable to cause hierarchy.

### Population attributable fraction and socio-demographic index

The population attributable fractions (PAFs) were used to quantify the contribution of risk factors to the burden of disease (Estimation of six metabolic risk factors in Supplementary material) (18). The sociodemographic index (SDI) is a composite indicator of socio-demographic development status, which is strongly correlated with health outcomes. It is the geometric mean of 0 to 1 indices of total fertility rate in those under 25 years old, mean education for those aged 15 years or older, and lag-distributed income per capita. The national SDIs for China between 1990 and 2019 ranged from 0.433 to 0.686 (17).

### Statistical analysis

The number and rate of DALYs, YLDs, YLLs, and deaths with 95% uncertainty intervals (UIs) of the six metabolic risk factors were reported according to age and sex from 1990 to 2019. Age-standardized rates of DALYs and death were plotted against SDI between 1990 and 2019 with a simple correlation. Temporal trend changes were determined using a Joinpoint regression model. Average annual percent change (AAPC) was calculated for the entire period analyzed, and annual percent change (APC) was calculated for each segmented line regression. The temporal trends were defined according to the statistical significance of the AAPC compared to zero. Any AAPC or APC with a 95% CI overlapping with zero was considered stable. All statistical analyses were performed using Joinpoint Regression Program (version 4.8.0.1, Statistical Methodology and Applications Branch, Surveillance Research Program, National Cancer Institute), with *P*-values < 0.05 considered statistically significant.

## Results

### Trend of disease burden driven by six metabolic risk factors according to age and year

**Figure 1:**
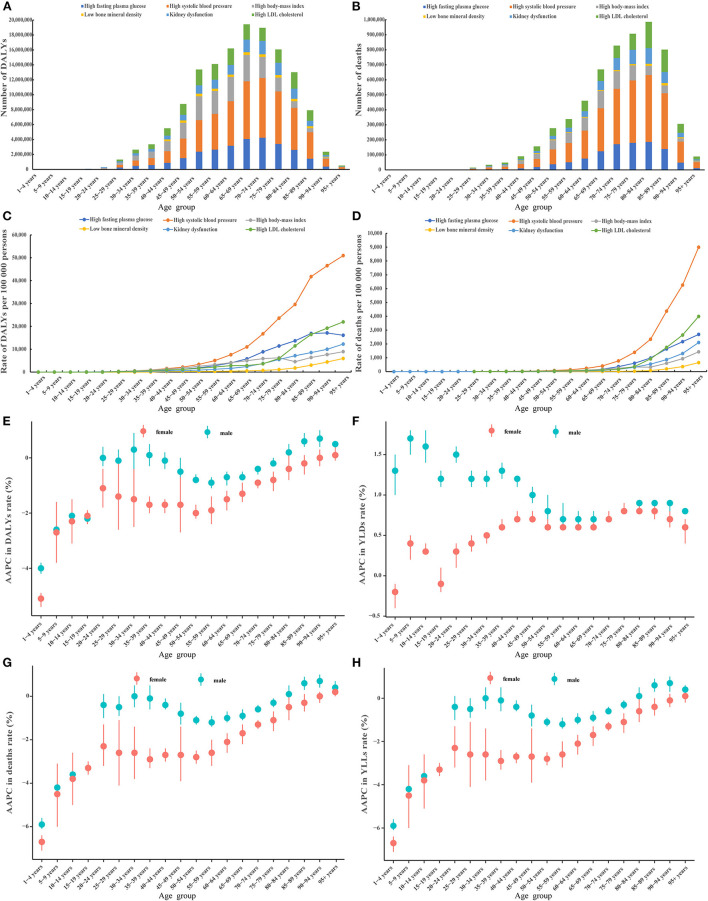
Cross-sectional and longitudinal trend of the disease burden attributable to six metabolic risk factors throughout the human lifespan. Number of DALYs attributable to six metabolic risk factors across 20 age groups in 2019 **(A)**, number of deaths attributable to six metabolic risk factors across 20 age groups in 2019 **(B)**, rate of DALYs attributable to six metabolic risk factors across 20 age groups in 2019 **(C)**, rate of deaths attributable to six metabolic risk factors across 20 age groups in 2019 **(D)**, AAPC in the rate of DALYs attributable to metabolic risk factors across 20 age groups, 1990–2019 **(E)**, AAPC in the rate of YLDs attributable to metabolic risk factors across 20 age groups, 1990–2019 **(F)**, AAPC in the rate of deaths attributable to metabolic risk factors across 20 age groups, 1990–2019 **(G)**, AAPC in the rate of YLLs attributable to metabolic risk factors across 20 age groups, 1990–2019 **(H)**. AAPC, average annual percent change; DALYs, disability-adjusted life years; YLDs, years lived with a disability; YLLs, years of life lost.

We first addressed how metabolic risk factor driven disease burden changed with increasing age. Overall, people aged 65–69 and 80–84 years had the highest number of DALYs and deaths, respectively (Figures 1A, B). The rate of DALYs and death increased with age, especially for people older than 60 and 70 for DALYs and deaths, respectively (Figures 1C, D). Notably, the disease burden attributable to KDF and LBMD increased rapidly with age (Supplementary Figures 1E, F, 2E, F). The disease burden was higher in male patients than female patients across different age groups for each (Supplementary Figures 1A–F, 2A–F) and all of the metabolic risk factors (Figures 1E–H). Besides, a double-peak pattern was observed for AAPC in the rate of DALYs, deaths, and YLLs, peaking at age 20 to 49 and at age 70 to 95 plus (Figures 1E, G, H); while a trumpet pattern was found for AAPC in the rate of YLDs (Figure 1F). A double-peak pattern was also observed for AAPC in the rate of HFPG, HLDL, HSBP (Supplementary Figures 1B–D, 2B–D).

We further determined how the disease burden changed with age. A strong increasing trend was found across the 16 age groups, especially in people older than 60 (Supplementary Figure 3). Amongst all subjects aged 15 to 94, individuals aged 60–74 contributed the greatest numbers of DALYs and the 75–94 age group contributed the greatest number of deaths (Supplementary Figures 4, 5).

We next ascertained if the gender differences in the six metabolic risk factors existed. The numbers and age-standardized rate of DALYs and deaths were higher in male than female patients for five out of six metabolic risk factors (except LBMD) (Supplementary Figure 6, Supplementary Table 1). The age-standardized rate of YLDs was higher in male than female patients for five out of six metabolic risk factors (except HFPG), while the age-standardized rate of YLLs was higher in female than male patients for all six metabolic risk factors (Supplementary Figure 7).

### Temporal trend of metabolic risk factors from 1990 to 2019

From 1990 to 2019, the number of DALYs and deaths attributable to the six metabolic risk factors showed rapid growth, with the highest rate for HBMI (DALYs: 4.0; death: 4.2) with no sex-based difference (Table 1, Supplementary Figure 8, Supplementary Table 1). The age standardized rate of DALYs increased for HBMI (1.28) and LBMD (0.18), decreased for HFPG (−0.41), HSBP (−0.89), KDF (−0.61), and remained stable for HLDL. The age standardized rate of death increased for HBMI (1.03), LBMD (0.38), HLDL (0.45), decreased for HSBP (−0.78) and remained stable for HFPG (−0.41) and KDF (−0.10) (Table 1, Supplementary Figure 8). A sex difference was identified in the trend of the age-standardized rate of DALYs for HFPG and HLDL, the age-standardized rate of death for HFPG, HSBP, KDF, and HLDL (Supplementary Table 1).

**Table 1:** Trends in the number and age-standardized rate of DALYs and death attributable to six metabolic risk factors for both sexes in China, 1990–2019.

		**1990**	**2019**	**AAPC** **(95% CI)**	**APC** **(1990–1999)**	**APC** **(2000–2009)**	**APC** **(2009–2019)**
**DALYs**	**Number (95% UI)**						
	High fasting plasma glucose	13,423,677 (10,777,527–16,387,796)	28,228,439 (22,052,698–35,375,022)	2.5 (2.1–2.9)	2.8 (2.2–3.4)	3.7 (2.9–4.6)	1.1 (0.6–1.7)
	High systolic blood pressure	29,372,404 (24,312,404–34,731,124)	54,441,615 (45,474,002–63,689,070)	2.1 (2.0–2.3)	1.6 (1.5–1.7)	2.7 (2.4–3.0)	1.8 (1.8–1.9)
	High body-mass index	7,876,348 (2,002,230–16,754,458)	24,830,041 (11,788,976–40,545,899)	4.0 (3.9–4.2)	3.9 (3.8–4.0)	4.4 (3.9–4.9)	3.6 (3.5–3.7)
	Low bone mineral density	1,339,535 (1,100,875–1,640,114)	3,320,275 (2,594,480–4,004,265)	3.4 (3.2–3.5)	3.4 (3.2–3.5)	3.4 (3.2–3.5)	3.4 (3.2–3.5)
	Kidney dysfunction	7,678,849 (6,630,862–8,750,551)	13,353,909 (11,150,932–15,771,793)	2.0 (1.8–2.2)	1.3 (1.1–1.5)	2.9 (2.4–3.3)	1.5 (1.0–1.9)
	High LDL cholesterol	8,889,099 (7,139,095–11,088,753)	19,813,962 (15,205,317–25,139,359)	2.8 (2.7–3.0)	2.0 (1.9–2.1)	3.8 (3.5–4.1)	2.2 (1.9–2.5)
	**Age standardized rate per 100,000 population** **(95% UI)**						
	High fasting plasma glucose	1,605 (1,283–1,965)	1,452 (1,139–1,832)	−0.4 (−0.8 to 0.0)	0.1 (−0.4 to 0.7)	0.8 (0.0–1.6)	−2.1 (−2.6 to −1.6)
	High systolic blood pressure	3,672 (3,073–4,301)	2,844 (2,392–3,321)	−0.9 (−1.0 to −0.8)	−1.3 (−1.4 to −1.3)	−0.3 (−0.6 to 0)	−1.4 (−1.5 to −1.3)
	High body-mass index	852 (214–1,834)	1,231 (578–2,023)	1.3 (1.1–1.4)	1.1 (1.0–1.2)	1.6 (1.2–1.9)	1.0 (0.8–1.2)
	Low bone mineral density	168 (138–204)	177 (138–214)	0.2 (0.0–0.3)	−0.4 (−0.8 to 0.0)	0.7 (0.6–0.8)	0.2 (0.0–0.4)
	Kidney dysfunction	863 (747–995)	709 (593–832)	−0.6 (−0.9 to −0.4)	−1.2 (−1.5 to −0.9)	0.5 (0.2–0.8)	−1.5 (−2.0 to−0.9)
	High LDL cholesterol	1,046 (826–1,346)	1,052 (800–1,345)	0.0 (−0.1 to 0.1)	−0.7 (−0.8 to −0.7)	1.2 (0.8–1.5)	−0.8 (−0.8 to −0.7)
**Death**	**Number** **(95% UI)**						
	High fasting plasma glucose	450,809 (343,516–575,374)	1,067,554 (793,048–1,442,201)	2.9 (2.3–3.4)	3.3 (2.9–3.7)	4.2 (2.9–5.6)	1.1 (0.4–1.8)
	High systolic blood pressure	1,222,195 (1,017,782–1,445,891)	2,599,879 (2,138,699–3,082,235)	2.6 (2.4–2.8)	1.7 (1.6–1.8)	3.4 (3.0–3.9)	2.2 (2.1–2.3)
	High body-mass index	234,998 (58,375–513,815)	764,698 (333,163–1,310,557)	4.2 (3.9–4.4)	3.7 (3.6–3.9)	4.4 (3.9–4.9)	4.0 (3.7–4.4)
	Low bone mineral density	32,002 (26,081–42,555)	89,857 (58,927–110,423)	4.3 (3.9–4.7)	4.3 (3.9–4.7)	4.3 (3.9–4.7)	4.3 (3.9–4.7)
	Kidney dysfunction	248,344 (210,134–288,765)	573,537 (467,902–690,324)	3.0 (2.7–3.3)	1.8 (1.7–2.0)	4.4 (3.7–5.1)	2.2 (1.8–2.6)
	High LDL cholesterol	317,060 (244,158–413,836)	915,983 (647,993–1,239,382)	3.7 (3.6–3.9)	2.4 (2.3–2.5)	5.3 (4.9–5.7)	3.0 (2.8–3.1)
	**Age standardized rate per 100,000 population** **(95% UI)**						
	High fasting plasma glucose	67 (51–89)	62 (45–85)	−0.4 (−0.9–0.1)	0.4 (0.1–0.7)	0.9 (0.0–1.9)	−2.5 (−3.8 to −1.3)
	High systolic blood pressure	191 (160–226)	153 (126–182)	−0.8 (−0.9 to −0.7)	−1.3 (−1.4 to −1.2)	0.0 (−0.3–0.4)	−1.5 (−1.6 to −1.4)
	High body-mass index	30 (7–67)	41 (18–71)	1.0 (0.8–1.3)	0.8 (0.6–1.0)	1.4 (0.7–2.0)	0.6 (0.5–0.8)
	Low bone mineral density	5 (4–7)	6 (4–7)	0.4 (0.2–0.6)	−0.7 (−0.9 to −0.5)	3.1 (2.7–3.4)	−1.3 (−1.3 to −1.2)
	Kidney dysfunction	36 (30–42)	34 (27–41)	−0.1 (−0.4 to 0.2)	−0.9 (−1.1 to −0.6)	1.4 (0.5–2.2)	−1.3 (−1.6 to −1.1)
	High LDL cholesterol	49 (35–68)	56 (38–77)	0.4 (0.3–0.6)	−0.3 (−0.4 to −0.2)	2.1 (1.7–2.4)	−0.9 (−1 to −0.8)

AAPC, average annual percent changes; APC, annual percent change; DALYs, disability-adjusted life years; UI, uncertainty interval; CI, confidence interval.

For the 22 level 2 causes, the number of DALYs and death increased from 1990 to 2019, with the highest speed in HBMI attributable neurological disorders (DALYs: 6.7; death: 6.8) (Table 2). In terms of age-standardized rate of DALYs, there was an increasing trend of neoplasms and neurological disorders attributable to HFPG, diabetes and kidney diseases, neurological disorders, sense organ diseases, musculoskeletal disorders, neoplasms, cardiovascular diseases, digestive diseases attributable to HBMI, unintentional injuries attributable to LBMD, and musculoskeletal disorders attributable to KDF (Table 2). In terms of age-standardized rate of death, there was an increasing trend of neoplasms attributable to HFPG, cardiovascular diseases attributable to HSBP, neurological disorders, diabetes and kidney diseases, neoplasms, cardiovascular diseases attributable to HBMI, unintentional injuries attributable to LBMD (Table 2). A similar trend was also observed in level 3, level 4, and detailed causes (Supplementary Tables 2–4).

**Table 2:** Trends in the number and age-standardized rate of DALYs and death for level 2 causes attributable to six metabolic risk factors in China, 1990–2019.

	**Number (95% UI)**						**Age standardized rate per 100,000 population (95% UI)**					
	**1990**	**2019**	**AAPC** **(95% CI)**	**APC** **(1990–1999)**	**APC** **(2000–2009)**	**APC** **(2009–2019)**	**1990**	**2019**	**AAPC** **(95% CI)**	**APC** **(1990–1999)**	**APC** **(2000–2009)**	**APC** **(2009–2019)**
**DALYs**												
**High fasting plasma glucose**												
Neoplasms	603,435 (152,503–1,309,632)	1,955,214 (496,063–4,292,456)	4.1 (3.9–4.3)	4.1 (3.8–4.3)	5.7 (5.4–6.0)	2.6 (2.1–3.1)	70 (18–151)	95 (24–210)	1.0 (0.8–1.3)	1.4 (1.1–1.7)	2.8 (2.4–3.1)	−0.9 (−1.5 to −0.3)
Cardiovascular diseases	6,657,471 (4,752,045–9,121,111)	13,585,849 (9,679,707–18,867,220)	2.4 (1.8–3.0)	3.1 (2.2–4.0)	3.6 (2.4–4.7)	0.5 (−0.3 to 1.4)	856 (611–1,173)	715 (502–1,022)	−0.7 (−1.3 to −0.2)	0.3 (−0.6 to 1.2)	0.7 (−0.5 to 2.0)	−3.1 (−3.9 to −2.3)
Neurological disorders	124,438 (18,782–445,419)	442,695 (73,488–1,425,147)	4.4 (4.2–4.6)	4.8 (4.4–5.2)	5.6 (5.1–6.0)	2.9 (2.7–3.2)	24 (4–85)	27 (4–89)	0.4 (0.2–0.6)	1.2 (0.8–1.6)	1.7 (1.3–2.1)	−1.6 (−1.8 to −1.3)
Sense organ diseases	31,679 (7,189–76,325)	84,914 (18,868–200,923)	3.5 (3.3–3.7)	5.9 (5.5–6.3)	3.5 (3.2–3.8)	1.5 (1.1–1.9)	5 (1–12)	5 (1–11)	−0.2 (−0.4 to 0.1)	2.2 (1.8–2.6)	−0.3 (−0.6 to 0.0)	−2.4 (−2.7 to −2.0)
Respiratory infections and tuberculosis	389,268 (234,926–568,709)	125,538 (73,808–183,637)	−3.9 (−4.3 to −3.5)	−3.9 (−4.8 to −2.9)	−4.0 (−4.7 to −3.3)	−3.8 (−4.2 to −3.5)	42 (25–62)	6 (4–9)	−6.4 (−6.8 to −6.1)	−6.4 (−7.3 to −5.5)	−7.1 (−7.8 to −6.5)	−6.2 (−6.5 to −5.9)
Diabetes and kidney diseases	5,617,387 (4,687,144–6,682,997)	12,034,230 (9,908,708–14,504,239)	2.6 (2.5–2.8)	2.6 (2.5–2.7)	3.4 (3.0–3.8)	1.9 (1.8–2.0)	608 (510–718)	604 (499–726)	0.0 (−0.1 to 0.1)	0.1 (0.0–0.1)	0.7 (0.4–1.1)	−0.8 (−0.9 to −0.8)
**High systolic blood pressure**												
Cardiovascular diseases	27,793,037 (22,943,586–33,182,723)	51,069,180 (42,484,465–59,828,159)	2.1 (2.0–2.2)	1.5 (1.5–1.6)	2.6 (2.3–2.9)	1.8 (1.7–1.9)	3,491 (2,894–4,115)	2,670 (2,232–3,124)	−0.9 (−1.1 to −0.8)	−1.4 (−1.5 to −1.3)	−0.4 (−0.7 to 0.0)	−1.5 (−1.5 to −1.4)
Diabetes and kidney diseases	1,579,368 (1,317,964–1,870,237)	3,372,435 (2,783,861–4,004,996)	2.7 (2.5–3.0)	2.2 (1.9–2.5)	3.7 (3.4–3.9)	2.1 (1.5–2.7)	182 (153–211)	174 (144–206)	−0.1 (−0.3 to 0.2)	−0.7 (−0.9 to −0.4)	1.0 (0.7–1.4)	−0.9 (−1.5 to −0.2)
**High body-mass index**												
Neoplasms	885,448 (194,534–2,154,883)	2,669,799 (1,094,208–4,839,179)	3.9 (3.7–4.1)	5.0 (4.9–5.1)	2.6 (2.4–2.9)	3.9 (3.3–4.5)	94 (21–227)	128 (53–233)	1.1 (0.9–1.2)	2.3 (2.2–2.3)	−0.2 (−0.4 to 0.0)	1.1 (0.6–1.6)
Cardiovascular diseases	5,327,955 (1,329,819–11,290,734)	15,004,139 (6,947,157–24,943,213)	3.6 (3.5–3.8)	3.5 (3.3–3.6)	3.9 (3.6–4.2)	3.3 (3.1–3.4)	577 (142–1,251)	747 (344–1,254)	0.9 (0.7–1.1)	0.6 (0.4–0.9)	1.1 (0.6–1.6)	0.6 (0.5–0.7)
Chronic respiratory diseases	98,457 (25,467–228,184)	175,325 (75,052–320,742)	2.1 (1.9–2.2)	2.1 (1.9–2.3)	−0.3 (−0.5 to 0.0)	4.4 (3.9–4.9)	11 (3–26)	10 (5–19)	−0.1 (−0.3 to 0.2)	−0.5 (−0.7 to −0.3)	−2.4 (−2.9 to −1.9)	2.8 (2.3–3.2)
Digestive diseases	139,950 (32,648–342,137)	384,258 (158,489–746,027)	3.5 (3.4–3.6)	3.0 (2.9–3.1)	3.9 (3.7–4.0)	3.4 (3.3–3.5)	15 (4–37)	19 (8–37)	0.8 (0.7–0.9)	0.1 (0.0–0.3)	1.0 (0.8–1.1)	1.1 (0.9–1.3)
Neurological disorders	72,969 (8,730–252,707)	477,209 (101,059–1,388,766)	6.7 (6.6–6.8)	6.1 (6.0–6.2)	7.2 (7.0–7.4)	6.7 (6.5–6.8)	13 (2–46)	28 (6–82)	2.7 (2.6–2.8)	2.4 (2.3–2.5)	3.1 (3.0–3.3)	2.4 (2.3–2.5)
Musculoskeletal disorders	273,363 (58,598–684,499)	1,133,691 (431,660–2,264,916)	5.0 (4.9–5.1)	3.4 (3.1–3.6)	6.6 (6.5–6.6)	5.0 (4.9–5.1)	28 (6–70)	55 (21–110)	2.3 (2.2–2.4)	0.8 (0.4–1.2)	3.5 (3.5–3.6)	2.4 (2.3–2.5)
Sense organ diseases	9,122 (1,861–23,630)	47,688 (15,902–101,631)	5.9 (5.7–6.2)	7.2 (6.6–7.8)	4.7 (4.6–4.8)	5.2 (4.7–5.8)	1 (0–3)	3 (1–5)	2.4 (2.2–2.5)	3.8 (3.4–4.2)	1.3 (1.2–1.3)	1.5 (1.1–1.8)
Diabetes and kidney diseases	1,069,083 (291,743–2,209,261)	4,937,932 (2,514,211–7,732,009)	5.4 (5.3–5.5)	5.1 (5.0–5.3)	6.9 (6.6–7.2)	4.2 (4.1–4.3)	113 (30–236)	240 (122–379)	2.7 (2.5–2.8)	2.3 (2.2–2.5)	4.0 (3.6–4.4)	1.6 (1.5–1.7)
**Low bone mineral density**												
Transport injuries	617,844 (498,088–816,274)	1,274,510 (1,013,062–1,507,922)	2.5 (2.3–2.7)	2.8 (2.4–3.2)	4.8 (4.5–5.1)	−0.4 (−0.8 to 0.0)	67 (54–88)	62 (50–74)	−0.3 (−0.4 to −0.1)	−0.2 (−0.5 to 0.0)	1.4 (1.3–1.6)	−2.3 (−2.5 to −2.2)
Unintentional injuries	699,578 (569,079–854,117)	2,015,682 (1,477,479–2,535,537)	3.7 (3.6–3.8)	2.5 (2.3–2.6)	3.4 (3.3–3.5)	5.4 (5.3–5.5)	99 (81–120)	113 (82–143)	0.5 (0.3–0.6)	−0.6 (−0.7 to −0.5)	0.2 (−0.2 to 0.5)	1.9 (1.6–2.1)
Self-harm and interpersonal violence	22,113 (17,657–27,191)	30,084 (22,865–38,560)	1.1 (0.9–1.2)	1.0 (1.0–1.1)	1.3 (0.9–1.8)	0.9 (0.6–1.2)	3 (2–3)	1 (1–2)	−1.9 (−2.0 to −1.8)	−2.0 (−2.1 to −1.9)	−1.6 (−1.8 to −1.4)	−1.9 (−2.2 to −1.7)
**Kidney dysfunction**												
Cardiovascular diseases	3,669,169 (2,953,636–4,473,079)	7,475,542 (5,796,532–9,391,024)	2.5 (2.2–2.9)	1.5 (1.2–1.8)	4.1 (3.2–5.0)	1.7 (1.2–2.1)	451 (363–551)	395 (304–495)	−0.4 (−0.7 to −0.2)	−1.3 (−1.6 to −1)	1.2 (0.9–1.5)	−1.6 (−2.2 to −0.9)
Musculoskeletal disorders	12,654 (7,655–18,640)	46,523 (28,108–68,591)	4.6 (4.5–4.8)	1.6 (1.4–1.7)	6.8 (6.7–6.9)	5.5 (5.1–5.9)	2 (1–2)	2 (1–4)	1.3 (1.1–1.5)	−1.4 (−1.6 to −1.2)	3.6 (3.4–3.7)	1.9 (1.4–2.3)
Diabetes and kidney diseases	3,997,026 (3,531,282–4,458,760)	5,831,843 (4,992,206–6,645,333)	1.4 (1.0–1.7)	1.1 (0.8–1.4)	1.8 (1.5–2.0)	1.1 (0.1–2.2)	410 (364–457)	312 (268–354)	−0.9 (−1.2 to −0.5)	−1.1 (−1.3 to −0.8)	−0.4 (−0.6 to −0.1)	−1.3 (−2.4 to −0.3)
**High LDL cholesterol**												
Cardiovascular diseases	8,889,099 (7,139,095–11,088,753)	19,813,962 (15,205,317–25,139,359)	2.8 (2.7–3.0)	2.0 (1.9–2.1)	3.8 (3.5–4.1)	2.2 (1.9–2.5)	1,046 (826–1,346)	1,052 (800–1,345)	0.0 (−0.1 to 0.1)	−0.7 (−0.8 to −0.7)	1.2 (0.8–1.5)	−0.8 (−0.8 to −0.7)
**Death**												
**High fasting plasma glucose**												
Neoplasms	24,887 (6,343–53,939)	90,655 (23,078–197,161)	4.5 (4.3–4.8)	4.8 (4.4–5.1)	6.3 (5.9–6.7)	2.7 (2.0–3.3)	3 (1–7)	5 (1–10)	1.2 (1.0–1.5)	1.9 (1.6–2.3)	2.9 (2.5–3.3)	−1.0 (−1.7 to −0.3)
Cardiovascular diseases	298,051 (208,303–414,537)	700,341 (481,073–1,026,949)	2.9 (2.2–3.5)	3.8 (2.7–4.8)	4.6 (3.0–6.2)	0.3 (−0.4 to 1.1)	47 (33–69)	41 (28–62)	−0.6 (−1.3 to 0.1)	0.6 (−0.5 to 1.7)	0.8 (−1.0 to 2.5)	−3.5 (−4.2 to −2.8)
Neurological disorders	6,521 (629–26,296)	23,988 (2,536–94,088)	4.6 (4.3–4.8)	4.6 (4.5–4.7)	6.2 (5.7–6.7)	3.0 (2.5–3.5)	2 (0–7)	2 (0–7)	0.1 (−0.1 to 0.3)	0.5 (0.4–0.7)	1.7 (1.4–2.1)	−2.0 (−2.2 to −1.7)
Respiratory infections and tuberculosis	12,665 (7,319–19,035)	3,646 (2,056–5,638)	−4.3 (−4.7 to −3.8)	−3.9 (−5.0 to −2.8)	−5.3 (−6.1 to −4.5)	−4.0 (−4.4 to −3.7)	2 (1–2)	0 (0–0)	−7.0 (−7.4 to −6.6)	−6.2 (−7.1 to −5.3)	−8.3 (−9.0 to −7.5)	−7.0 (−7.3 to −6.7)
Diabetes and kidney diseases	108,685 (95,113–124,011)	248,925 (211,404–287,553)	2.9 (2.7–3.1)	3.0 (2.7–3.2)	2.8 (2.3–3.2)	2.8 (2.6–2.9)	14 (12–16)	14 (12–16)	−0.1 (−0.3 to 0.1)	0.2 (0.0–0.4)	0.0 (−0.7 to 0.6)	−0.6 (−0.7 to −0.5)
**High systolic blood pressure**												
Cardiovascular diseases	1,172,880 (972,201–1,384,099)	2,471,920 (2,026,048–2,942,576)	2.6 (2.4–2.8)	1.7 (1.6–1.8)	3.4 (2.9–3.9)	2.2 (2.1–2.3)	184 (153–218)	146 (120–174)	−0.8 (−1.0 to −0.7)	−1.3 (−1.4 to −1.2)	0.0 (−0.4 to 0.4)	−1.5 (−1.6 to −1.4)
Diabetes and kidney diseases	49,315 (41,426–58,188)	127,960 (105,241–151,102)	3.3 (3.2–3.5)	2.4 (2.1–2.7)	4.3 (4.0–4.6)	2.9 (2.8–3.0)	7 (6–8)	7 (6–9)	0.1 (−0.1 to 0.2)	−0.5 (−0.6 to −0.4)	1.1 (0.7–1.5)	−0.9 (−1.0 to −0.7)
**High body-mass index**												
Neoplasms	30,067 (6,567–73,656)	100,442 (41,168–185,335)	4.3 (4.2–4.4)	5.2 (5.0–5.3)	3.2 (3.1–3.2)	4.4 (4.2–4.7)	4 (1–9)	5 (2–9)	1.2 (0.9–1.5)	2.3 (1.9–2.7)	0.2 (0.0–0.3)	1.1 (0.3–1.9)
Cardiovascular diseases	178,080 (43,400–389,585)	549,540 (242,018–949,280)	3.9 (3.7–4.2)	3.4 (3.2–3.5)	4.4 (3.8–4.9)	3.7 (3.6–3.8)	22 (5–50)	29 (13–52)	0.9 (0.7–1.1)	0.4 (0.2–0.5)	1.3 (0.9–1.8)	0.6 (0.5–0.7)
Chronic respiratory diseases	2,514 (549–6,524)	3,270 (1,235–6,227)	0.9 (0.5–1.3)	1.6 (1.5–1.7)	−0.8 (−1.7 to 0.2)	1.8 (1.6–2.1)	0 (0–1)	0 (0–0)	−2.4 (−2.7 to −2.2)	−1.4 (−1.6 to −1.3)	−4.1 (−4.9 to −3.2)	−1.9 (−2.1 to −1.8)
Digestive diseases	1,846 (428–4,213)	3,053 (1,235–5,627)	1.7 (1.3–2.1)	1.6 (0.6–2.5)	0.2 (−0.2 to 0.5)	3.3 (2.8–3.8)	0 (0–1)	0 (0–0)	−1.4 (−1.8 to −1.1)	−1.1 (−1.9 to −0.2)	−2.7 (−3 to −2.5)	−0.6 (−1.0 to −0.2)
Neurological disorders	3,531 (290–13,782)	23,787 (3,264–78,659)	6.8 (6.7–6.9)	5.8 (5.7–5.9)	7.6 (7.3–7.8)	6.8 (6.6–7.0)	1 (0–3)	2 (0–6)	2.3 (2.2–2.5)	1.7 (1.4–2.0)	3.1 (2.9–3.3)	2.1 (1.9–2.2)
Diabetes and kidney diseases	18,961 (5,077–39,582)	84,607 (39,544–139,620)	5.3 (5.0–5.5)	5.1 (4.9–5.4)	5.6 (4.9–6.3)	4.8 (4.7–5.0)	2 (1–5)	4 (2–7)	2.2 (1.9–2.4)	2.3 (2.0–2.5)	2.6 (1.8–3.3)	1.3 (1.1–1.5)
**Low bone mineral density**												
Transport injuries	15,250 (12,207–21,550)	29,232 (23,067–35,070)	2.3 (2.1–2.4)	2.7 (2.4–3.1)	5.0 (4.8–5.2)	−1.3 (−1.6 to −1.0)	2 (1–2)	1 (1–2)	−0.6 (−0.7 to −0.5)	−0.1 (−0.3 to 0.2)	1.6 (1.5–1.8)	−3.9 (−4.1 to −3.7)
Unintentional injuries	16,302 (13,347–21,271)	60,259 (32,787–76,443)	4.6 (4.2–5.0)	2.1 (1.7–2.5)	7.8 (6.9–8.7)	4.0 (3.8–4.2)	3 (3–4)	4 (2–5)	0.8 (0.5–1.0)	−1.1 (−1.3 to −0.9)	3.9 (3.4–4.3)	−0.3 (−0.6 to 0.1)
Self-harm and interpersonal violence	449 (367–527)	366 (296–447)	−0.7 (−0.9 to −0.5)	0.1 (0.0–0.2)	−0.3 (−0.7 to 0.1)	−1.8 (−2.1 to −1.5)	0 (0–0)	0 (0–0)	−3.7 (−3.9 to −3.6)	−2.9 (−3.0 to −2.8)	−3.4 (−3.8 to −3.0)	−4.7 (−4.9 to −4.4)
**Kidney dysfunction**												
Cardiovascular diseases	149,737 (119,076–184,749)	376,811 (285,378–480,070)	3.3 (2.9–3.6)	1.9 (1.5–2.3)	5.2 (4.8–5.6)	2.3 (1.5–3.1)	23 (18–29)	23 (17–29)	0.0 (−0.3 to 0.3)	−0.9 (−1.1 to −0.7)	2.0 (1.3–2.8)	−1.7 (−2.2 to −1.3)
Diabetes and kidney diseases	98,607 (86,800–111,078)	196,726 (168,241–224,684)	2.4 (2.3–2.5)	1.7 (1.5–1.8)	2.9 (2.6–3.2)	2.3 (2.2–2.4)	13 (12–15)	11 (10–13)	−0.5 (−0.6 to −0.3)	−0.8 (−0.9 to −0.7)	0.2 (−0.3 to 0.6)	−1.2 (−1.3 to −1.0)
**High LDL cholesterol**												
Cardiovascular diseases	317,060 (244,158–413,836)	915,983 (647,993–1,239,382)	3.7 (3.6–3.9)	2.4 (2.3–2.5)	5.3 (4.9–5.7)	3.0 (2.8–3.1)	49 (35–68)	56 (38–77)	0.4 (0.3–0.6)	−0.3 (−0.4 to −0.2)	2.1 (1.7–2.4)	−0.9 (−1.0 to −0.8)

AAPC, average annual percent changes; APC, annual percent change; DALYs, disability-adjusted life years; UI, uncertainty interval; CI, confidence interval.

### Temporal trend of the effect of metabolic risk factors on cause from 1990 to 2019

In 2019, in terms of age-standardized rate of DALYs, HBMI contributed to 29.4, 15.1, 3.7% of diabetes and kidney diseases, cardiovascular diseases, neoplasms, HFPG to 73.8, 14.5, 2.8, 2.5% of diabetes and kidney diseases, cardiovascular diseases, neoplasms, neurological disorders. HLDL contributed to 21.3% of cardiovascular diseases, HSBP to 54.1%, 21.4% of cardiovascular diseases, diabetes and kidney diseases, KDF to 38.3%, 8.0% of diabetes and kidney diseases, cardiovascular diseases, and LBMD to 10.3%, 7.2% of unintentional injuries, transport injuries (Supplementary Table 5). Similar results were found for the contribution of the six metabolic risk factors to disease burden in terms of age-standardized rate of death (Supplementary Table 5).

Analysis of the PAF of age standardized DALYs rate indicated a significant increasing contribution of HFPG to neoplasms (2.1), respiratory infections and tuberculosis (0.7), cardiovascular diseases (0.7), neurological disorders (0.5), diabetes and kidney diseases (0.4), increasing contribution of HSBP to cardiovascular diseases (0.5), diabetes and kidney diseases (0.3), increasing contribution of HBMI to digestive diseases (4.0), chronic respiratory diseases (3.8), diabetes and kidney diseases (3.0), neurological disorders (2.8), musculoskeletal disorders (2.5), sense organ diseases (2.5), cardiovascular diseases (2.3), neoplasms (2.2), increasing contribution of LBMD to unintentional injuries (3.4), self-harm and interpersonal violence (2.0), transport injuries (1.0), increasing contribution of HLDL to cardiovascular diseases (1.4), increasing contribution of KDF to cardiovascular diseases (1.0) and musculoskeletal disorders (1.5), but a significant decreasing contribution to diabetes and kidney diseases (−0.5) (Table 3, Supplementary Figure 9). Analysis of PAF of age standardized death rate also showed a similar trend (Table 3, Supplementary Figure 10).

**Table 3:** Trends in the population attributable fraction on the age-standardized rate of DALYs and death of level 2 causes attributable to six metabolic risk factors, 1990–2019.

	**Age standardized attributable burden (per 100,000)**					
	**1990**	**2019**	**AAPC** **(95% CI)**	**APC** **(1990–1999)**	**APC** **(2000–2009)**	**APC** **(2010–2019)**
**DALYs**						
**High fasting plasma glucose**						
Cardiovascular diseases	11.5 (8.4–15.6)	14.5 (10.6–20.2)	0.7 (0.1–1.2)	1.9 (1.1–2.8)	1.7 (0.8–2.7)	−1.3 (−2.4 to −0.2)
Diabetes and kidney diseases	66.2 (62.9–69.9)	73.8 (70.4–77.5)	0.4 (0.3–0.4)	0.6 (0.5–0.7)	0.4 (0.3–0.5)	0.2 (0.0–0.3)
Neoplasms	1.5 (0.4–3.2)	2.8 (0.7–5.9)	2.1 (1.8–2.4)	2.0 (1.4–2.6)	4.4 (3.8–4.9)	0.1 (−0.2 to 0.5)
Neurological disorders	2.2 (0.4–6.1)	2.5 (0.4–7.0)	0.5 (0.3–0.7)	1.1 (0.8–1.4)	1.9 (1.6–2.1)	−1.4 (−1.6 to −1.1)
Respiratory infections and tuberculosis	1.0 (0.6–1.4)	1.2 (0.7–1.7)	0.7 (0.3–1.0)	−1.4 (−1.7 to −1.1)	3.1 (2.4–3.7)	−0.3 (−1.0 to 0.5)
Sense organ diseases	0.6 (0.1–1.3)	0.5 (0.1–1.2)	0.0 (−0.3 to 0.2)	2.0 (1.5–2.4)	0.1 (−0.2 to 0.4)	−2.0 (−2.4 to −1.6)
**High systolic blood pressure**						
Cardiovascular diseases	47.1 (40.8–53.1)	54.1 (47.6–60.5)	0.5 (0.5–0.5)	0.3 (0.3–0.4)	0.8 (0.8–0.9)	0.3 (0.3–0.3)
Diabetes and kidney diseases	19.8 (16.9–22.8)	21.4 (17.8–24.9)	0.3 (0.1–0.5)	−0.2 (−0.3 to 0.0)	0.8 (0.7–1.0)	0.0 (−0.6 to 0.6)
**High body-mass index**						
Cardiovascular diseases	7.8 (1.9–16.7)	15.1 (7.3–24.6)	2.3 (2.2–2.4)	2.3 (2.1–2.5)	2.2 (2.1–2.4)	2.5 (2.4–2.5)
Chronic respiratory diseases	0.3 (0.1–0.7)	0.8 (0.4–1.4)	3.8 (3.5–4.1)	2.4 (2.1–2.8)	2.6 (2.3–2.8)	6.5 (5.7–7.2)
Diabetes and kidney diseases	12.3 (3.4–25)	29.4 (15.8–43.5)	3.0 (2.9–3.2)	2.8 (2.7–3.0)	3.7 (3.6–3.8)	2.6 (2.3–3.0)
Digestive diseases	1.1 (0.3–2.7)	3.6 (1.5–6.5)	4.0 (3.9–4.1)	3.6 (3.3–3.8)	4.5 (4.3–4.6)	3.9 (3.8–4.0)
Musculoskeletal disorders	1.7 (0.4–3.9)	3.5 (1.4–6.3)	2.5 (2.5–2.6)	1.8 (1.7–2.0)	3.3 (3.3–3.3)	2.4 (2.3–2.5)
Neoplasms	2.0 (0.5–4.8)	3.7 (1.6–6.7)	2.2 (2.1–2.3)	2.8 (2.8–2.9)	1.5 (1.3–1.7)	2.5 (2.3–2.6)
Neurological disorders	1.2 (0.2–3.8)	2.6 (0.6–6.8)	2.8 (2.7–2.9)	2.3 (2.2–2.4)	3.3 (3.0–3.6)	2.7 (2.5–2.9)
Sense organ diseases	0.1 (0.0–0.4)	0.3 (0.1–0.6)	2.5 (2.3–2.7)	3.5 (3.0–4.0)	1.8 (1.7–1.9)	1.7 (1.3–2.0)
**Low bone mineral density**						
Self-harm and interpersonal violence	0.2 (0.2–0.3)	0.4 (0.3–0.5)	2.0 (1.7–2.3)	0.9 (0.5–1.3)	3.6 (3.1–4.0)	1.0 (0.6–1.4)
Transport injuries	5.4 (4.6–5.9)	7.2 (6.1–7.9)	1.0 (0.8–1.2)	−0.2 (−0.4 to 0.1)	2.2 (1.7–2.7)	1.1 (0.9–1.2)
Unintentional injuries	4.2 (3.5–4.9)	10.3 (8.6–11.7)	3.4 (3.1–3.7)	2.6 (2.3–2.9)	2.8 (2.6–3.1)	4.6 (4.0–5.3)
**Kidney dysfunction**						
Cardiovascular diseases	6.1 (5.0–7.2)	8.0 (6.4–9.7)	1.0 (0.9–1.1)	0.3 (0.2–0.5)	2.5 (2.4–2.7)	0.1 (−0.1 to 0.3)
Diabetes and kidney diseases	44.8 (40.8–48.6)	38.3 (33.2–42.9)	−0.5 (−0.7 to −0.3)	−0.6 (−0.8 to −0.4)	−0.7 (−0.9 to −0.5)	−0.3 (−0.7 to 0.2)
Musculoskeletal disorders	0.1 (0.1–0.1)	0.2 (0.1–0.2)	1.5 (1.4–1.6)	−0.3 (−0.4 to −0.3)	3.2 (3.1–3.3)	1.7 (1.3–2.1)
**High LDL cholesterol**						
Cardiovascular diseases	14.1 (11.4–17.6)	21.3 (16.7–26.9)	1.4 (1.4–1.5)	0.9 (0.8–1.0)	2.3 (2.3–2.4)	1.0 (0.9–1.1)
**Death**						
**High fasting plasma glucose**						
Cardiovascular diseases	12.3 (8.6–17.4)	14.9 (10.3–21.9)	0.5 (0.0–1.1)	2.0 (1.2–2.9)	1.6 (0.5–2.6)	−1.6 (−2.7 to −0.5)
Diabetes and kidney diseases	60.4 (57.2–63.3)	64.8 (61.7–67.7)	0.3 (0.2–0.3)	0.6 (0.5–0.7)	0.0 (0.0–0.1)	0.2 (0.1–0.3)
Neoplasms	1.9 (0.5–4.0)	3.3 (0.9–7.0)	1.9 (1.7–2.2)	2.3 (1.7–2.9)	3.7 (3.2–4.3)	−0.1 (−0.4 to 0.3)
Neurological disorders	4.8 (0.9–12.0)	5.4 (1.1–13.2)	0.4 (0.1–0.6)	1.0 (0.6–1.5)	1.8 (1.3–2.2)	−1.6 (−1.9 to −1.2)
Respiratory infections and tuberculosis	1.8 (1.0–2.8)	1.2 (0.7–1.8)	−1.5 (−1.9 to −1.2)	−2.0 (−2.7 to −1.2)	−0.6 (−1.2 to −0.1)	−2.4 (−3.0 to −1.7)
**High systolic blood pressure**						
Cardiovascular diseases	48.2 (41.5–55.3)	52.7 (45.1–60.7)	0.3 (0.3–0.3)	0.0 (−0.1 to 0.0)	0.6 (0.6–0.7)	0.3 (0.3–0.4)
Diabetes and kidney diseases	30.9 (27.5–34.2)	34.8 (31.1–37.8)	0.4 (0.3–0.5)	−0.1 (−0.2 to 0.0)	1.1 (1.0–1.2)	0.0 (−0.2 to 0.2)
**High body-mass index**						
Cardiovascular diseases	5.9 (1.4–13.3)	10.5 (4.7–18.2)	2.0 (2.0–2.1)	1.7 (1.5–1.8)	1.9 (1.8–2.0)	2.5 (2.5–2.6)
Chronic respiratory diseases	0.2 (0.0–0.4)	0.3 (0.1–0.5)	1.7 (1.6–1.9)	1.3 (1–1.5)	0.9 (0.7–1.1)	2.7 (2.5–2.9)
Diabetes and kidney diseases	9.9 (2.6–21.1)	20.5 (9.9–33.4)	2.5 (2.4–2.6)	2.6 (2.5–2.7)	2.7 (2.5–2.9)	2.3 (2.2–2.4)
Digestive diseases	0.7 (0.2–1.7)	1.3 (0.5–2.3)	1.9 (1.8–2.1)	2.3 (1.9–2.7)	0.7 (0.6–0.8)	3.0 (2.8–3.1)
Neoplasms	2.0 (0.5–4.9)	3.5 (1.5–6.4)	2.0 (1.9–2.0)	2.7 (2.6–2.7)	1.1 (1.0–1.3)	2.3 (2.1–2.4)
Neurological disorders	2.5 (0.4–7.4)	5.3 (1.4–12.5)	2.6 (2.5–2.7)	2.1 (1.9–2.3)	3.0 (2.9–3.2)	2.7 (2.6–2.8)
**Low bone mineral density**						
Self-harm and interpersonal violence	0.2 (0.2–0.3)	0.2 (0.2–0.3)	0.2 (0.0–0.5)	0.2 (−0.2 to 0.6)	0.9 (0.6–1.1)	−0.7 (−1.2 to −0.3)
Transport injuries	8.2 (7.1–8.8)	9.8 (8.4–10.6)	0.6 (0.3–0.8)	−0.3 (−0.5 to −0.1)	1.4 (0.8–2.0)	0.3 (−0.1 to 0.8)
Unintentional injuries	9.1 (7.7–10.9)	19.9 (14.0–23.4)	2.8 (1.8–3.7)	1.1 (0.1–2.1)	5.1 (2.2–8.0)	2.4 (1.8–2.9)
**Kidney dysfunction**						
Cardiovascular diseases	6.0 (4.7–7.3)	8.2 (6.2–10.2)	1.1 (1–1.2)	0.4 (0.3–0.6)	2.7 (2.6–2.9)	0.2 (−0.1 to 0.5)
Diabetes and kidney diseases	55.4 (52.8–57.3)	53.3 (52.1–54.6)	−0.2 (−0.2 to −0.1)	−0.4 (−0.5 to −0.3)	0.1 (0.1 to 0.2)	−0.2 (−0.3 to −0.2)
**High LDL cholesterol**						
Cardiovascular diseases	12.8 (9.4–17.3)	20.2 (14.3–27.4)	1.6 (1.5–1.6)	1.0 (0.9–1.1)	2.8 (2.7–2.9)	0.9 (0.8–1.0)

AAPC, average annual percent change; APC, annual percent change; DALYs, disability-adjusted life years; CI, confidence interval.

### Association between metabolic risk factor and SDI

There was a positive correlation between the SDI level and the number of six metabolic risk factors attributable DALYs, YLDs, deaths, and YLLs (Supplementary Table 6, Supplementary Figure 11). The SDI level showed a positive correlation with the age-standardized rate of DALYs for HBMI, LBMD, and HLDL, but a negative correlation with DALYs for HSBP and KDF. The SDI level showed a positive correlation with the age-standardized rate of deaths for HBMI, KDF, and HLDL, but a negative correlation with HSBP. The SDI level was positively correlated with the age-standardized rate of YLDs for all six metabolic risk factors except LBMD. The SDI level was positively correlated with the age-standardized rate of deaths YLLs for HBMI, and HLDL, but was negatively correlated with HFPG, HSBP, and KDF (Supplementary Table 6, Supplementary Figure 11).

### Overall burden ranking in Group of 20 (G20) countries

**Figure 2:**
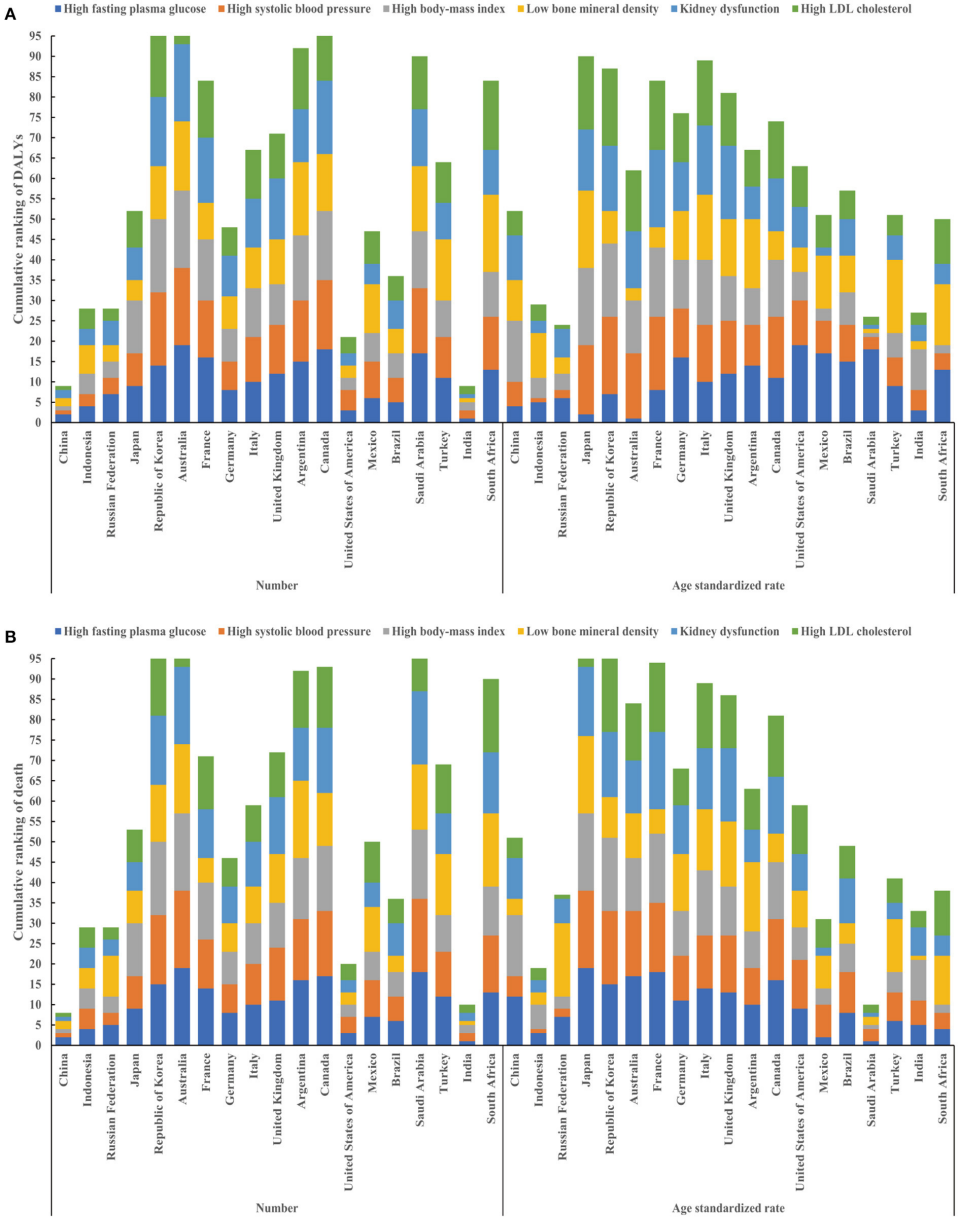
Cumulative ranking of DALYs and deaths attributable to six metabolic risk factors in 19 countries of Group 20 (excluding the European Union). Cumulative ranking of number (left) and age standardized rate (right) of DALYs attributable to six metabolic risk factors in 19 countries of Group 20 **(A)**; Cumulative ranking of number (left) and age standardized rate (right) of deaths attributable to six metabolic risk factors in 19 countries of Group 20 **(B)**. DALYs, disability-adjusted life years.

Among 19 countries of Group 20, in 2019, China ranked 1^st^, 1^st^, 1^st^, 2^nd^, 2^nd^, 2^nd^ for HSBP, HBMI, HLDL, HFPG, LBMD, KDF in terms of number of DALYs and ranked 1^st^, 1^st^, 1^st^, 1^st^, 2^nd^, 2^nd^ for HSBP, HBMI, HLDL, KDF, HFPG, LBMD in terms of number of deaths, respectively (Figure 2, Supplementary Table 7). The age-standardized rate of DALYs for HFPB, HSBP, HLDL, LBMD, KDF, HBMI ranked 4^th^, 6^th^, 6^th^, 10^th^, 11^th^, 15^th^, respectively (Figure 2, Supplementary Table 7). The age standardized rate of death for LBMD, HSBP, HLDL, KDF, HFPG, HBMI ranked 4^th^, 5^th^, 5^th^, 10^th^, 12^th^, 15^th^, respectively (Figure 2, Supplementary Table 7).

## Discussion

Our findings support the view that mitigation of metabolic risk factors provides a unique opportunity to achieve better health in the future. At first, 36 independent drivers of global health, especially 5 metabolic risk factors, were expected to deteriorate without comprehensive management, although most of them were forecast to improve by 2040 (19). In China, the population aged >65 is increasing rapidly and those aged >65 and >80 may reach 400 million and 150 million, respectively, before 2050 (20). Most importantly, our data support the view that risk factor driven disease burdens increased with age, reaching a peak for individuals aged >60.

Our results call for early and tight control of metabolic risk factors starting before the first peak (age 20–49) targeting adolescence. As a pivotal point in the life course characterized by openness to change, adolescence offers a unique window of opportunity to promote the adoption of a healthy lifestyle (diet and physical activity). However, this window of opportunity has largely been overlooked in behavior and policy research (21). Growing up in an era of “toxic” food environment, the current generation of adolescents may face unprecedented disease burdens from metabolic risk factors in their later life (22). The pace of change in the nutritional habits of adolescents poses a great threat to their health. National and individual efforts should identify key meanings and context of their food choices and seek to improve their food environments and choices by harnessing widely shared adolescent values that go beyond nutrition or health (23). Our findings also provided us an impetus to pharmacologically target key metabolic pathways linked to longevity before the second peak factors with the hope of delaying aging and ameliorating age-related diseases (24). On the one hand, a hallmark of aging is metabolism dysfunction, especially glucose homeostasis, negatively regulating energy metabolism and ultimately increasing the organism's susceptibility to disease. On the other hand, metabolic dysfunction occurs increasingly with age, including modulation of mitochondrial function, a decline in insulin sensitivity, and alterations in substrate utilization, which are associated with obesity, hyperglycemia, dyslipidemia, and insulin resistance (24).

These findings highlighted that male gender is a biological variable due to higher disease burden attributable to metabolic risk factors across all age groups. Free testosterone in men may be associated with a risk for major adverse cardiovascular events as men with lower sex hormone–binding globulin concentrations have a higher risk for myocardial infarction (25). The greater increase in disease burden among males will require more attention from health systems. Our data also support the view that menopause transition contributes to the increase in metabolic disease burden of old age (26), based on the fact that the AAPC in the rate of YLDs decreased for male patients but increased for female patients from 1 to 49 years before reaching a plateau. The reported findings underline the potential benefits of monitoring women's health during midlife and emphasize the critical window for early intervention strategies. Postmenopausal women, especially those younger than 60, should be fully advised on the benefits and harms of different types and timing of hormone therapy (27).

Our data imply that an integrative metabolic strategy should prioritize HBMI and LBMD due to the increased age-standardized rates of DALYs and death. Maintaining a BMI of 20.0–25.0 kg/m^2^ can help minimize all-cause mortality, and a minimum of 150 min weekly leisure moderate to vigorous physical activity was associated with the most health benefits (28, 29). Phentermine–topiramate and glucagon-like peptide-1 receptor agonists (GLP-1RA), particularly semaglutide, seem to be the best drugs for controlling weight in overweight and obese adults (30). Men and women with LBMD, consistent with osteoporosis or osteopenia, showed a significantly increased risk of fractures and mortality (31). Nonetheless, indiscriminate use of vitamin D supplementation to prevent fracture and osteoporosis seems should be avoided (32). Treatment with bisphosphonates or denosumab, and teriparatide or abaloparatide should be considered for individuals at high and imminent risk of fracture, respectively (33).

Integrative management in old individuals often requires the use of multiple drugs to treat concurrent dyslipidemia, diabetes, and hypertension, and those with metabolic syndrome should be tightly managed. Anti-hypertensive treatment targeting systolic blood-pressure 110–130 mmHg rather than 130–150 mmHg led to fewer incidences of cardiovascular events in Chinse older patients with hypertension (34). Compared with the gold standard angiotensin-converting enzyme inhibitor (enalapril) an angiotensin receptor-neprilysin inhibitor (sacubitril/valsartan) not only decreases the risk of cardiovascular death or heart failure hospitalization but also improves symptoms in patients with chronic heart failure with reduced ejection fraction (35). Clinical trials exploring the cardiovascular and renal outcomes of GLP-1RA and sodium-glucose cotransporter-2 inhibitors (SGLT2i) not only change the treatment paradigm of diabetes but also support a more holistic approach beyond glycemic control which emphasize on cardiac and reno-protective effects (36). Although statins, ezetimibe, and PCSK9 inhibitors are the standard of care for coronary artery disease, many novel LDL cholesterol–lowering drugs, such as inclisiran recently approved by the FDA, may transform the care of patients who are at risk of life-threatening coronary events (37, 38). KDF is harmful but treatable if individuals at risk are identified at an early stage. More individuals than ever before are experiencing KDF and nephrosclerosis-age-associated histologic changes, as observed in 2.7, 58, and 73% of biopsies from donors aged < 30, 60–69,70 plus, respectively (39). There is partial but limited success for kidney diseases treated with immunosuppressive agents, antihypertensives, and diuretics. Sodium-glucose co-transporter-2 (SGLT2) inhibition should be used as a foundational therapy for chronic kidney disease as SGLT2 inhibitors might substantially slow the progression of chronic kidney disease in people with type 2 diabetes (40, 41). New candidate therapeutic drugs targeting the glomerular filtration barrier may help to correct defects within or between cells of the glomerular filtration barrier and maintain its integrity (42). Most importantly, a mechanistic link has been observed among obesity, kidney dysfunction, and hypertension (43). Namely, excessive adiposity, which is a major driver of kidney diseases and prolonged obesity, and progressive renal injury, often leads to the development of treatment-resistant hypertension.

Our findings identified the precise diseases and risk factors that are most in need of attention. For example, our data showed that KDF-driven musculoskeletal disorders at level 2 cause and gout at level 3 cause. We also ascertained the trend of the contribution of risk factors, which may help formulate feasible and quantitative health policy. Analysis of population-attributable fractions indicated the increasing contribution of HSBP to cardiovascular diseases and of HFPG to neoplasms. A 5-mmHg decrement in systolic blood pressure decreases major cardiovascular events by ~10%, irrespective of previous cardiovascular disease (44). The risk of incident colorectal cancer associated with HFPG may help to identify people at high risk for future colorectal cancer (45). In addition, the evolutionary pattern of disability and death with societal development, as measured by the SDI, may help prioritize metabolic factors while formulating health policy. In addition to physical equipment, the government may also need to improve the availability of phentermine–topiramate and GLP-1 receptor agonists in adults who are overweight and obese as there is an increasing trend for HBMI and HLDL with the increase of SDI (46). In addition to the early detection of chronic kidney disease and interventions aiming to reduce urine protein excretion, more efforts are needed to delay and recover the progression of kidney function because there is increasing disability and decreasing death for KDF with the increase of SDI (47).

Our data revealed that China was the largest contributor to disease burden due to metabolic risk factors among 19 G20 countries. This presents a public health challenge and an opportunity for China to improve future health worldwide. Systematic management of individuals with metabolic syndromes must be stressed again as the age-standardized rate of DALYs and death in China ranked fourth to sixth among G20 countries for HFPG, HSBP, and HLDL. Although the age-standardized rate of DALYs and death in China ranked 10th to 15th for LBMD, KDF, and HBMI, this does not automatically imply that these indices are well-controlled. Since China gained more increments in life expectancy compared to other G20 countries, which may have led to a lower disease burden from age-related LBMD, KDF, and HBMI in the past three decades (2).

These findings are not entirely surprising if one follows the concept that metabolic aging is remolding future health structures. At the same time, these findings are interesting as they foster the concept of prevention always triumphs over remediation during adolescence and integrative strategy for key risk factors and individuals. Nonetheless, these findings are subject to two major limitations. First, the study did not include urban-rural and province-level data, which may be useful to formulate provincial-, urban-, and rural-specific health policies. Second, the study is subject to the same limitation as the GBD 2019 studies (2, 3).

## Data availability statement

The original contributions presented in the study are included in the article/Supplementary material, further inquiries can be directed to the corresponding authors.

## Ethics statement

This manuscript was produced as part of the GBD Collaborator Network in accordance with the GBD Protocol (IHME ID. 1775-GBD2019-012021). Data released from the Global Health Data Exchange query did not require informed patient consent. This study used an anonymized publicly available data set with no identifiable information about the survey participants.

## Author contributions

JC, KHH, YZJ, L-LL, SM, HS, A-MW, and SWX provided data or critical feedback on data sources. YZJ and A-MW developed methods of computational machinery and managed the estimation or publication process. AB, JC, EC, XQF, KHH, KAH, YZJ, L-LL, SM, FW, ZYW, DZW, A-MW, SWX, and ZYZ provided critical feedback on methods or results. AB, JC, EC, KAH, JJH, YZJ, L-LL, YMS, L-ST, FW, ZYW, DZW, A-MW, and CWZ drafted the work or revising it critically for important intellectual content. All authors contributed to the article and approved the submitted version.
