# Supplementary material for: The temporal trend of disease burden attributable to metabolic risk factors in China, 1990–2019: An analysis of the Global Burden of Disease study
Source: Front Nutr. 2023 Jan 4;9:1035439. doi: 10.3389/fnut.2022.1035439 (PMC9846330; doi:10.3389/fnut.2022.1035439)
Supplement: Supplementary file 1 [file Data_Sheet_1.DOCX]

**Supplementary materials**

# **Table of content**

**Case definition**

**The GBD metabolic risk factor attributable cause hierarchy**

**Estimation of six metabolic risk factors**

**Supplementary figure**

**sFigure 1**. AAPC in rate of DALYs attributable to six metabolic risk factors across 20 age groups, 1990-2019.

**sFigure 2**. AAPC in rate of deaths attributable to six metabolic risk factors across 20 age groups, 1990-2019.

**sFigure 3**. Trend in number of DALYs and deaths attributable to metabolic risk factors across 15-94 years, 1990 to 2019

**sFigure 4**. Trend in number of DALYs attributable to six metabolic risk factors across 4 age groups (age 15-94), 1990 to 2019

**sFigure 5**. Trend in number of deaths attributable to six metabolic risk factors across 4 age groups (age 15-94), 1990 to 2019

**sFigure 6**. The number and age standardized rate of DALYs and deaths attributable to six metabolic risk factors according to gender in 2019

**sFigure 7**. The age-standardized rate of YLDs and YLLs attributable to six metabolic risk factors according to gender, 2019.

**sFigure 8**. Trend in number and age-standardized rate of DALYs and deaths attributable to six metabolic risk factors, 1990-2019

**sFigure 9**. Trend of population attributable fraction on level 2 causes attributable to six metabolic risk factors in terms of age-standardized rate of DALYs, 1990-2019

**sFigure 10**. Trend of population attributable fraction on level 2 causes attributable to six metabolic risk factors in terms of age-standardized rate of death, 1990-2019

**sFigure 11**. Correlation of SDI with age-standardized rate of DALYs (A), YLDs (B), deaths (C) and YLLs (D) attributable to six metabolic risk factors, 1990-2019

**Supplementary tables**

**sTable 1**. Trend in number and age-standardized rate of DALYs and deaths attributable to six metabolic risk factors for male and female in China, 1990-2019

**sTable 2**. Trend in number and age-standardized rate of DALYs and deaths for level 3 causes attributable to six metabolic risk factors in China, 1990-2019

**sTable 3**. Trend in number and age-standardized rate of DALYs and deaths for level 4 causes attributable to six metabolic risk factors in China, 1990-2019

**sTable 4**. Trend in number and age-standardized rate of DALYs and deaths for the most detailed causes attributable to six metabolic risk factors in China, 1990-2019

**sTable 5**. Population attributable fractions (PAFs) in DALYs, YLDs, YLLs and deaths of level 2 causes attributable to six metabolic risk factors in China, 2019

**sTable 6**. Correlation between SDI and DALYs, YLLs, YLDs and deaths from 1990 to 2019

**sTable 7**. The value and ranking in the number and age standardized rate of DALYs and deaths attributable to six metabolic risk factors in 19 countries of Group 20 except European Union

# **Case definition**

**High fasting plasma glucose (FPG)** was measured as the mean FPG in the population, where FPG was a continuous exposure in mmol/l. Since FPG is a continuum, high FPG was defined as any level of TMREL (4.8-5.4 mmol/L).

**High low-density lipoprotein (HLDL)** Blood concentration of low-density lipoprotein (LDL) was modelled with a uniform distribution between 0·7 and 1·3 mmol/L.

**High systolic blood pressure (HSBP)** Brachial systolic blood pressure was modelled ranging from 110 to 115 mmHg.

**High body-mass index (BMI)** for adults (ages 20+) is defined as BMI greater than 20 to 25 kg/m2. High BMI for children (ages 1–19) is defined as being overweight or obese based on International Obesity Task Force standards.

**Low Bone mineral density (LBMD)** is measured in terms of the difference between BMD of a population and the 99^th^ percentile of a reference population at the same age and sex (theoretical minimum-risk exposure level, TMREL). The burden attributed to low bone mineral density is estimated for adults 20 years and older. Bone mineral density (BMD) is a continuous variable measured by dual-X-ray-absorptiometry (DXA) at the femoral neck (FN) and is presented in g/cm2 after standardizing for the brand of densitometer (sBMD).

**Kidney dysfunction (KDF)** is divided into four categories of renal function defined by urinary albumin to creatinine ratio (ACR) and estimated glomerular filtration rate (eGFR). (1) Albuminuria with preserved eGFR (ACR >30 mg/g & eGFR >=60 ml/min/1.73m^2^); this corresponds to stages 1 and 2 chronic kidney disease (CKD) in the Kidney Disease Improving Global Outcomes (KDIGO) classification. (2) CKD stage 3 (eGFR of 30-59 ml/min/1.73m^2^). (2) CKD stage 4 (eGFR of 15-29 ml/min/1.73m^2^). (4) CKD stage 5 (eGFR <15ml/min/1.73m^2^, not (yet) on renal replacement therapy).

# **The GBD metabolic risk factor attributable cause hierarchy**

The 23, 66, 61, 108 metabolic risk factor attributable level 2, 3, 4, most detailed risk-cause pairs.

| **Risk** | **Cause** |
| --- | --- |
| **23 level 2 risk-cause pair** | |
| High fasting plasma glucose | Neoplasms |
|  | Cardiovascular diseases |
|  | Neurological disorders |
|  | Sense organ diseases |
|  | Respiratory infections and tuberculosis |
|  | Diabetes and kidney diseases |
|  | Cardiovascular diseases |
|  | Diabetes and kidney diseases |
| High body-mass index | Neoplasms |
|  | Cardiovascular diseases |
|  | Chronic respiratory diseases |
|  | Digestive diseases |
|  | Neurological disorders |
|  | Musculoskeletal disorders |
|  | Sense organ diseases |
|  | Diabetes and kidney diseases |
| Low bone mineral density | Transport injuries |
|  | Unintentional injuries |
|  | Self-harm and interpersonal violence |
| Kidney dysfunction | Cardiovascular diseases |
|  | Musculoskeletal disorders |
|  | Diabetes and kidney diseases |
| High LDL cholesterol | Cardiovascular diseases |
| **66 level 3 risk-cause pair** | |
| High fasting plasma glucose | Tuberculosis |
|  | Liver cancer |
|  | Tracheal, bronchus, and lung cancer |
|  | Breast cancer |
|  | Colon and rectum cancer |
|  | Pancreatic cancer |
|  | Ovarian cancer |
|  | Bladder cancer |
|  | Ischemic heart disease |
|  | Stroke |
|  | Peripheral artery disease |
|  | Alzheimer's disease and other dementias |
|  | Diabetes mellitus |
|  | Chronic kidney disease |
|  | Blindness and vision loss |
| High systolic blood pressure | Rheumatic heart disease |
|  | Ischemic heart disease |
|  | Stroke |
|  | Hypertensive heart disease |
|  | Cardiomyopathy and myocarditis |
|  | Atrial fibrillation and flutter |
|  | Aortic aneurysm |
|  | Peripheral artery disease |
|  | Endocarditis |
|  | Non-rheumatic valvular heart disease |
|  | Chronic kidney disease |
|  | Other cardiovascular and circulatory diseases |
| High body-mass index | Esophageal cancer |
|  | Liver cancer |
|  | Breast cancer |
|  | Uterine cancer |
|  | Colon and rectum cancer |
|  | Gallbladder and biliary tract cancer |
|  | Pancreatic cancer |
|  | Ovarian cancer |
|  | Kidney cancer |
|  | Thyroid cancer |
|  | Non-Hodgkin lymphoma |
|  | Multiple myeloma |
|  | Leukemia |
|  | Ischemic heart disease |
|  | Stroke |
|  | Hypertensive heart disease |
|  | Atrial fibrillation and flutter |
|  | Asthma |
|  | Gallbladder and biliary diseases |
|  | Alzheimer's disease and other dementias |
|  | Diabetes mellitus |
|  | Chronic kidney disease |
|  | Osteoarthritis |
|  | Low back pain |
|  | Gout |
|  | Blindness and vision loss |
| Low bone mineral density | Road injuries |
|  | Other transport injuries |
|  | Falls |
|  | Exposure to mechanical forces |
|  | Animal contact |
|  | Interpersonal violence |
| Kidney dysfunction | Ischemic heart disease |
|  | Stroke |
|  | Peripheral artery disease |
|  | Chronic kidney disease |
|  | Gout |
| High LDL cholesterol | Ischemic heart disease |
|  | Stroke |
| **61 level 4 risk-cause pair** | |
| High fasting plasma glucose | Ischemic stroke |
|  | Intracerebral hemorrhage |
|  | Subarachnoid hemorrhage |
|  | Glaucoma |
|  | Cataract |
|  | Drug-susceptible tuberculosis |
|  | Multidrug-resistant tuberculosis without extensive drug resistance |
|  | Extensively drug-resistant tuberculosis |
|  | Latent tuberculosis infection |
|  | Diabetes mellitus type 1 |
|  | Diabetes mellitus type 2 |
|  | Liver cancer due to NASH |
|  | Chronic kidney disease due to diabetes mellitus type 1 |
|  | Chronic kidney disease due to diabetes mellitus type 2 |
|  | Liver cancer due to other causes |
| High systolic blood pressure | Ischemic stroke |
|  | Intracerebral hemorrhage |
|  | Subarachnoid hemorrhage |
|  | Chronic kidney disease due to hypertension |
|  | Chronic kidney disease due to glomerulonephritis |
|  | Chronic kidney disease due to other and unspecified causes |
|  | Other cardiomyopathy |
|  | Non-rheumatic calcific aortic valve disease |
|  | Chronic kidney disease due to diabetes mellitus type 1 |
|  | Chronic kidney disease due to diabetes mellitus type 2 |
| High body-mass index | Liver cancer due to hepatitis B |
|  | Liver cancer due to hepatitis C |
|  | Liver cancer due to alcohol use |
|  | Ischemic stroke |
|  | Intracerebral hemorrhage |
|  | Subarachnoid hemorrhage |
|  | Chronic kidney disease due to hypertension |
|  | Chronic kidney disease due to glomerulonephritis |
|  | Chronic kidney disease due to other and unspecified causes |
|  | Cataract |
|  | Acute lymphoid leukemia |
|  | Chronic lymphoid leukemia |
|  | Acute myeloid leukemia |
|  | Chronic myeloid leukemia |
|  | Other leukemia |
|  | Diabetes mellitus type 2 |
|  | Chronic kidney disease due to diabetes mellitus type 2 |
|  | Osteoarthritis hip |
|  | Osteoarthritis knee |
|  | Liver cancer due to other causes |
| Low bone mineral density | Pedestrian road injuries |
|  | Cyclist road injuries |
|  | Motorcyclist road injuries |
|  | Motor vehicle road injuries |
|  | Other road injuries |
|  | Other exposure to mechanical forces |
|  | Non-venomous animal contact |
|  | Physical violence by other means |
| Kidney dysfunction | Ischemic stroke |
|  | Intracerebral hemorrhage |
|  | Chronic kidney disease due to hypertension |
|  | Chronic kidney disease due to glomerulonephritis |
|  | Chronic kidney disease due to other and unspecified causes |
|  | Chronic kidney disease due to diabetes mellitus type 1 |
|  | Chronic kidney disease due to diabetes mellitus type 2 |
| High LDL cholesterol | Ischemic stroke |
| **108 most detailed risk-cause pair** | |
| High fasting plasma glucose | Tracheal, bronchus, and lung cancer |
|  | Breast cancer |
|  | Colon and rectum cancer |
|  | Pancreatic cancer |
|  | Ovarian cancer |
|  | Bladder cancer |
|  | Ischemic heart disease |
|  | Ischemic stroke |
|  | Intracerebral hemorrhage |
|  | Subarachnoid hemorrhage |
|  | Peripheral artery disease |
|  | Alzheimer's disease and other dementias |
|  | Glaucoma |
|  | Cataract |
|  | Drug-susceptible tuberculosis |
|  | Multidrug-resistant tuberculosis without extensive drug resistance |
|  | Extensively drug-resistant tuberculosis |
|  | Latent tuberculosis infection |
|  | Diabetes mellitus type 1 |
|  | Diabetes mellitus type 2 |
|  | Liver cancer due to NASH |
|  | Chronic kidney disease due to diabetes mellitus type 1 |
|  | Chronic kidney disease due to diabetes mellitus type 2 |
|  | Liver cancer due to other causes |
|  | Total burden related to Non-alcoholic fatty liver disease (NAFLD) |
|  | Total cancers |
| High systolic blood pressure | Rheumatic heart disease |
|  | Ischemic heart disease |
|  | Ischemic stroke |
|  | Intracerebral hemorrhage |
|  | Subarachnoid hemorrhage |
|  | Hypertensive heart disease |
|  | Atrial fibrillation and flutter |
|  | Aortic aneurysm |
|  | Peripheral artery disease |
|  | Endocarditis |
|  | Chronic kidney disease due to hypertension |
|  | Chronic kidney disease due to glomerulonephritis |
|  | Chronic kidney disease due to other and unspecified causes |
|  | Other cardiomyopathy |
|  | Non-rheumatic calcific aortic valve disease |
|  | Chronic kidney disease due to diabetes mellitus type 1 |
|  | Chronic kidney disease due to diabetes mellitus type 2 |
|  | Other cardiovascular and circulatory diseases |
| High body-mass index | Esophageal cancer |
|  | Liver cancer due to hepatitis B |
|  | Liver cancer due to hepatitis C |
|  | Liver cancer due to alcohol use |
|  | Breast cancer |
|  | Uterine cancer |
|  | Colon and rectum cancer |
|  | Gallbladder and biliary tract cancer |
|  | Pancreatic cancer |
|  | Ovarian cancer |
|  | Kidney cancer |
|  | Thyroid cancer |
|  | Non-Hodgkin lymphoma |
|  | Multiple myeloma |
|  | Ischemic heart disease |
|  | Ischemic stroke |
|  | Intracerebral hemorrhage |
|  | Subarachnoid hemorrhage |
|  | Hypertensive heart disease |
|  | Atrial fibrillation and flutter |
|  | Asthma |
|  | Gallbladder and biliary diseases |
|  | Alzheimer's disease and other dementias |
|  | Chronic kidney disease due to hypertension |
|  | Chronic kidney disease due to glomerulonephritis |
|  | Chronic kidney disease due to other and unspecified causes |
|  | Low back pain |
|  | Gout |
|  | Cataract |
|  | Acute lymphoid leukemia |
|  | Chronic lymphoid leukemia |
|  | Acute myeloid leukemia |
|  | Chronic myeloid leukemia |
|  | Other leukemia |
|  | Diabetes mellitus type 2 |
|  | Chronic kidney disease due to diabetes mellitus type 2 |
|  | Osteoarthritis hip |
|  | Osteoarthritis knee |
|  | Liver cancer due to other causes |
|  | Total burden related to hepatitis B |
|  | Total burden related to hepatitis C |
|  | Total cancers |
| Low bone mineral density | Pedestrian road injuries |
|  | Cyclist road injuries |
|  | Motorcyclist road injuries |
|  | Motor vehicle road injuries |
|  | Other road injuries |
|  | Other transport injuries |
|  | Falls |
|  | Other exposure to mechanical forces |
|  | Non-venomous animal contact |
|  | Physical violence by other means |
| Kidney dysfunction | Ischemic heart disease |
|  | Ischemic stroke |
|  | Intracerebral hemorrhage |
|  | Peripheral artery disease |
|  | Chronic kidney disease due to hypertension |
|  | Chronic kidney disease due to glomerulonephritis |
|  | Chronic kidney disease due to other and unspecified causes |
|  | Gout |
|  | Chronic kidney disease due to diabetes mellitus type 1 |
|  | Chronic kidney disease due to diabetes mellitus type 2 |
| High LDL cholesterol | Ischemic heart disease |
|  | Ischemic stroke |

# **Estimation of six metabolic risk factors**

(Adapted from: GBD 2019 Risk Factors Collaborators, Global burden of 87 risk factors in 204 countries and territories, 1990-2019: a systematic analysis for the Global Burden of Disease Study 2019, Lancet. 2020 Oct 17;396(10258):1223-1249)

**Population-attributable fraction (PAF)**

For continuous risk factors, the formula for the PAF is defined as:
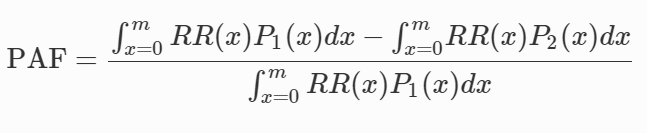


Where RR(x) is the RR of a specific disease at exposure level x, P1(x) is the population distribution of the exposure, P2(x) is the minimum theoretical exposure distribution, and m is the maximum exposure level. The minimum theoretical exposure is the counterfactual condition of exposure, which has been previously defined for each risk factor.

For categorical risk factor, the formula for PAF is:


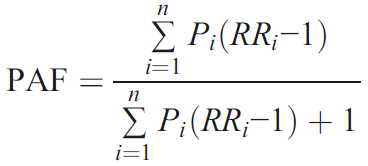


where i is exposure level, RRi is RR for exposure level i, and Pi is the prevalence of exposure level i.

# **Supplementary figure**


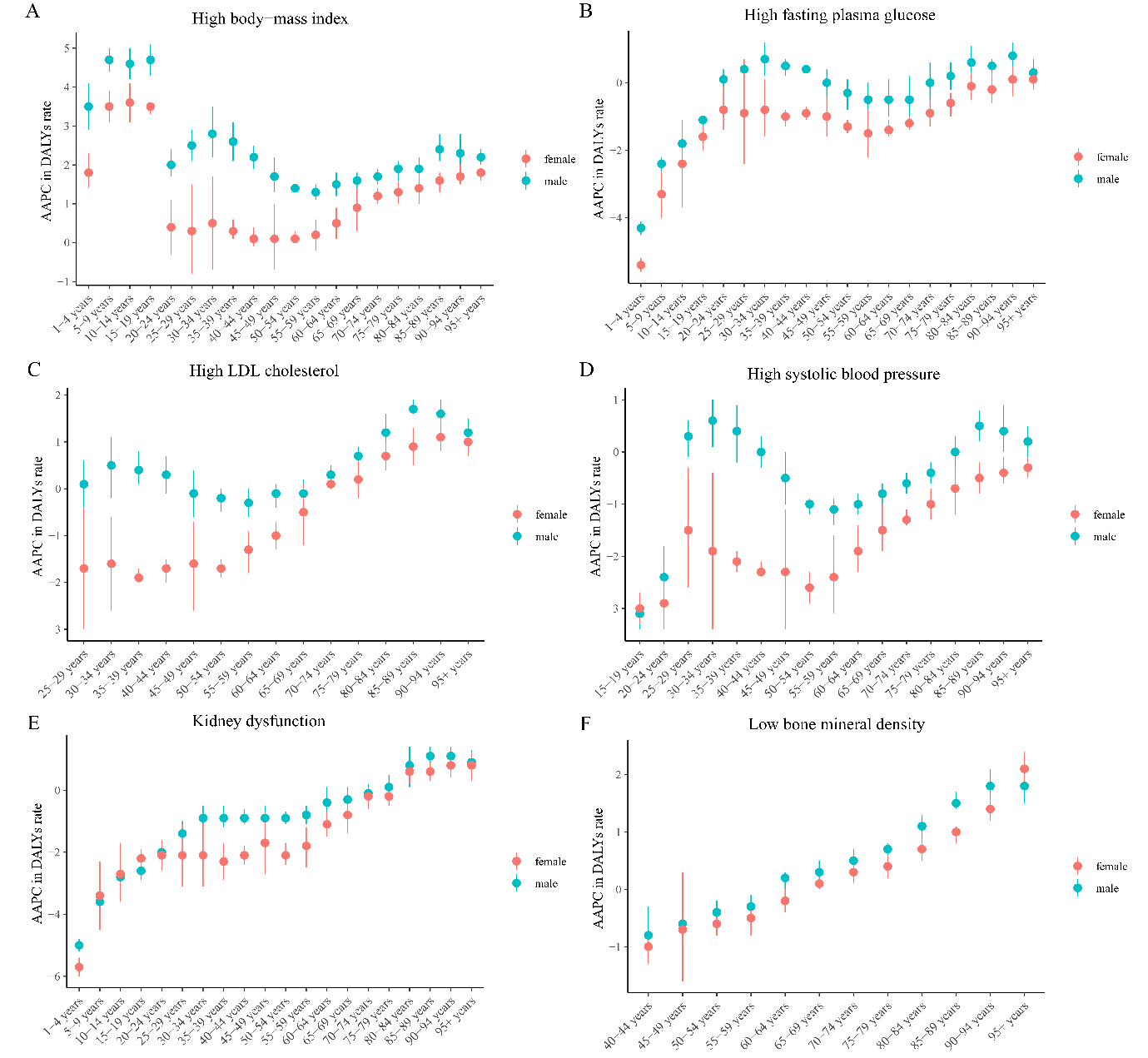


**sFigure 1**. AAPC in rate of DALYs attributable to six metabolic risk factors across 20 age groups, 1990-2019.

High body-mass index (A), high fasting plasma glucose (B), high LDL cholesterol (C), high systolic blood pressure (D), kidney dysfunction (E), low bone mineral density (F); AAPC, average annual percent change; DALYs, disability-adjusted life years.


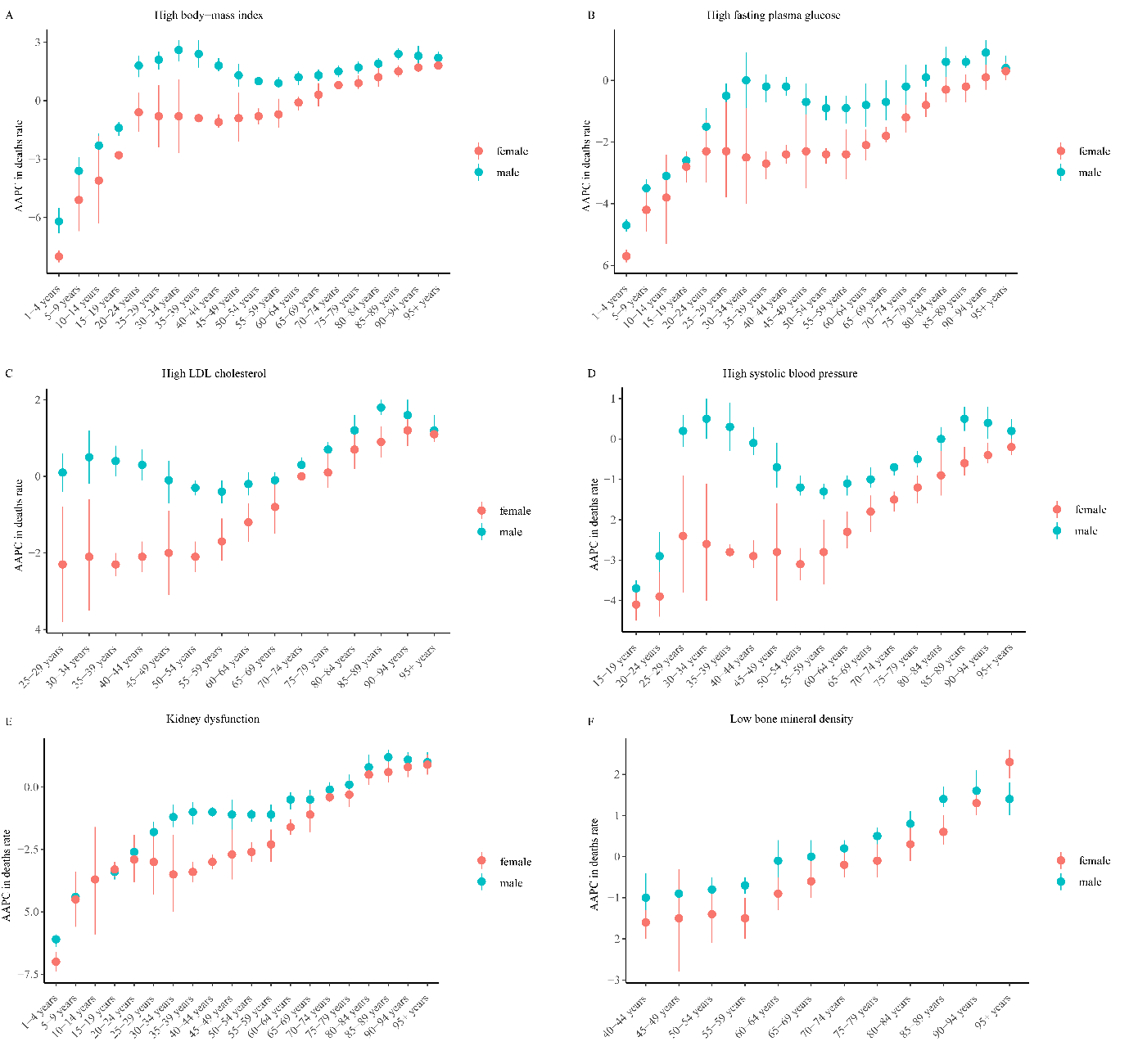


**sFigure 2**. AAPC in rate of deaths attributable to six metabolic risk factors across 20 age groups, 1990-2019.

High body-mass index (A), high fasting plasma glucose (B), high LDL cholesterol (C), high systolic blood pressure (D), kidney dysfunction (E), low bone mineral density (F); AAPC, average annual percent change; DALYs, disability-adjusted life years.


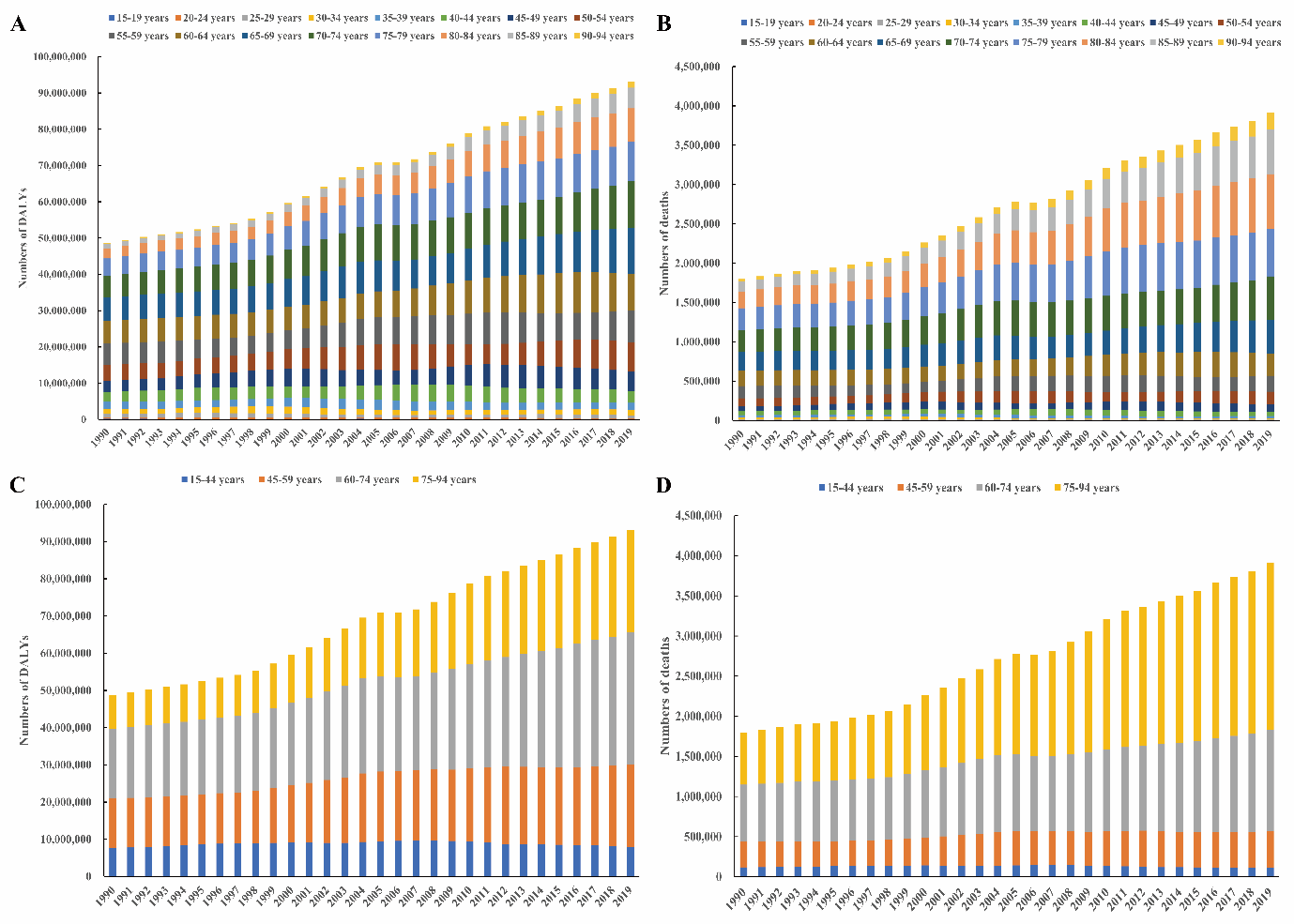


**sFigure 3**. Trend in number of DALYs and deaths attributable to metabolic risk factors across 15-94 years, 1990 to 2019

Trend in number of DALYs attributable to metabolic risk factors across 16 age groups (age 15-94), 1990 to 2019 (A), trend in number of deaths attributable to metabolic risk factors across 16 age groups (age 15-94), 1990 to 2019 (B), trend in number of DALYs attributable to metabolic risk factors across 4 age groups (age 15-94), 1990 to 2019 (C), trend in number of deaths attributable to metabolic risk factors across 4 age groups (age 15-94), 1990 to 2019 (D). DALYs, disability-adjusted life years.


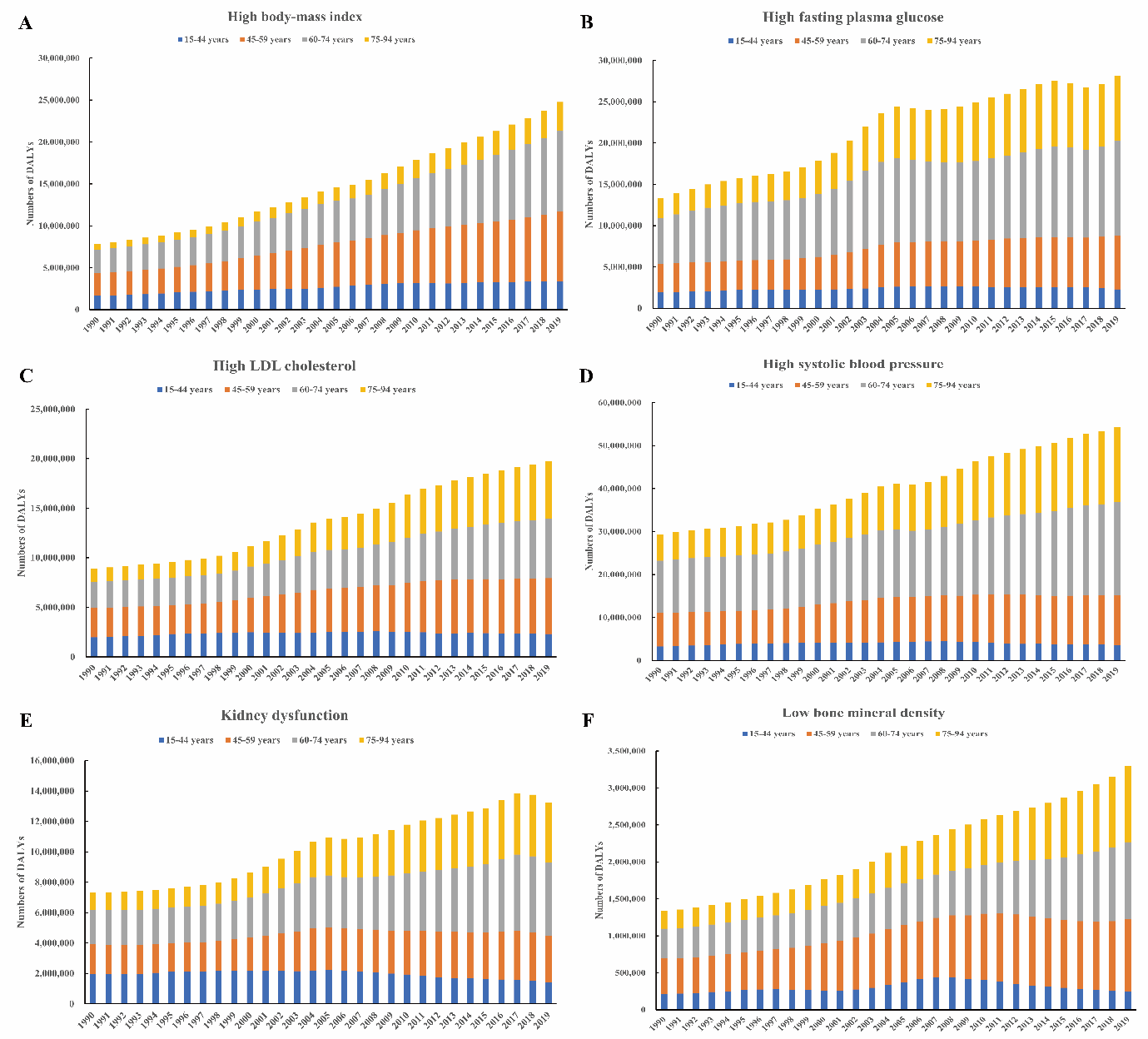


**sFigure 4**. Trend in number of DALYs attributable to six metabolic risk factors across 4 age groups (age 15-94), 1990 to 2019

High body-mass index (A), high fasting plasma glucose (B), high LDL cholesterol (C), high systolic blood pressure (D), kidney dysfunction (E), low bone mineral density (F); DALYs, disability-adjusted life years.


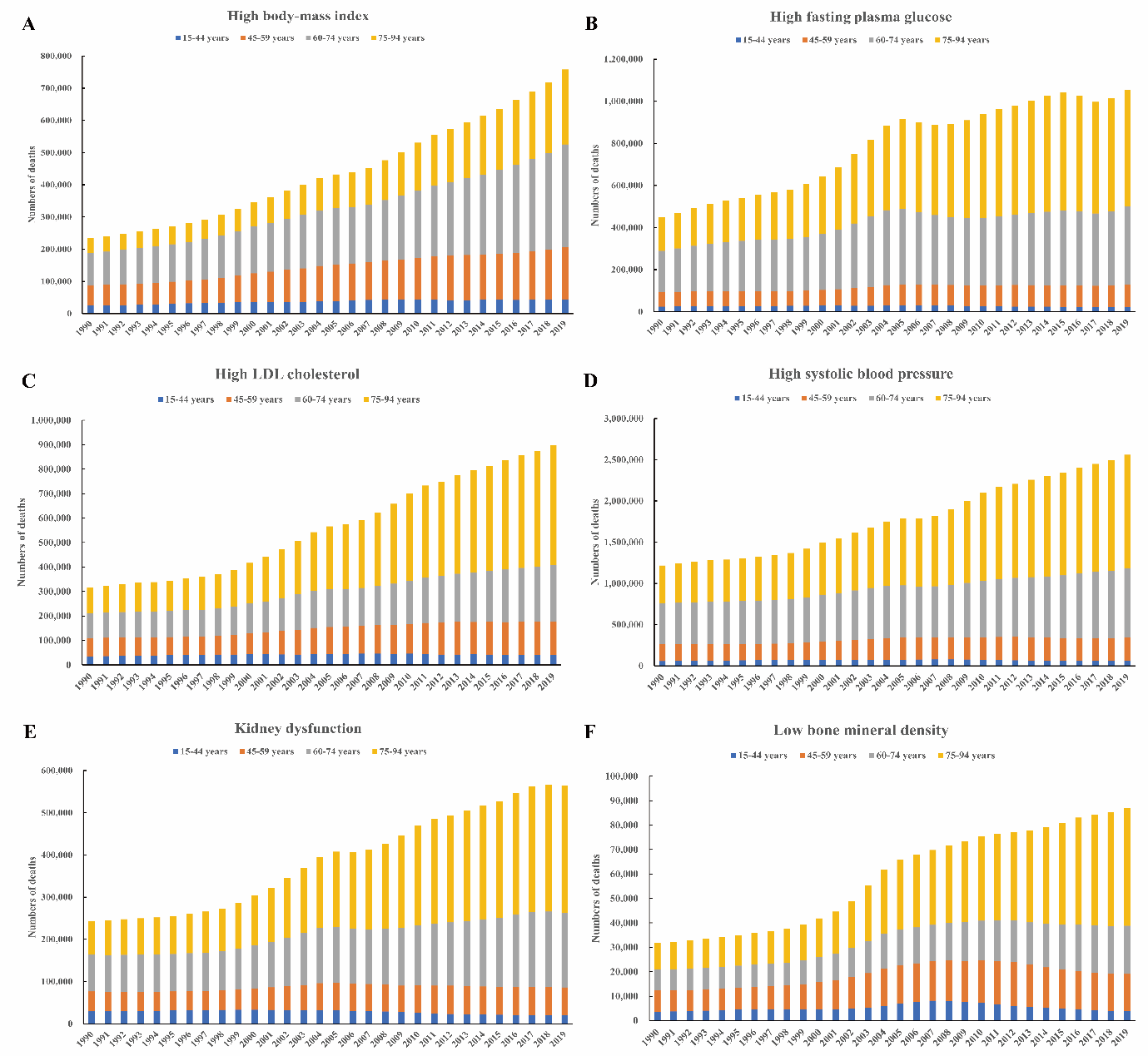


**sFigure 5**. Trend in number of deaths attributable to six metabolic risk factors across 4 age groups (age 15-94), 1990 to 2019

High body-mass index (A), high fasting plasma glucose (B), high LDL cholesterol (C), high systolic blood pressure (D), kidney dysfunction (E), low bone mineral density (F).


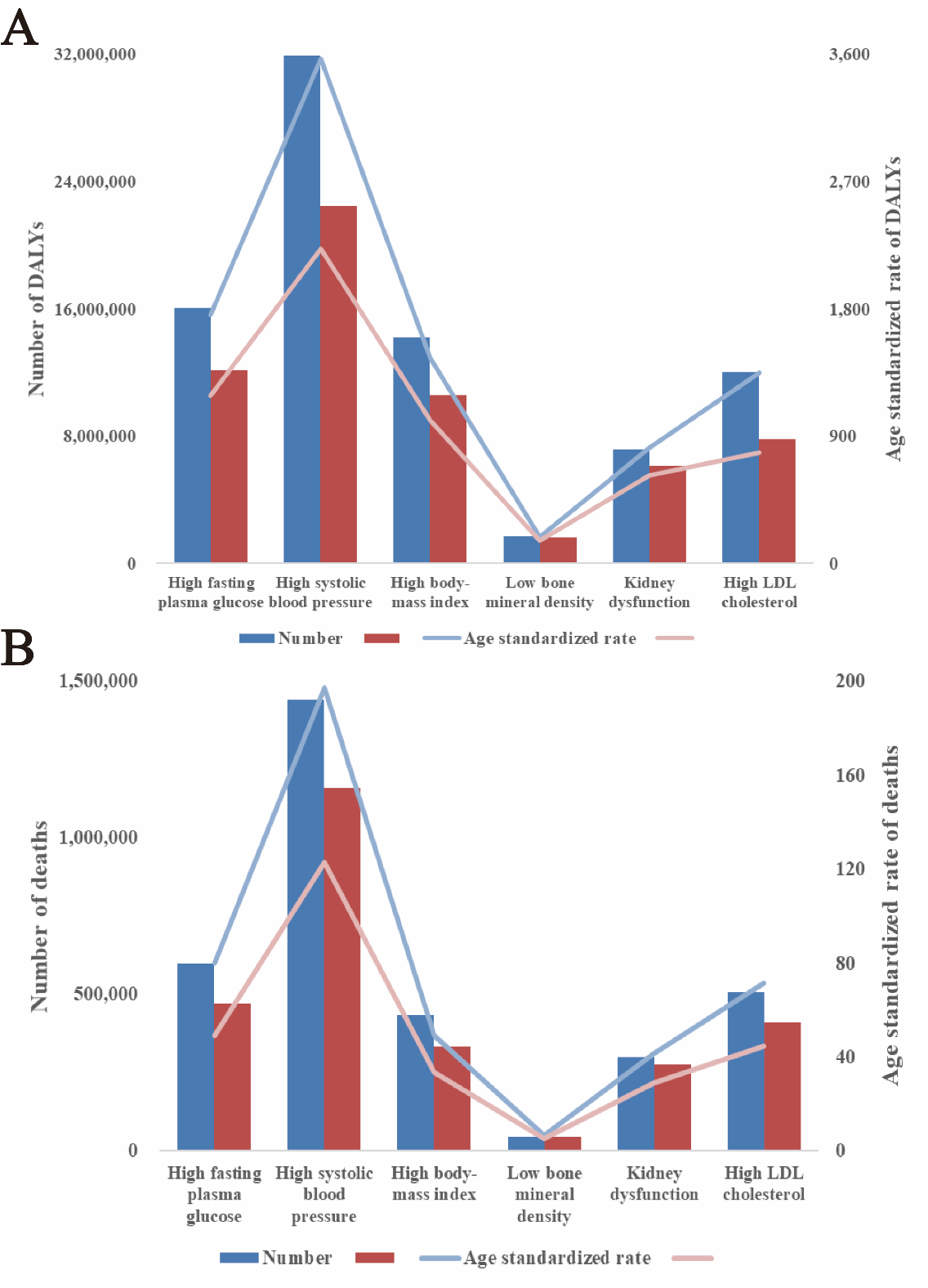


**sFigure 6**. The number and age standardized rate of DALYs and deaths attributable to six metabolic risk factors according to gender in 2019. Blue: male; Red: female.

The number (left) and age standardized rate (right) of DALYs attributable to six metabolic risk factors according to gender in 2019 (A), the number (left) and age standardized rate (right) of deaths attributable to six metabolic risk factors according to gender in 2019 (B); DALYs, disability-adjusted life years.


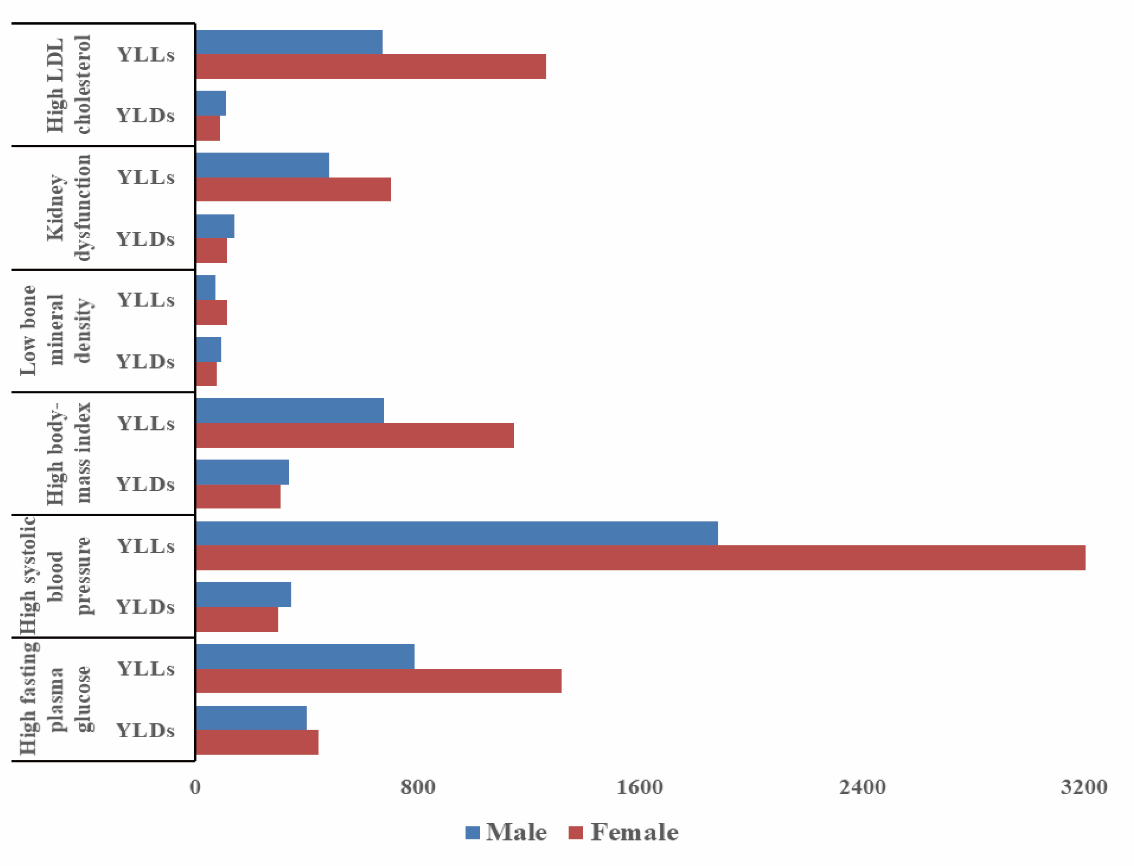


**sFigure 7**. The age-standardized rate of YLDs and YLLs attributable to six metabolic risk factors according to gender, 2019.

YLDs, years lived with disability; YLLs, years of life lost.


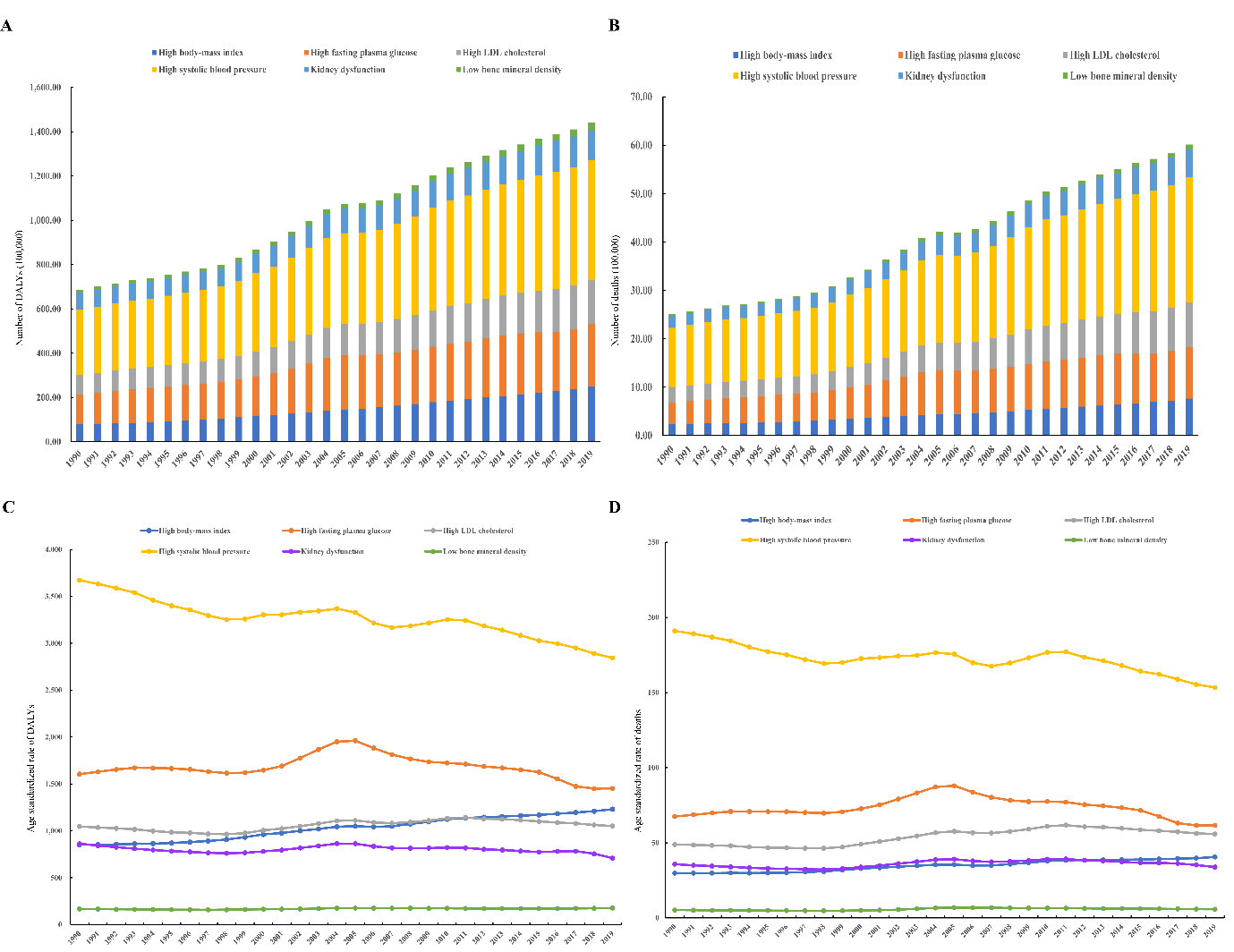


**sFigure 8**. Trend in number and age-standardized rate of DALYs and deaths attributable to six metabolic risk factors, 1990-2019

Trend in number of DALYs attributable to six metabolic risk factors, 1990-2019 (A), trend in number of deaths attributable to six metabolic risk factors, 1990-2019 (B), trend in rate of DALYs attributable to six metabolic risk factors, 1990-2019 (C), trend in rate of deaths attributable to six metabolic risk factors, 1990-2019 (D); DALYs, disability-adjusted life years.


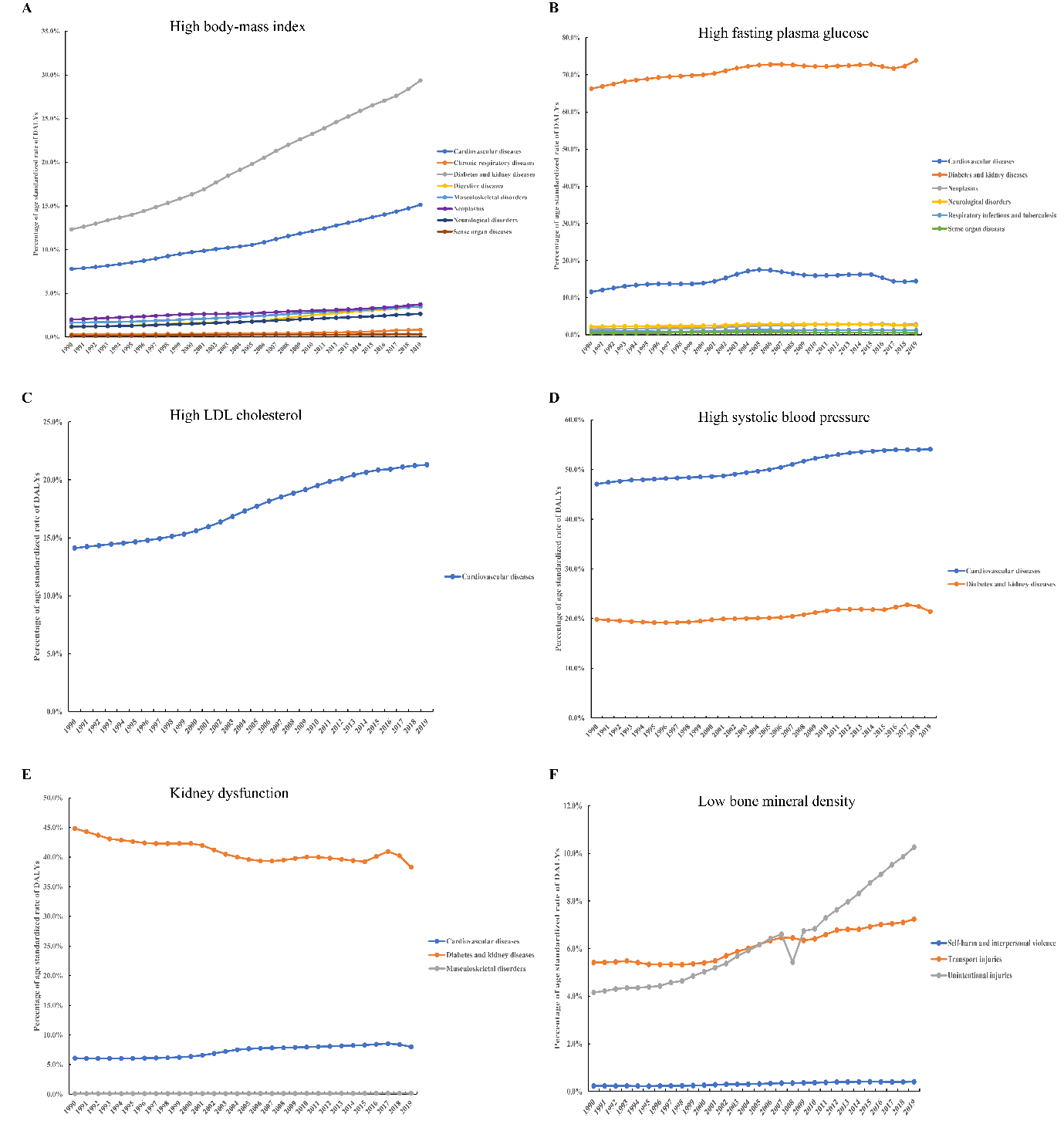


**sFigure 9**. Trend of population attributable fraction on level 2 causes attributable to six metabolic risk factors in terms of age-standardized rate of DALYs, 1990-2019

Trend of population attributable fraction on level 2 causes attributable to high body-mass index in terms of age-standardized rate of DALYs, 1990-2019(A), trend of population attributable fraction on level 2 causes attributable to high fasting plasma glucose in terms of age-standardized rate of DALYs, 1990-2019(B), trend of population attributable fraction on level 2 causes attributable to high LDL cholesterol in terms of age-standardized rate of DALYs, 1990-2019(C), trend of population attributable fraction on level 2 causes attributable to high systolic blood pressure in terms of age-standardized rate of DALYs, 1990-2019(D), trend of population attributable fraction on level 2 causes attributable to kidney dysfunction in terms of age-standardized rate of DALYs, 1990-2019(E), trend of population attributable fraction on level 2 causes attributable to low bone mineral density in terms of age-standardized rate of DALYs, 1990-2019(F).


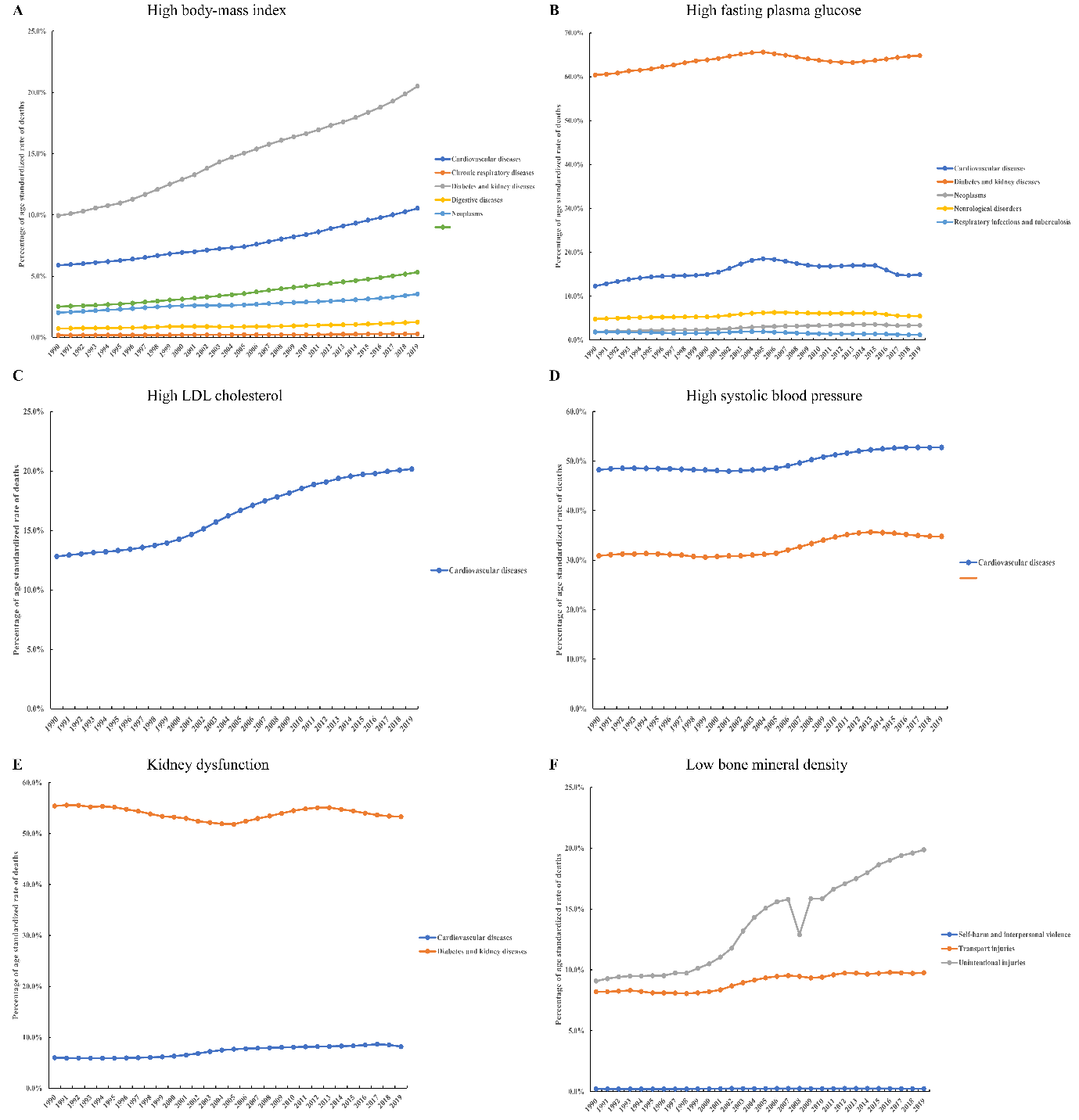


**sFigure 10**. Trend of population attributable fraction on level 2 causes attributable to six metabolic risk factors in terms of age-standardized rate of death, 1990-2019

Trend of population attributable fraction on level 2 causes attributable to high body-mass index in terms of age-standardized rate of death, 1990-2019(A), trend of population attributable fraction on level 2 causes attributable to high fasting plasma glucose in terms of age-standardized rate of death, 1990-2019(B), trend of population attributable fraction on level 2 causes attributable to high LDL cholesterol in terms of age-standardized rate of death, 1990-2019(C), trend of population attributable fraction on level 2 causes attributable to high systolic blood pressure in terms of age-standardized rate of death, 1990-2019(D), trend of population attributable fraction on level 2 causes attributable to kidney dysfunction in terms of age-standardized rate of death, 1990-2019(E), trend of population attributable fraction on level 2 causes attributable to low bone mineral density in terms of age-standardized rate of death, 1990-2019(F).


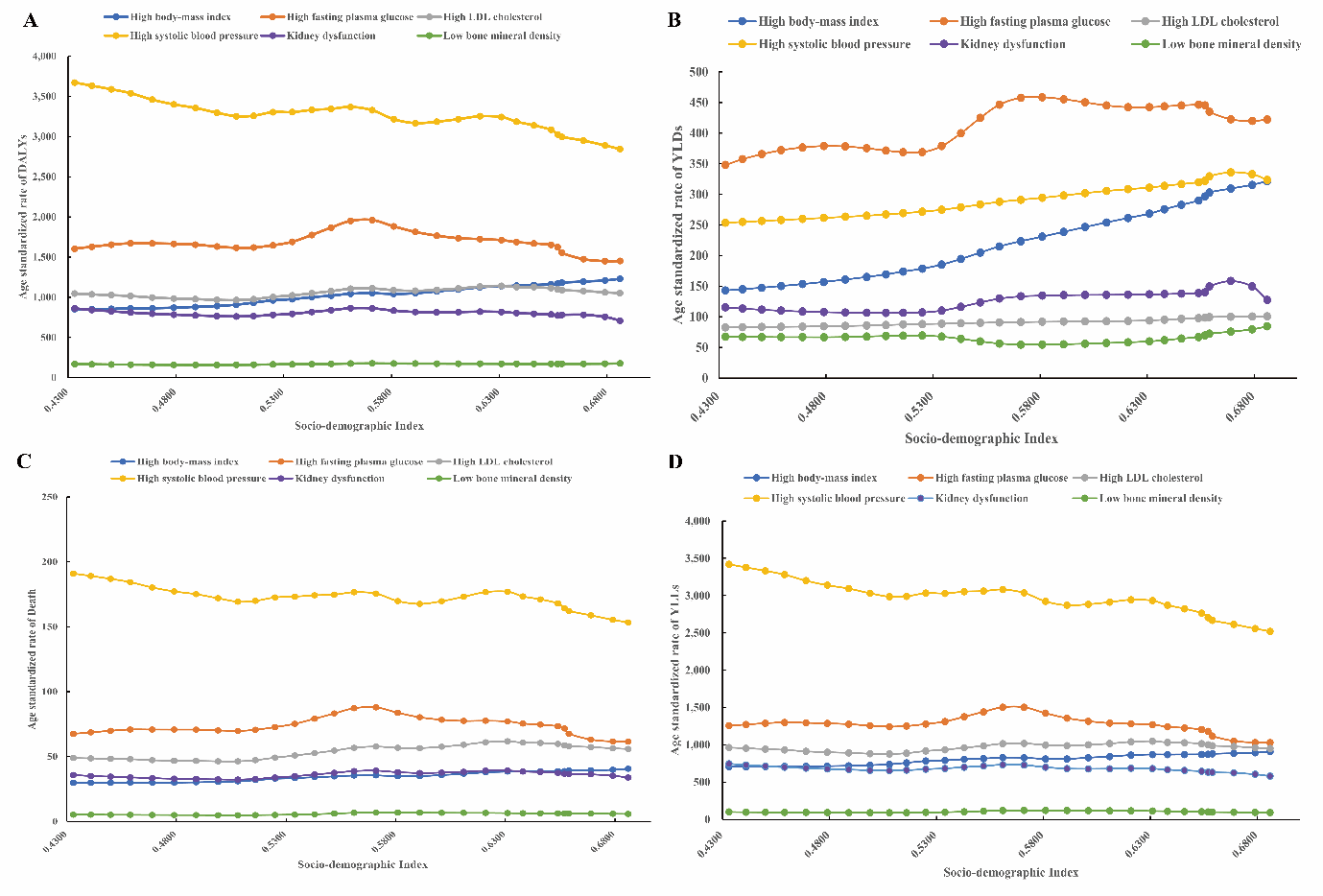


**sFigure 11**. Correlation of SDI with age-standardized rate (per 100,000 population) of DALYs (A), YLDs (B), deaths (C) and YLLs (D) attributable to six metabolic risk factors, 1990-2019

Abbreviation: SDI, Sociodemographic index; DALYs, disability-adjusted life years; YLDs, years lived with disability; YLLs, years of life lost

# **Supplementary tables**

**sTable 1**. Trend in number and age-standardized rate of DALYs and deaths attributable to six metabolic risk factors for male and female in China, 1990-2019

|  | DALYs, N (95% UI) | | | Deaths, N (95% UI) | | |
| --- | --- | --- | --- | --- | --- | --- |
|  | 1990 | 2019 | AAPC (95% CI) | 1990 | 2019 | AAPC (95% CI) |
| **Number** | | | | | | |
| **Male** | | | | | | |
| High fasting plasma glucose | 6,849,477(5,315,768 to 8,577,153) | 16,079,736(12,087,035 to 20,582,965) | 2·9(2·4 to 3·4) | 220,186(164,160 to 287,607) | 598,045(429,743 to 836,398) | 3·3(2·7 to 3·9) |
| High systolic blood pressure | 15,693,134(12,208,391 to 19,314,501) | 31,938,900(25,193,679 to 39,140,725) | 2·5(2·3 to 2·7) | 614,619(483,715 to 756,067) | 1,441,190(1,121,365 to 1,775,433) | 3·0(2·8 to 3·2) |
| High body-mass index | 4,074,496(946,556 to 9,142,298) | 14,252,690(6,508,595 to 23,751,935) | 4·4(4·2 to 4·6) | 118,292(26,361 to 271,542) | 431,628(186,050 to 746,597) | 4·5(4·3 to 4·8) |
| Low bone mineral density | 730,294(579,862 to 985,146) | 1,698,706(1,281,581 to 2,076,364) | 3·0(2·8 to 3·1) | 17,331(13,173 to 25,117) | 44,732(29,945 to 57,911) | 3·4(3·2 to 3·5) |
| Kidney dysfunction | 3,914,397(3,276,428 to 4,590,571) | 7,204,969(5,765,700 to 9,013,885) | 2·1(1·9 to 2·4) | 123,009(102,146 to 147,086) | 298,534(235,670 to 376,339) | 3·1(2·9 to 3·4) |
| High LDL cholesterol | 5,132,690(3,963,778 to 6,488,116) | 12,018,428(8,845,432 to 15,513,958) | 3·0(2·8 to 3·1) | 171,701(129,705 to 225,140) | 505,471(352,899 to 679,518) | 3·8(3·6 to 4·0) |
| **Female** |  |  |  |  |  |  |
| High fasting plasma glucose | 6,574,200(5,167,627 to 8,251,919) | 12,148,703(9,335,104 to 15,382,986) | 2·1(1·6 to 2·5) | 230,624(173,217 to 306,268) | 469,510(329,121 to 643,184) | 2·4(1·8 to 3·0) |
| High systolic blood pressure | 13,679,271(10,722,791 to 16,879,893) | 22,502,716(17,465,018 to 27,797,743) | 1·7(1·5 to 2·0) | 607,576(481,097 to 754,445) | 1,158,689(875,875 to 1,457,131) | 2·2(1·9 to 2·6) |
| High body-mass index | 3,801,852(1,039,204 to 8,000,206) | 10,577,351(4,981,691 to 17,720,265) | 3·6(3·5 to 3·7) | 116,706(30,739 to 253,682) | 333,070(146,158 to 590,392) | 3·7(3·4 to 3·9) |
| Low bone mineral density | 609,240(503,316 to 721,468) | 1,621,569(1,266,285 to 2,016,399) | 3·4(3·3 to 3·6) | 14,670(11,974 to 17,982) | 45,125(26,172 to 58,230) | 4·0(3·8 to 4·2) |
| Kidney dysfunction | 3,764,452(3,145,206 to 4,428,545) | 6,148,940(4,996,899 to 7,535,759) | 1·8(1·4 to 2·1) | 125,335(102,404 to 151,558) | 275,003(210,353 to 347,909) | 2·9(2·5 to 3·2) |
| High LDL cholesterol | 3,756,409(2,935,929 to 4,870,787) | 7,795,534(5,671,801 to 10,492,477) | 2·6(2·5 to 2·7) | 145,359(106,643 to 200,392) | 410,512(273,785 to 590,077) | 3·6(3·4 to 3·9) |
| **Age standardized rate per 100,000 population** | | | | | | |
| **Male** | | | | | | |
| High fasting plasma glucose | 1,703(1,323 to 2,141) | 1,762(1,337 to 2,296) | 0·0(-0·5 to 0·5) | 75(55 to 101) | 80(56 to 115) | 0·0(-0·6 to 0·7) |
| High systolic blood pressure | 4,054(3,258 to 4,914) | 3,570(2,838 to 4,371) | -0·4(-0·6 to -0·2) | 213(171 to 259) | 197(155 to 240) | -0·3(-0·5 to 0·0) |
| High body-mass index | 869(197 to 1,992) | 1,453(664 to 2,426) | 1·8(1·6 to 1·9) | 31(7 to 73) | 49(21 to 87) | 1·6(1·4 to 1·8) |
| Low bone mineral density | 178(142 to 231) | 190(145 to 232) | 0·2(0·1 to 0·4) | 6(4 to 8) | 7(4 to 9) | 0·5(0·3 to 0·7) |
| Kidney dysfunction | 908(762 to 1,066) | 817(662 to 1,010) | -0·3(-0·6 to -0·1) | 40(33 to 48) | 41(33 to 52) | 0·2(-0·1 to 0·4) |
| High LDL cholesterol | 1,212(931 to 1,584) | 1,352(989 to 1,754) | 0·4(0·2 to 0·5) | 57(40 to 79) | 72(48 to 100) | 0·8(0·6 to 1·0) |
| **Female** | | | | | | |
| High fasting plasma glucose | 1,531(1,204 to 1,936) | 1,191(916 to 1,509) | -0·9(-1·2 to -0·7) | 63(47 to 86) | 49(34 to 67) | -1·0(-1·3 to -0·7) |
| High systolic blood pressure | 3,337(2,636 to 4,083) | 2,225(1,728 to 2,743) | -1·4(-1·7 to -1·1) | 176(138 to 217) | 123(93 to 155) | -1·3(-1·6 to -1·0) |
| High body-mass index | 833(227 to 1,760) | 1,014(473 to 1,705) | 0·7(0·6 to 0·8) | 29(7 to 63) | 33(14 to 60) | 0·5(0·3 to 0·7) |
| Low bone mineral density | 154(127 to 182) | 163(126 to 202) | 0·2(0·1 to 0·3) | 5(4 to 6) | 5(3 to 7) | 0·3(0·1 to 0·5) |
| Kidney dysfunction | 833(696 to 988) | 621(504 to 759) | -0·9(-1·3 to -0·6) | 34(27 to 41) | 29(22 to 37) | -0·4(-0·8 to 0·0) |
| High LDL cholesterol | 889(678 to 1,191) | 786(570 to 1,067) | -0·4(-0·6 to -0·3) | 43(30 to 62) | 45(29 to 64) | 0·1(0·0 to 0·3) |

AAPC, average annual percent changes; DALYs, disability-adjusted life years; UI, uncertainty interval; CI, confidence interval.

| **sTable 2**. Trend in number and age-standardized rate of DALYs and deaths for level 3 causes attributable to six metabolic risk factors in China, 1990-2019 | | | | | | |
| --- | --- | --- | --- | --- | --- | --- |
|  | Number | | | Age standardized rate per 100,000 population | | |
|  | 1990 | 2019 | AAPC (95% CI) | 1990 | 2019 | AAPC (95% CI) |
| **DALYs** |  |  |  |  |  |  |
| **High fasting plasma glucose** |  |  |  |  |  |  |
| Tuberculosis | 389,268(234,926 to 568,709) | 125,538(73,808 to 183,637) | -3·9(-4·3 to -3·5) | 42(25 to 62) | 6(4 to 9) | -6·4(-6·8 to -6·1) |
| Liver cancer | 30,074(6,595 to 69,010) | 29,721(6,937 to 66,181) | -0·1(-0·5 to 0·3) | 3(1 to 8) | 1(0 to 3) | -2·9(-3·3 to -2·6) |
| Tracheal, bronchus, and lung cancer | 345,569(72,605 to 813,014) | 1,107,654(242,107 to 2,548,449) | 4·1(3·8 to 4·3) | 40(9 to 94) | 54(12 to 123) | 1·0(0·8 to 1·2) |
| Breast cancer | 55,063(10,119 to 128,018) | 146,739(26,655 to 348,416) | 3·4(3·1 to 3·7) | 6(1 to 14) | 7(1 to 17) | 0·4(0·1 to 0·7) |
| Colon and rectum cancer | 104,239(24,054 to 240,404) | 395,340(85,260 to 915,610) | 4·7(4·4 to 5·0) | 12(3 to 29) | 20(4 to 45) | 1·5(1·2 to 1·8) |
| Pancreatic cancer | 36,617(7,793 to 85,001) | 175,495(38,289 to 404,020) | 5·5(5·2 to 5·8) | 4(1 to 10) | 8(2 to 19) | 2·4(2·1 to 2·7) |
| Ovarian cancer | 10,399(1,883 to 27,641) | 44,615(7,595 to 113,363) | 5·0(4·6 to 5·4) | 1(0 to 3) | 2(0 to 5) | 2·0(1·6 to 2·4) |
| Bladder cancer | 21,474(4,273 to 49,136) | 55,650(10,510 to 124,278) | 3·2(2·7 to 3·8) | 3(1 to 7) | 3(1 to 6) | -0·1(-0·6 to 0·4) |
| Ischemic heart disease | 2,072,260(1,277,153 to 3,222,225) | 6,014,932(3,774,590 to 9,306,629) | 3·6(3·0 to 4·3) | 277(171 to 428) | 327(201 to 508) | 0·5(-0·1 to 1·1) |
| Stroke | 4,570,623(3,148,224 to 6,619,910) | 7,533,231(5,073,192 to 11,085,966) | 1·6(1·0 to 2·2) | 577(387 to 835) | 386(256 to 577) | -1·5(-2·1 to -0·9) |
| Peripheral artery disease | 14,589(7,937 to 24,584) | 37,686(21,305 to 62,767) | 3·2(2·9 to 3·6) | 2(1 to 3) | 2(1 to 3) | -0·2(-0·5 to 0·2) |
| Alzheimer's disease and other dementias | 124,438(18,782 to 445,419) | 442,695(73,488 to 1,425,147) | 4·4(4·2 to 4·6) | 24(4 to 85) | 27(4 to 89) | 0·4(0·2 to 0·6) |
| Diabetes mellitus | 4,260,040(3,450,976 to 5,252,928) | 9,900,139(7,861,133 to 12,244,669) | 2·9(2·6 to 3·1) | 465(379 to 571) | 495(393 to 611) | 0·1(-0·1 to 0·4) |
| Chronic kidney disease | 1,357,347(1,122,111 to 1,601,070) | 2,134,091(1,763,499 to 2,538,659) | 1·6(1·2 to 2·0) | 142(119 to 167) | 109(90 to 128) | -0·8(-1·2 to -0·4) |
| Blindness and vision loss | 31,679(7,189 to 76,325) | 84,914(18,868 to 200,923) | 3·5(3·3 to 3·7) | 5(1 to 12) | 5(1 to 11) | -0·2(-0·4 to 0·1) |
| **High systolic blood pressure** |  |  |  |  |  |  |
| Rheumatic heart disease | 742,250(462,441 to 1,155,465) | 450,558(294,885 to 694,341) | -1·7(-2·0 to -1·5) | 83(52 to 137) | 23(15 to 36) | -4·3(-4·5 to -4·1) |
| Ischemic heart disease | 6,332,425(4,950,620 to 7,809,007) | 18,552,713(14,741,741 to 22,628,942) | 3·8(3·5 to 4·0) | 797(628 to 988) | 986(776 to 1,210) | 0·7(0·5 to 1·0) |
| Stroke | 15,189,829(11,658,878 to 18,941,469) | 25,176,256(20,271,928 to 30,148,436) | 1·8(1·6 to 2·0) | 1,855(1,436 to 2,296) | 1,280(1,028 to 1,531) | -1·3(-1·4 to -1·1) |
| Hypertensive heart disease | 5,042,174(3,661,268 to 5,792,671) | 5,594,910(3,877,275 to 6,532,987) | 0·4(0·1 to 0·7) | 693(526 to 786) | 313(214 to 364) | -2·7(-3·0 to -2·4) |
| Cardiomyopathy and myocarditis | 40,000(23,271 to 72,690) | 96,399(65,355 to 132,169) | 3·1(2·8 to 3·3) | 4(3 to 8) | 5(3 to 7) | 0·5(0·2 to 0·7) |
| Atrial fibrillation and flutter | 217,091(150,778 to 300,060) | 700,754(494,049 to 953,254) | 4·1(4·0 to 4·2) | 32(22 to 44) | 37(27 to 51) | 0·6(0·5 to 0·7) |
| Aortic aneurysm | 52,450(35,374 to 75,857) | 141,700(108,068 to 182,227) | 3·5(3·3 to 3·6) | 6(4 to 9) | 7(5 to 9) | 0·6(0·4 to 0·7) |
| Peripheral artery disease | 15,956(8,181 to 28,066) | 47,540(25,117 to 84,157) | 3·8(3·8 to 3·9) | 2(1 to 4) | 2(1 to 4) | 0·3(0·3 to 0·4) |
| Endocarditis | 22,296(13,987 to 31,119) | 38,546(27,170 to 52,764) | 1·9(1·7 to 2·2) | 2(2 to 3) | 2(1 to 3) | -0·6(-1·0 to -0·3) |
| Non-rheumatic valvular heart disease | 12,614(7,225 to 19,072) | 30,921(22,429 to 41,095) | 3·1(2·9 to 3·3) | 1(1 to 2) | 2(1 to 2) | 0·4(0·2 to 0·7) |
| Chronic kidney disease | 1,579,368(1,317,964 to 1,870,237) | 3,372,435(2,783,861 to 4,004,996) | 2·7(2·5 to 3·0) | 182(153 to 211) | 174(144 to 206) | -0·1(-0·3 to 0·2) |
| Other cardiovascular and circulatory diseases | 125,952(94,847 to 167,652) | 238,883(185,268 to 297,014) | 2·2(2·1 to 2·4) | 15(11 to 20) | 12(10 to 15) | -0·6(-0·8 to -0·4) |
| **High body-mass index** |  |  |  |  |  |  |
| Esophageal cancer | 306,709(49,525 to 827,903) | 859,654(221,497 to 1,875,382) | 3·6(3·4 to 3·8) | 34(6 to 93) | 41(11 to 89) | 0·6(0·4 to 0·8) |
| Liver cancer | 339,788(63,119 to 881,904) | 570,373(183,232 to 1,205,391) | 1·8(1·3 to 2·3) | 34(6 to 89) | 28(9 to 59) | -0·8(-1·2 to -0·3) |
| Breast cancer | 62,298(11,394 to 150,487) | 336,583(93,779 to 680,214) | 5·9(5·5 to 6·3) | 7(1 to 17) | 15(4 to 31) | 2·8(2·6 to 3·1) |
| Uterine cancer | 38,378(9,764 to 83,099) | 90,829(40,885 to 166,472) | 3·0(2·8 to 3·3) | 4(1 to 9) | 4(2 to 8) | 0·3(0·1 to 0·4) |
| Colon and rectum cancer | 53,125(11,759 to 127,189) | 376,133(149,260 to 689,708) | 7·0(6·8 to 7·1) | 6(1 to 14) | 18(7 to 34) | 4·1(4·0 to 4·3) |
| Gallbladder and biliary tract cancer | 16,054(3,730 to 40,845) | 81,473(31,251 to 157,725) | 5·8(5·5 to 6·0) | 2(0 to 5) | 4(2 to 8) | 2·7(2·5 to 2·9) |
| Pancreatic cancer | 12,709(2,179 to 34,463) | 106,354(27,250 to 240,617) | 7·6(7·4 to 7·7) | 1(0 to 4) | 5(1 to 12) | 4·6(4·4 to 4·8) |
| Ovarian cancer | 2,352(-53 to 7,379) | 16,701(-398 to 43,257) | 7·0(6·9 to 7·1) | 0(0 to 1) | 1(0 to 2) | 4·2(4·1 to 4·3) |
| **Kidney cancer** | 8,117(1,906 to 18,424) | 70,544(29,312 to 127,229) | 7·7(7·6 to 7·9) | 1(0 to 2) | 3(1 to 6) | 4·9(4·7 to 5·0) |
| Thyroid cancer | 3,227(682 to 7,943) | 14,811(4,839 to 30,476) | 5·3(5·1 to 5·6) | 0(0 to 1) | 1(0 to 2) | 2·7(2·5 to 2·8) |
| Non-Hodgkin lymphoma | 7,344(1,462 to 19,495) | 47,354(16,139 to 99,617) | 6·7(6·3 to 7·0) | 1(0 to 2) | 2(1 to 5) | 4·1(3·7 to 4·4) |
| Multiple myeloma | 2,828(524 to 7,430) | 14,687(4,528 to 30,414) | 5·8(5·6 to 6·0) | 0(0 to 1) | 1(0 to 1) | 3·0(2·8 to 3·2) |
| Leukemia | 32,519(6,124 to 82,757) | 84,303(30,802 to 167,814) | 3·3(3·1 to 3·6) | 3(1 to 7) | 4(2 to 9) | 1·4(1·2 to 1·7) |
| Ischemic heart disease | 1,183,844(283,868 to 2,643,152) | 5,073,254(2,233,964 to 8,744,122) | 5·2(5·0 to 5·4) | 128(30 to 286) | 258(112 to 447) | 2·5(2·3 to 2·6) |
| Stroke | 3,376,374(849,027 to 7,253,492) | 8,188,302(3,891,288 to 13,434,158) | 3·1(2·9 to 3·3) | 352(87 to 762) | 398(189 to 654) | 0·4(0·2 to 0·7) |
| Hypertensive heart disease | 727,467(176,938 to 1,575,574) | 1,514,532(673,366 to 2,626,321) | 2·5(2·0 to 3·0) | 92(21 to 208) | 80(34 to 145) | -0·5(-1·0 to 0·0) |
| Atrial fibrillation and flutter | 40,270(8,841 to 97,700) | 228,050(88,027 to 445,218) | 6·2(6·1 to 6·2) | 6(1 to 15) | 12(5 to 24) | 2·5(2·4 to 2·6) |
| Asthma | 98,457(25,467 to 228,184) | 175,325(75,052 to 320,742) | 2·1(1·9 to 2·2) | 11(3 to 26) | 10(5 to 19) | -0·1(-0·3 to 0·2) |
| Gallbladder and biliary diseases | 139,950(32,648 to 342,137) | 384,258(158,489 to 746,027) | 3·5(3·4 to 3·6) | 15(4 to 37) | 19(8 to 37) | 0·8(0·7 to 0·9) |
| Alzheimer's disease and other dementias | 72,969(8,730 to 252,707) | 477,209(101,059 to 1,388,766) | 6·7(6·6 to 6·8) | 13(2 to 46) | 28(6 to 82) | 2·7(2·6 to 2·8) |
| Diabetes mellitus | 771,802(209,708 to 1,609,362) | 3,737,576(1,913,966 to 5,903,926) | 5·6(5·5 to 5·7) | 80(21 to 167) | 182(94 to 286) | 2·8(2·7 to 3·0) |
| Chronic kidney disease | 297,282(75,328 to 668,658) | 1,200,356(542,875 to 2,014,596) | 5·0(4·7 to 5·3) | 33(8 to 74) | 58(26 to 99) | 2·1(1·9 to 2·4) |
| Osteoarthritis | 99,197(16,851 to 280,352) | 557,902(165,070 to 1,343,750) | 6·1(5·9 to 6·3) | 11(2 to 31) | 26(8 to 63) | 3·0(2·8 to 3·2) |
| Low back pain | 155,486(33,389 to 376,821) | 460,685(184,490 to 899,388) | 3·8(3·7 to 3·9) | 15(3 to 37) | 23(9 to 46) | 1·4(1·3 to 1·6) |
| Gout | 18,680(4,003 to 47,523) | 115,104(44,197 to 231,850) | 6·5(6·3 to 6·7) | 2(0 to 5) | 6(2 to 11) | 3·8(3·6 to 4·0) |
| Blindness and vision loss | 9,122(1,861 to 23,630) | 47,688(15,902 to 101,631) | 5·9(5·7 to 6·2) | 1(0 to 3) | 3(1 to 5) | 2·4(2·2 to 2·5) |
| **Low bone mineral density** |  |  |  |  |  |  |
| Road injuries | 537,569(432,389 to 724,960) | 1,195,859(947,985 to 1,415,383) | 2·8(2·6 to 3·0) | 58(47 to 78) | 58(47 to 69) | 0·0(-0·1 to 0·1) |
| Other transport injuries | 80,275(64,852 to 103,780) | 78,651(63,197 to 94,695) | -0·1(-0·6 to 0·3) | 9(7 to 11) | 4(3 to 5) | -2·9(-3·3 to -2·5) |
| Falls | 620,852(503,823 to 753,230) | 1,839,375(1,346,044 to 2,316,329) | 3·8(3·7 to 4·0) | 90(74 to 109) | 105(76 to 131) | 0·5(0·4 to 0·7) |
| Exposure to mechanical forces | 63,174(47,142 to 92,266) | 164,611(116,916 to 210,564) | 3·4(2·9 to 3·9) | 7(5 to 10) | 8(6 to 10) | 0·5(0·0 to 0·9) |
| Animal contact | 15,552(11,217 to 19,921) | 11,695(8,385 to 17,040) | -1·0(-1·1 to -0·8) | 2(1 to 2) | 1(0 to 1) | -3·9(-4·0 to -3·7) |
| Interpersonal violence | 22,113(17,657 to 27,191) | 30,084(22,865 to 38,560) | 1·1(0·9 to 1·2) | 3(2 to 3) | 1(1 to 2) | -1·9(-2·0 to -1·8) |
| **Kidney dysfunction** |  |  |  |  |  |  |
| Ischemic heart disease | 1,405,191(931,472 to 1,946,778) | 3,776,976(2,431,295 to 5,253,210) | 3·5(3·0 to 4·0) | 182(117 to 254) | 209(134 to 291) | 0·5(0·0 to 1·0) |
| Stroke | 2,253,520(1,811,414 to 2,794,712) | 3,672,749(2,903,821 to 4,452,126) | 1·7(1·6 to 1·9) | 268(214 to 336) | 184(144 to 224) | -1·2(-1·4 to -1·0) |
| Peripheral artery disease | 10,458(5,983 to 17,378) | 25,816(14,645 to 44,192) | 3·2(3·0 to 3·4) | 1(1 to 2) | 1(1 to 2) | 0·0(0·0 to 0·1) |
| Chronic kidney disease | 3,997,026(3,531,282 to 4,458,760) | 5,831,843(4,992,206 to 6,645,333) | 1·4(1·0 to 1·7) | 410(364 to 457) | 312(268 to 354) | -0·9(-1·2 to -0·5) |
| Gout | 12,654(7,655 to 18,640) | 46,523(28,108 to 68,591) | 4·6(4·5 to 4·8) | 2(1 to 2) | 2(1 to 4) | 1·3(1·1 to 1·5) |
| **High LDL cholesterol** |  |  |  |  |  |  |
| Ischemic heart disease | 6,941,572(5,615,925 to 8,542,968) | 15,386,309(11,920,599 to 19,336,537) | 2·8(2·6 to 2·9) | 807(637 to 1,003) | 819(629 to 1,041) | 0·1(-0·1 to 0·3) |
| Stroke | 1,947,527(1,229,161 to 3,174,413) | 4,427,653(2,418,020 to 7,710,339) | 2·9(2·7 to 3·1) | 239(136 to 416) | 233(120 to 416) | -0·1(-0·3 to 0·1) |
| **Deaths** |  |  |  |  |  |  |
| **High fasting plasma glucose** |  |  |  |  |  |  |
| Tuberculosis | 12,665(7,319 to 19,035) | 3,646(2,056 to 5,638) | -4·3(-4·7 to -3·8) | 2(1 to 2) | 0(0 to 0) | -7·0(-7·4 to -6·6) |
| Liver cancer | 1,137(252 to 2,566) | 1,262(301 to 2,797) | 0·3(-0·1 to 0·7) | 0(0 to 0) | 0(0 to 0) | -2·7(-3·1 to -2·4) |
| Tracheal, bronchus, and lung cancer | 14,427(3,113 to 33,824) | 53,005(11,785 to 121,265) | 4·6(4·3 to 4·8) | 2(0 to 4) | 3(1 to 6) | 1·3(1·1 to 1·6) |
| Breast cancer | 1,931(362 to 4,510) | 5,453(1,003 to 12,882) | 3·6(3·3 to 3·9) | 0(0 to 1) | 0(0 to 1) | 0·4(0·1 to 0·8) |
| Colon and rectum cancer | 4,423(1,025 to 10,166) | 18,161(4,030 to 41,451) | 5·0(4·6 to 5·3) | 1(0 to 1) | 1(0 to 2) | 1·5(1·2 to 1·9) |
| Pancreatic cancer | 1,523(323 to 3,532) | 8,046(1,759 to 18,320) | 5·9(5·6 to 6·2) | 0(0 to 0) | 0(0 to 1) | 2·5(2·2 to 2·9) |
| Ovarian cancer | 385(70 to 1,024) | 1,758(299 to 4,429) | 5·2(4·9 to 5·6) | 0(0 to 0) | 0(0 to 0) | 2·0(1·6 to 2·4) |
| Bladder cancer | 1,061(213 to 2,421) | 2,970(565 to 6,595) | 3·5(3·0 to 4·0) | 0(0 to 0) | 0(0 to 0) | -0·1(-0·6 to 0·4) |
| Ischemic heart disease | 95,190(58,410 to 149,663) | 341,686(193,747 to 557,999) | 4·4(3·8 to 5·0) | 16(9 to 26) | 21(11 to 35) | 0·7(0·2 to 1·2) |
| Stroke | 202,738(133,708 to 298,337) | 358,181(227,737 to 561,374) | 1·8(1·1 to 2·5) | 31(19 to 47) | 20(12 to 33) | -1·6(-2·2 to -0·9) |
| Peripheral artery disease | 123(94 to 175) | 474(372 to 606) | 4·7(4·0 to 5·3) | 0(0 to 0) | 0(0 to 0) | 0·9(0·4 to 1·4) |
| Alzheimer's disease and other dementias | 6,521(629 to 26,296) | 23,988(2,536 to 94,088) | 4·6(4·3 to 4·8) | 2(0 to 7) | 2(0 to 7) | 0·1(-0·1 to 0·3) |
| Diabetes mellitus | 70,089(61,639 to 79,978) | 172,892(147,237 to 198,952) | 3·2(3·1 to 3·4) | 9(8 to 10) | 9(8 to 11) | 0·1(-0·1 to 0·3) |
| Chronic kidney disease | 38,596(31,794 to 46,116) | 76,033(60,573 to 91,454) | 2·4(2·2 to 2·6) | 5(4 to 6) | 4(3 to 5) | -0·5(-0·7 to -0·4) |
| **High systolic blood pressure** |  |  |  |  |  |  |
| Rheumatic heart disease | 27,138(16,393 to 46,065) | 18,226(11,270 to 29,323) | -1·4(-1·6 to -1·2) | 4(2 to 7) | 1(1 to 2) | -4·4(-4·7 to -4·1) |
| Ischemic heart disease | 265,786(206,604 to 329,317) | 969,221(741,700 to 1,217,123) | 4·5(4·3 to 4·8) | 43(32 to 55) | 59(43 to 75) | 1·1(0·8 to 1·3) |
| Stroke | 624,653(484,209 to 784,337) | 1,126,039(889,924 to 1,370,838) | 2·0(1·8 to 2·3) | 93(70 to 117) | 63(49 to 78) | -1·3(-1·5 to -1·1) |
| Hypertensive heart disease | 241,298(180,926 to 275,423) | 320,090(201,652 to 373,927) | 1·0(0·6 to 1·4) | 42(34 to 48) | 21(13 to 24) | -2·4(-2·8 to -2·0) |
| Cardiomyopathy and myocarditis | 1,327(764 to 2,484) | 3,786(2,520 to 5,284) | 3·7(3·4 to 3·9) | 0(0 to 0) | 0(0 to 0) | 0·7(0·5 to 0·9) |
| Atrial fibrillation and flutter | 5,013(3,642 to 6,634) | 17,513(13,026 to 23,149) | 4·4(4·1 to 4·6) | 1(1 to 2) | 1(1 to 2) | 0·2(0·1 to 0·4) |
| Aortic aneurysm | 2,075(1,408 to 2,971) | 6,083(4,584 to 7,789) | 3·7(3·6 to 3·9) | 0(0 to 0) | 0(0 to 0) | 0·5(0·3 to 0·6) |
| Peripheral artery disease | 132(86 to 197) | 570(395 to 797) | 5·2(5·0 to 5·4) | 0(0 to 0) | 0(0 to 0) | 1·3(1·0 to 1·5) |
| Endocarditis | 734(472 to 983) | 1,461(1,023 to 1,998) | 2·4(2·1 to 2·7) | 0(0 to 0) | 0(0 to 0) | -0·7(-1·0 to -0·4) |
| Non-rheumatic valvular heart disease | 433(248 to 653) | 1,057(755 to 1,418) | 3·1(2·8 to 3·3) | 0(0 to 0) | 0(0 to 0) | 0·1(-0·2 to 0·3) |
| Chronic kidney disease | 49,315(41,426 to 58,188) | 127,960(105,241 to 151,102) | 3·3(3·2 to 3·5) | 7(6 to 8) | 7(6 to 9) | 0·1(-0·1 to 0·2) |
| Other cardiovascular and circulatory diseases | 4,290(3,241 to 6,106) | 7,875(6,154 to 9,818) | 2·1(1·8 to 2·3) | 1(0 to 1) | 0(0 to 1) | -1·2(-1·4 to -0·9) |
| **High body-mass index** |  |  |  |  |  |  |
| Esophageal cancer | 11,783(1,922 to 32,264) | 36,181(9,426 to 79,606) | 3·9(3·8 to 4·1) | 1(0 to 4) | 2(0 to 4) | 0·8(0·5 to 1·0) |
| Liver cancer | 10,362(1,929 to 26,876) | 18,965(6,165 to 40,278) | 2·1(1·8 to 2·5) | 1(0 to 3) | 1(0 to 2) | -0·7(-1·0 to -0·4) |
| Breast cancer | 2,191(392 to 5,325) | 12,059(3,393 to 25,190) | 6·1(5·7 to 6·4) | 0(0 to 1) | 1(0 to 1) | 2·8(2·4 to 3·2) |
| Uterine cancer | 1,222(316 to 2,618) | 2,967(1,306 to 5,508) | 3·1(2·9 to 3·3) | 0(0 to 0) | 0(0 to 0) | 0·1(-0·1 to 0·3) |
| Colon and rectum cancer | 1,818(400 to 4,351) | 14,147(5,480 to 26,648) | 7·3(7·1 to 7·5) | 0(0 to 1) | 1(0 to 1) | 4·1(3·9 to 4·3) |
| Gallbladder and biliary tract cancer | 625(143 to 1,593) | 3,518(1,330 to 6,901) | 6·1(5·9 to 6·4) | 0(0 to 0) | 0(0 to 0) | 2·8(2·6 to 3·0) |
| Pancreatic cancer | 456(78 to 1,231) | 4,236(1,108 to 9,602) | 8·0(7·8 to 8·2) | 0(0 to 0) | 0(0 to 0) | 4·7(4·5 to 4·9) |
| Ovarian cancer | 71(-2 to 222) | 571(-13 to 1,496) | 7·4(7·3 to 7·6) | 0(0 to 0) | 0(0 to 0) | 4·3(4·2 to 4·5) |
| **Kidney cancer** | 276(65 to 627) | 2,589(1,082 to 4,699) | 8·0(7·8 to 8·2) | 0(0 to 0) | 0(0 to 0) | 4·8(4·6 to 5·0) |
| Thyroid cancer | 107(22 to 264) | 536(174 to 1,120) | 5·7(5·5 to 6·0) | 0(0 to 0) | 0(0 to 0) | 2·6(2·4 to 2·8) |
| Non-Hodgkin lymphoma | 226(45 to 599) | 1,593(528 to 3,315) | 7·0(6·6 to 7·3) | 0(0 to 0) | 0(0 to 0) | 4·0(3·8 to 4·2) |
| Multiple myeloma | 101(18 to 262) | 544(169 to 1,135) | 6·0(5·8 to 6·2) | 0(0 to 0) | 0(0 to 0) | 2·9(2·7 to 3·0) |
| Leukemia | 831(160 to 2,104) | 2,535(920 to 5,039) | 3·9(3·6 to 4·2) | 0(0 to 0) | 0(0 to 0) | 1·5(1·2 to 1·8) |
| Ischemic heart disease | 39,406(9,214 to 89,039) | 203,609(84,285 to 367,441) | 5·8(5·6 to 6·0) | 5(1 to 12) | 11(5 to 21) | 2·7(2·5 to 2·9) |
| Stroke | 106,664(25,826 to 233,294) | 266,755(119,931 to 453,820) | 3·2(3·0 to 3·5) | 12(3 to 27) | 13(6 to 23) | 0·3(0·0 to 0·6) |
| Hypertensive heart disease | 31,035(7,085 to 70,851) | 73,300(28,352 to 142,308) | 2·9(2·4 to 3·5) | 5(1 to 12) | 4(2 to 9) | -0·4(-0·9 to 0·0) |
| Atrial fibrillation and flutter | 976(209 to 2,389) | 5,876(2,152 to 11,857) | 6·4(6·1 to 6·6) | 0(0 to 1) | 0(0 to 1) | 2·1(1·9 to 2·3) |
| Asthma | 2,514(549 to 6,524) | 3,270(1,235 to 6,227) | 0·9(0·5 to 1·3) | 0(0 to 1) | 0(0 to 0) | -2·4(-2·7 to -2·2) |
| Gallbladder and biliary diseases | 1,846(428 to 4,213) | 3,053(1,235 to 5,627) | 1·7(1·3 to 2·1) | 0(0 to 1) | 0(0 to 0) | -1·4(-1·8 to -1·1) |
| Alzheimer's disease and other dementias | 3,531(290 to 13,782) | 23,787(3,264 to 78,659) | 6·8(6·7 to 6·9) | 1(0 to 3) | 2(0 to 6) | 2·3(2·2 to 2·5) |
| Diabetes mellitus | 10,510(2,938 to 21,514) | 47,530(22,514 to 76,632) | 5·3(5·2 to 5·5) | 1(0 to 3) | 2(1 to 4) | 2·3(2·1 to 2·5) |
| Chronic kidney disease | 8,450(2,079 to 18,788) | 37,077(15,764 to 65,372) | 5·2(5·0 to 5·4) | 1(0 to 3) | 2(1 to 3) | 2·0(1·8 to 2·3) |
| **Low bone mineral density** |  |  |  |  |  |  |
| Road injuries | 13,317(10,610 to 18,889) | 27,523(21,566 to 33,066) | 2·5(2·4 to 2·7) | 2(1 to 2) | 1(1 to 2) | -0·3(-0·4 to -0·2) |
| Other transport injuries | 1,934(1,550 to 2,649) | 1,709(1,356 to 2,073) | -0·5(-0·9 to 0·0) | 0(0 to 0) | 0(0 to 0) | -3·3(-3·8 to -2·9) |
| Falls | 14,512(11,965 to 18,810) | 56,639(30,514 to 71,875) | 4·8(4·4 to 5·2) | 3(3 to 4) | 4(2 to 5) | 0·9(0·6 to 1·1) |
| Exposure to mechanical forces | 1,446(1,043 to 2,338) | 3,436(2,092 to 4,376) | 3·1(2·7 to 3·5) | 0(0 to 0) | 0(0 to 0) | 0·1(-0·4 to 0·6) |
| Animal contact | 344(221 to 412) | 184(145 to 238) | -2·1(-2·4 to -1·9) | 0(0 to 0) | 0(0 to 0) | -5·0(-5·2 to -4·8) |
| Interpersonal violence | 449(367 to 527) | 366(296 to 447) | -0·7(-0·9 to -0·5) | 0(0 to 0) | 0(0 to 0) | -3·7(-3·9 to -3·6) |
| **Kidney dysfunction** |  |  |  |  |  |  |
| Ischemic heart disease | 61,221(39,573 to 85,649) | 218,411(137,830 to 306,684) | 4·5(4·0 to 5·0) | 10(6 to 15) | 14(9 to 20) | 1·0(0·6 to 1·5) |
| Stroke | 88,430(70,294 to 112,446) | 158,128(118,597 to 196,772) | 2·1(1·8 to 2·5) | 12(10 to 16) | 9(6 to 11) | -1·1(-1·6 to -0·7) |
| Peripheral artery disease | 86(60 to 125) | 272(188 to 374) | 4·2(3·9 to 4·4) | 0(0 to 0) | 0(0 to 0) | 0·9(0·7 to 1·1) |
| Chronic kidney disease | 98,607(86,800 to 111,078) | 196,726(168,241 to 224,684) | 2·4(2·3 to 2·5) | 13(12 to 15) | 11(10 to 13) | -0·5(-0·6 to -0·3) |
| **High LDL cholesterol** |  |  |  |  |  |  |
| Ischemic heart disease | 249,254(194,105 to 314,056) | 733,295(520,883 to 982,246) | 3·8(3·5 to 4·0) | 38(27 to 51) | 45(30 to 62) | 0·6(0·4 to 0·8) |
| Stroke | 67,806(33,489 to 130,613) | 182,689(71,552 to 383,129) | 3·5(3·2 to 3·7) | 11(4 to 23) | 11(4 to 24) | 0·0(-0·3 to 0·3) |

AAPC, average annual percent changes; DALYs, disability-adjusted life years; UI, uncertainty interval; CI, confidence interval.

**sTable 3**. Trend in number and age-standardized rate of DALYs and deaths for level 4 causes attributable to six metabolic risk factors in China, 1990-2019

|  | Number | | | Age standardized rate per 100,000 population | | |
| --- | --- | --- | --- | --- | --- | --- |
|  | 1990 | 2019 | AAPC (95% CI) | 1990 | 2019 | AAPC (95% CI) |
| **DALYs** |  |  |  |  |  |  |
| **High fasting plasma glucose** |  |  |  |  |  |  |
| Ischemic stroke | 1,352,615(724,692 to 2,398,297) | 3,667,314(1,936,682 to 6,510,542) | 3·4(2·8 to 4·0) | 183(95 to 341) | 192(100 to 354) | 0·1(-0·5 to 0·6) |
| Intracerebral hemorrhage | 2,504,782(1,535,000 to 3,700,369) | 3,506,649(2,231,722 to 5,211,237) | 1·0(0·5 to 1·5) | 308(191 to 455) | 176(111 to 266) | -2·1(-2·6 to -1·7) |
| Subarachnoid hemorrhage | 713,226(384,912 to 1,112,571) | 359,268(220,875 to 533,972) | -2·5(-3·0 to -1·9) | 86(46 to 135) | 18(11 to 27) | -5·4(-5·9 to -4·9) |
| Glaucoma | 4,432(1,018 to 10,381) | 8,225(1,874 to 19,370) | 2·2(1·8 to 2·5) | 1(0 to 2) | 0(0 to 1) | -1·5(-1·7 to -1·2) |
| Cataract | 27,247(5,656 to 66,204) | 76,689(15,737 to 182,771) | 3·7(3·5 to 3·9) | 4(1 to 10) | 4(1 to 10) | 0·0(-0·2 to 0·2) |
| Drug-susceptible tuberculosis | 367,193(221,840 to 545,717) | 115,440(67,663 to 171,861) | -4·1(-4·5 to -3·6) | 40(24 to 58) | 6(3 to 9) | -6·5(-6·9 to -6·1) |
| Multidrug-resistant tuberculosis without extensive drug resistance | 22,075(4,543 to 64,377) | 8,589(1,406 to 26,962) | -3·4(-4·1 to -2·7) | 2(0 to 7) | 0(0 to 1) | -5·9(-6·6 to -5·3) |
| Extensively drug-resistant tuberculosis | 0(0 to 0) | 1,509(232 to 4,684) | - | 0(0 to 0) | 0(0 to 0) | - |
| Latent tuberculosis infection | 0(0 to 0) | 0(0 to 0) | - | 0(0 to 0) | 0(0 to 0) | - |
| Diabetes mellitus type 1 | 346,007(293,386 to 401,853) | 296,776(245,577 to 362,231) | -0·5(-1·0 to -0·1) | 30(25 to 34) | 18(15 to 22) | -1·7(-2·0 to -1·3) |
| Diabetes mellitus type 2 | 3,914,033(3,129,815 to 4,846,143) | 9,603,363(7,588,721 to 11,904,723) | 3·0(2·8 to 3·2) | 436(351 to 536) | 477(376 to 589) | 0·2(0·0 to 0·5) |
| Liver cancer due to NASH | 11,514(2,559 to 26,444) | 13,748(3,220 to 30,821) | 0·5(0·1 to 0·9) | 1(0 to 3) | 1(0 to 1) | -2·4(-2·8 to -2·0) |
| Chronic kidney disease due to diabetes mellitus type 1 | 508,303(362,795 to 673,710) | 493,681(334,382 to 689,359) | -0·1(-0·3 to 0·2) | 45(32 to 60) | 26(18 to 35) | -1·9(-2·1 to -1·6) |
| Chronic kidney disease due to diabetes mellitus type 2 | 849,044(663,737 to 1,032,537) | 1,640,410(1,312,349 to 1,979,868) | 2·4(2·0 to 2·7) | 97(78 to 116) | 83(67 to 99) | -0·5(-0·7 to -0·2) |
| Liver cancer due to other causes | 18,561(4,059 to 43,169) | 15,973(3,654 to 35,838) | -0·6(-0·9 to -0·3) | 2(0 to 5) | 1(0 to 2) | -3·3(-3·7 to -3·0) |
| **High systolic blood pressure** |  |  |  |  |  |  |
| Ischemic stroke | 3,816,004(2,860,358 to 4,858,373) | 10,896,777(8,214,341 to 13,591,421) | 3·7(3·5 to 3·9) | 497(373 to 639) | 564(428 to 706) | 0·4(0·3 to -0·6) |
| Intracerebral hemorrhage | 8,827,091(6,605,936 to 11,547,297) | 12,918,978(9,954,247 to 15,882,085) | 1·4(1·0 to 1·7) | 1,060(805 to 1,369) | 648(503 to 794) | -1·7(-2·0 to -1·4) |
| Subarachnoid hemorrhage | 2,546,734(1,505,588 to 3,356,638) | 1,360,501(1,008,391 to 1,780,303) | -2·2(-2·4 to -2·0) | 299(178 to 392) | 68(50 to 89) | -5·0(-5·4 to -4·6) |
| Chronic kidney disease due to hypertension | 885,572(738,270 to 1,057,438) | 1,692,128(1,393,406 to 2,014,210) | 2·3(2·0 to 2·6) | 101(85 to 119) | 88(73 to 104) | -0·4(-0·7 to -0·2) |
| Chronic kidney disease due to glomerulonephritis | 108,153(65,110 to 159,672) | 242,949(158,056 to 356,225) | 2·9(2·5 to 3·3) | 11(7 to 17) | 12(8 to 18) | 0·3(0·1 to 0·6) |
| Chronic kidney disease due to other and unspecified causes | 206,293(126,425 to 301,866) | 519,888(334,337 to 723,973) | 3·3(3·0 to 3·6) | 25(16 to 36) | 27(18 to 38) | 0·4(0·0 to 0·8) |
| Other cardiomyopathy | 40,000(23,271 to 72,690) | 96,399(65,355 to 132,169) | 3·1(2·8 to 3·3) | 4(3 to 8) | 5(3 to 7) | 0·5(0·2 to 0·7) |
| Non-rheumatic calcific aortic valve disease | 12,614(7,225 to 19,072) | 30,921(22,429 to 41,095) | 3·1(2·9 to 3·3) | 1(1 to 2) | 2(1 to 2) | 0·4(0·2 to 0·7) |
| Chronic kidney disease due to diabetes mellitus type 1 | 105,476(59,390 to 164,430) | 175,781(100,547 to 275,035) | 1·8(1·5 to 2·1) | 10(6 to 16) | 9(5 to 13) | -0·5(-0·8 to -0·2) |
| Chronic kidney disease due to diabetes mellitus type 2 | 273,874(176,991 to 384,732) | 741,688(496,494 to 1,000,436) | 3·6(3·3 to 3·9) | 34(22 to 46) | 37(25 to 50) | 0·4(0·1 to -0·7) |
| **High body-mass index** |  |  |  |  |  |  |
| Liver cancer due to hepatitis B | 254,188(47,127 to 668,425) | 419,201(132,985 to 891,905) | 1·7(1·3 to 2·2) | 25(5 to 66) | 20(6 to 44) | -0·7(-1·2 to -0·3) |
| Liver cancer due to hepatitis C | 39,471(6,900 to 107,001) | 69,719(22,038 to 147,260) | 2·0(1·6 to 2·3) | 4(1 to 12) | 3(1 to 7) | -1·0(-1·4 to -0·6) |
| Liver cancer due to alcohol use | 24,332(4,535 to 68,555) | 49,888(16,025 to 108,856) | 2·6(2·0 to 3·1) | 3(0 to 7) | 2(1 to 5) | -0·3(-0·8 to 0·2) |
| Ischemic stroke | 647,483(161,830 to 1,391,820) | 2,722,593(1,234,050 to 4,594,923) | 5·1(4·8 to 5·3) | 71(18 to 154) | 132(59 to 224) | 2·1(1·9 to 2·4) |
| Intracerebral hemorrhage | 2,102,130(528,378 to 4,511,073) | 4,878,281(2,332,841 to 7,902,616) | 3·0(2·7 to 3·2) | 217(54 to 470) | 237(114 to 385) | 0·3(0·0 to 0·6) |
| Subarachnoid hemorrhage | 626,761(150,418 to 1,372,994) | 587,428(276,406 to 977,359) | -0·2(-0·4 to 0·0) | 63(15 to 140) | 29(14 to 49) | -2·6(-2·9 to -2·3) |
| Chronic kidney disease due to hypertension | 93,106(20,145 to 231,398) | 390,661(141,875 to 725,463) | 5·1(4·9 to 5·4) | 10(2 to 26) | 19(7 to 36) | 2·2(1·9 to 2·4) |
| Chronic kidney disease due to glomerulonephritis | 39,114(6,515 to 107,957) | 139,972(42,497 to 283,703) | 4·6(4·3 to 4·8) | 4(1 to 11) | 7(2 to 14) | 2·0(1·7 to 2·3) |
| Chronic kidney disease due to other and unspecified causes | 69,183(12,392 to 177,973) | 271,682(92,921 to 507,727) | 4·9(4·6 to 5·2) | 8(1 to 19) | 14(5 to 25) | 2·1(1·8 to 2·5) |
| Cataract | 9,122(1,861 to 23,630) | 47,688(15,902 to 101,631) | 5·9(5·7 to 6·2) | 1(0 to 3) | 3(1 to 5) | 2·4(2·2 to 2·5) |
| Acute lymphoid leukemia | 2,986(515 to 8,579) | 16,453(5,695 to 32,771) | 6·1(5·7 to 6·4) | 0(0 to 1) | 1(0 to 2) | 4·3(4·0 to 4·6) |
| Chronic lymphoid leukemia | 1,010(185 to 2,654) | 6,947(2,545 to 13,361) | 6·9(6·5 to 7·3) | 0(0 to 0) | 0(0 to 1) | 4·6(4·3 to 5·0) |
| Acute myeloid leukemia | 2,094(387 to 5,377) | 10,713(3,928 to 20,825) | 5·8(5·6 to 6·0) | 0(0 to 0) | 1(0 to 1) | 3·8(3·6 to 3·9) |
| Chronic myeloid leukemia | 854(162 to 2,249) | 1,719(626 to 3,326) | 2·5(2·1 to 2·8) | 0(0 to 0) | 0(0 to 0) | 0·3(0·0 to 0·6) |
| Other leukemia | 25,575(4,790 to 65,934) | 48,470(17,263 to 96,262) | 2·2(2·0 to 2·5) | 2(0 to 6) | 3(1 to 5) | 0·3(0·0 to 0·6) |
| Diabetes mellitus type 2 | 771,802(209,708 to 1,609,362) | 3,737,576(1,913,966 to 5,903,926) | 5·6(5·5 to 5·7) | 80(21 to 167) | 182(94 to 286) | 2·8(2·7 to 3·0) |
| Chronic kidney disease due to diabetes mellitus type 2 | 95,878(20,284 to 228,095) | 398,042(144,632 to 718,643) | 5·1(4·8 to 5·5) | 11(2 to 25) | 19(7 to 34) | 2·1(1·8 to 2·4) |
| Osteoarthritis hip | 1,115(180 to 3,521) | 7,939(2,173 to 21,603) | 7·1(6·9 to 7·2) | 0(0 to 0) | 0(0 to 1) | 4·0(3·8 to 4·1) |
| Osteoarthritis knee | 98,082(16,592 to 275,584) | 549,964(162,522 to 1,325,608) | 6·1(5·9 to 6·3) | 11(2 to 30) | 26(8 to 62) | 3·0(2·8 to 3·2) |
| Liver cancer due to other causes | 21,798(3,984 to 58,879) | 31,564(10,007 to 65,887) | 1·3(1·0 to 1·7) | 2(0 to 6) | 2(0 to 3) | -1·1(-1·4 to -0·8) |
| **Low bone mineral density** |  |  |  |  |  |  |
| Pedestrian road injuries | 305,195(231,492 to 423,287) | 557,620(443,427 to 663,965) | 2·2(1·7 to 2·6) | 34(26 to 46) | 28(22 to 33) | -0·6(-0·8 to -0·5) |
| Cyclist road injuries | 48,129(34,091 to 63,813) | 145,167(112,878 to 177,574) | 3·9(3·7 to 4·0) | 5(4 to 7) | 7(5 to 9) | 1·0(0·8 to 1·2) |
| Motorcyclist road injuries | 99,307(68,094 to 139,408) | 277,780(212,708 to 343,845) | 3·6(3·5 to 3·7) | 10(7 to 14) | 13(10 to 17) | 0·9(0·7 to 1·1) |
| Motor vehicle road injuries | 79,704(54,672 to 128,572) | 197,633(154,041 to 245,556) | 3·2(2·9 to 3·5) | 8(6 to 13) | 10(7 to 12) | 0·5(0·3 to 0·6) |
| Other road injuries | 5,235(3,946 to 6,695) | 17,659(13,632 to 22,391) | 4·3(4·0 to 4·6) | 1(0 to 1) | 1(1 to 1) | 1·2(1·0 to 1·5) |
| Other exposure to mechanical forces | 63,174(47,142 to 92,266) | 164,611(116,916 to 210,564) | 3·4(2·9 to 3·9) | 7(5 to 10) | 8(6 to 10) | 0·5(0·0 to 0·9) |
| Non-venomous animal contact | 15,552(11,217 to 19,921) | 11,695(8,385 to 17,040) | -1·0(-1·1 to -0·8) | 2(1 to 2) | 1(0 to 1) | -3·9(-4·0 to -3·7) |
| Physical violence by other means | 22,113(17,657 to 27,191) | 30,084(22,865 to 38,560) | 1·1(0·9 to 1·2) | 3(2 to 3) | 1(1 to 2) | -1·9(-2·0 to -1·8) |
| **Kidney dysfunction** |  |  |  |  |  |  |
| Ischemic stroke | 767,687(588,365 to 988,497) | 1,846,012(1,353,662 to 2,370,502) | 3·1(2·9 to 3·3) | 95(70 to 125) | 93(66 to 122) | 0·0(-0·3 to 0·2) |
| Intracerebral hemorrhage | 1,485,834(1,170,187 to 1,904,681) | 1,826,738(1,405,981 to 2,283,554) | 0·7(0·5 to 0·9) | 173(135 to 219) | 91(71 to 114) | -2·1(-2·4 to -1·9) |
| Chronic kidney disease due to hypertension | 885,572(738,270 to 1,057,438) | 1,692,128(1,393,406 to 2,014,210) | 2·3(2·0 to 2·6) | 101(85 to 119) | 88(73 to 104) | -0·4(-0·7 to 0·2) |
| Chronic kidney disease due to glomerulonephritis | 604,415(469,778 to 751,574) | 670,007(522,610 to 839,076) | 0·4(0·1 to 0·7) | 54(43 to 67) | 37(30 to 47) | -1·2(-1·6 to 0·9) |
| Chronic kidney disease due to other and unspecified causes | 1,149,692(949,829 to 1,352,843) | 1,335,618(1,055,379 to 1,664,341) | 0·6(0·3 to 0·9) | 112(93 to 131) | 77(62 to 94) | -1·2(-1·5 to 0·9) |
| Chronic kidney disease due to diabetes mellitus type 1 | 508,303(362,795 to 673,710) | 493,681(334,382 to 689,359) | -0·1(-0·3 to 0·2) | 45(32 to 60) | 26(18 to 35) | -1·9(-2·1 to -1·6) |
| Chronic kidney disease due to diabetes mellitus type 2 | 849,044(663,737 to 1,032,537) | 1,640,410(1,312,349 to 1,979,868) | 2·4(2·0 to 2·7) | 97(78 to 116) | 83(67 to 99) | -0·5(-0·7 to -0·2) |
| **High LDL cholesterol** |  |  |  |  |  |  |
| Ischemic stroke | 1,947,527(1,229,161 to 3,174,413) | 4,427,653(2,418,020 to 7,710,339) | 2·9(2·7 to 3·1) | 239(136 to 416) | 233(120 to 416) | -0·1(-0·3 to 0·1) |
| **deaths** |  |  |  |  |  |  |
| **High fasting plasma glucose** |  |  |  |  |  |  |
| Ischemic stroke | 60,372(30,238 to 112,528) | 174,277(86,292 to 345,963) | 3·6(3·0 to 4·2) | 10(5 to 21) | 10(5 to 21) | 0·0(-0·6 to 0·6) |
| Intracerebral hemorrhage | 111,476(67,082 to 169,216) | 169,412(102,590 to 267,036) | 1·3(0·7 to 1·8) | 16(9 to 26) | 9(6 to 15) | -2·1(-2·6 to -1·5) |
| Subarachnoid hemorrhage | 30,891(16,153 to 49,391) | 14,492(8,426 to 23,059) | -2·7(-3·4 to -2·1) | 4(2 to 7) | 1(0 to 1) | -6·0(-6·6 to -5·3) |
| Drug-susceptible tuberculosis | 11,915(6,753 to 18,099) | 3,307(1,833 to 5,112) | -4·4(-4·9 to -3·9) | 1(1 to 2) | 0(0 to 0) | -7·2(-7·6 to -6·7) |
| Multidrug-resistant tuberculosis without extensive drug resistance | 750(149 to 2,200) | 284(43 to 947) | -3·5(-4·2 to -2·7) | 0(0 to 0) | 0(0 to 0) | -6·3(-7·0 to -5·5) |
| Extensively drug-resistant tuberculosis | 0(0 to 0) | 54(8 to 184) | - | 0(0 to 0) | 0(0 to 0) | - |
| Diabetes mellitus type 1 | 6,005(4,960 to 7,041) | 4,504(3,786 to 5,339) | -0·9(-1·2 to -0·7) | 1(0 to 1) | 0(0 to 0) | -2·6(-2·8 to -2·3) |
| Diabetes mellitus type 2 | 64,084(56,365 to 73,219) | 168,388(143,233 to 194,030) | 3·4(3·3 to 3·6) | 9(8 to 10) | 9(8 to 11) | 0·2(0·1 to 0·4) |
| Liver cancer due to NASH | 471(104 to 1,084) | 629(147 to 1,423) | 0·9(0·5 to 1·3) | 0(0 to 0) | 0(0 to 0) | -2·2(-2·6 to -1·8) |
| Chronic kidney disease due to diabetes mellitus type 1 | 11,183(7,847 to 14,946) | 12,679(8,458 to 18,334) | 0·4(0·2 to 0·7) | 1(1 to 1) | 1(0 to 1) | -1·7(-2·0 to -1·4) |
| Chronic kidney disease due to diabetes mellitus type 2 | 27,413(21,889 to 33,263) | 63,354(49,787 to 77,280) | 2·9(2·8 to 3·1) | 4(3 to 5) | 4(3 to 4) | -0·3(-0·4 to -0·1) |
| Liver cancer due to other causes | 667(149 to 1,527) | 632(145 to 1,424) | -0·3(-0·6 to 0·1) | 0(0 to 0) | 0(0 to 0) | -3·2(-3·6 to -2·8) |
| **High systolic blood pressure** |  |  |  |  |  |  |
| Ischemic stroke | 159,269(116,034 to 208,274) | 486,235(349,990 to 629,761) | 3·9(3·6 to 4·2) | 25(18 to 34) | 28(20 to 37) | 0·4(0·1 to 0·6) |
| Intracerebral hemorrhage | 363,840(269,464 to 480,919) | 588,994(448,853 to 733,707) | 1·7(1·4 to 2·0) | 53(38 to 71) | 32(24 to 41) | -1·7(-2·0 to -1·3) |
| Subarachnoid hemorrhage | 101,544(58,437 to 134,297) | 50,809(36,228 to 68,369) | -2·4(-2·7 to -2·2) | 14(8 to 19) | 3(2 to 4) | -5·6(-5·8 to -5·4) |
| Chronic kidney disease due to hypertension | 28,727(23,475 to 34,466) | 70,260(56,866 to 84,481) | 3·1(3·0 to 3·3) | 4(4 to 5) | 4(3 to 5) | -0·2(-0·3 to 0·0) |
| Chronic kidney disease due to glomerulonephritis | 2,921(1,782 to 4,473) | 7,607(4,813 to 11,455) | 3·4(3·2 to 3·5) | 0(0 to 1) | 0(0 to 1) | 0·3(0·2 to 0·4) |
| Chronic kidney disease due to other and unspecified causes | 5,077(2,967 to 7,792) | 14,978(9,063 to 22,542) | 3·8(3·7 to 4·0) | 1(0 to 1) | 1(1 to 1) | 0·4(0·3 to 0·6) |
| Other cardiomyopathy | 1,327(764 to 2,484) | 3,786(2,520 to 5,284) | 3·7(3·4 to 3·9) | 0(0 to 0) | 0(0 to 0) | 0·7(0·5 to 0·9) |
| Non-rheumatic calcific aortic valve disease | 433(248 to 653) | 1,057(755 to 1,418) | 3·1(2·8 to 3·3) | 0(0 to 0) | 0(0 to 0) | 0·1(-0·2 to 0·3) |
| Chronic kidney disease due to diabetes mellitus type 1 | 2,696(1,520 to 4,208) | 4,991(2,819 to 7,840) | 2·2(1·9 to 2·4) | 0(0 to 0) | 0(0 to 0) | -0·4(-0·6 to -0·2) |
| Chronic kidney disease due to diabetes mellitus type 2 | 9,894(6,579 to 13,814) | 30,124(20,159 to 41,346) | 3·9(3·8 to 4·1) | 2(1 to 2) | 2(1 to 2) | 0·4(0·3 to 0·6) |
| **High body-mass index** |  |  |  |  |  |  |
| Liver cancer due to hepatitis B | 7,347(1,368 to 19,193) | 13,091(4,110 to 28,002) | 2·0(1·6 to 2·4) | 1(0 to 2) | 1(0 to 1) | -0·7(-1·1 to -0·3) |
| Liver cancer due to hepatitis C | 1,552(257 to 4,191) | 3,002(890 to 6,450) | 2·3(1·9 to 2·7) | 0(0 to 1) | 0(0 to 0) | -0·9(-1·3 to -0·5) |
| Liver cancer due to alcohol use | 821(153 to 2,304) | 1,832(586 to 4,043) | 2·9(2·4 to 3·4) | 0(0 to 0) | 0(0 to 0) | -0·1(-0·6 to 0·4) |
| Ischemic stroke | 20,443(4,897 to 44,480) | 87,594(37,019 to 154,794) | 5·1(4·9 to 5·4) | 3(1 to 6) | 4(2 to 8) | 1·9(1·7 to 2·2) |
| Intracerebral hemorrhage | 67,093(16,290 to 147,424) | 162,957(72,331 to 276,642) | 3·1(2·8 to 3·4) | 8(2 to 17) | 8(4 to 14) | 0·2(-0·1 to 0·5) |
| Subarachnoid hemorrhage | 19,128(4,463 to 42,922) | 16,205(7,015 to 28,640) | -0·6(-0·8 to -0·4) | 2(0 to 5) | 1(0 to 1) | -3·3(-3·5 to -3·1) |
| Chronic kidney disease due to hypertension | 3,019(645 to 7,702) | 14,049(4,767 to 27,581) | 5·4(5·2 to 5·6) | 0(0 to 1) | 1(0 to 2) | 2·1(1·9 to 2·4) |
| Chronic kidney disease due to glomerulonephritis | 951(179 to 2,440) | 3,601(1,176 to 7,307) | 4·7(4·5 to 4·9) | 0(0 to 0) | 0(0 to 0) | 1·7(1·5 to 2·0) |
| Chronic kidney disease due to other and unspecified causes | 1,501(263 to 3,972) | 6,306(2,066 to 12,869) | 5·0(4·8 to 5·3) | 0(0 to 0) | 0(0 to 1) | 1·9(1·7 to 2·2) |
| Acute lymphoid leukemia | 74(13 to 206) | 430(150 to 859) | 6·3(6·1 to 6·5) | 0(0 to 0) | 0(0 to 0) | 4·0(3·8 to 4·1) |
| Chronic lymphoid leukemia | 28(5 to 73) | 216(77 to 420) | 7·3(7·0 to 7·6) | 0(0 to 0) | 0(0 to 0) | 4·5(4·1 to 4·9) |
| Acute myeloid leukemia | 55(10 to 142) | 331(120 to 648) | 6·4(6·2 to 6·5) | 0(0 to 0) | 0(0 to 0) | 3·8(3·7 to 4·0) |
| Chronic myeloid leukemia | 24(5 to 61) | 53(19 to 103) | 2·8(2·5 to 3·1) | 0(0 to 0) | 0(0 to 0) | 0·2(-0·1 to 0·5) |
| Other leukemia | 650(124 to 1,651) | 1,505(537 to 3,002) | 2·9(2·7 to 3·2) | 0(0 to 0) | 0(0 to 0) | 0·5(0·3 to 0·7) |
| Diabetes mellitus type 2 | 10,510(2,938 to 21,514) | 47,530(22,514 to 76,632) | 5·3(5·2 to 5·5) | 1(0 to 3) | 2(1 to 4) | 2·3(2·1 to 2·5) |
| Chronic kidney disease due to diabetes mellitus type 2 | 2,979(655 to 7,076) | 13,122(4,496 to 24,541) | 5·2(5·0 to 5·5) | 0(0 to 1) | 1(0 to 1) | 2·0(1·7 to 2·3) |
| Liver cancer due to other causes | 642(115 to 1,706) | 1,040(325 to 2,200) | 1·7(1·3 to 2·1) | 0(0 to 0) | 0(0 to 0) | -1·0(-1·3 to -0·6) |
| **Low bone mineral density** |  |  |  |  |  |  |
| Pedestrian road injuries | 8,676(6,571 to 12,625) | 16,282(12,850 to 19,527) | 2·2(2·0 to 2·4) | 1(1 to 2) | 1(1 to 1) | -0·7(-0·9 to -0·5) |
| Cyclist road injuries | 882(522 to 1,343) | 2,439(1,888 to 3,057) | 3·6(3·5 to 3·7) | 0(0 to 0) | 0(0 to 0) | 0·7(0·5 to 0·9) |
| Motorcyclist road injuries | 1,767(1,083 to 2,802) | 4,128(2,891 to 5,402) | 3·0(2·7 to 3·2) | 0(0 to 0) | 0(0 to 0) | 0·3(0·0 to 0·5) |
| Motor vehicle road injuries | 1,912(1,222 to 3,410) | 4,457(3,280 to 5,731) | 2·9(2·6 to 3·3) | 0(0 to 0) | 0(0 to 0) | 0·2(0·0 to 0·4) |
| Other road injuries | 79(52 to 115) | 218(170 to 270) | 3·5(3·2 to 3·8) | 0(0 to 0) | 0(0 to 0) | 0·5(0·3 to 0·8) |
| Other exposure to mechanical forces | 1,446(1,043 to 2,338) | 3,436(2,092 to 4,376) | 3·1(2·7 to 3·5) | 0(0 to 0) | 0(0 to 0) | 0·1(-0·4 to 0·6) |
| Non-venomous animal contact | 344(221 to 412) | 184(145 to 238) | -2·1(-2·4 to -1·9) | 0(0 to 0) | 0(0 to 0) | -5·0(-5·2 to -4·8) |
| Physical violence by other means | 449(367 to 527) | 366(296 to 447) | -0·7(-0·9 to -0·5) | 0(0 to 0) | 0(0 to 0) | -3·7(-3·9 to -3·6) |
| **Kidney dysfunction** |  |  |  |  |  |  |
| Ischemic stroke | 30,537(22,140 to 40,871) | 77,268(51,264 to 104,740) | 3·3(3·0 to 3·6) | 4(3 to 6) | 4(3 to 6) | -0·1(-0·4 to 0·2) |
| Intracerebral hemorrhage | 57,893(44,649 to 74,893) | 80,860(59,370 to 103,804) | 1·2(0·9 to 1·4) | 8(6 to 11) | 4(3 to 6) | -2·0(-2·3 to -1·7) |
| Chronic kidney disease due to hypertension | 28,727(23,475 to 34,466) | 70,260(56,866 to 84,481) | 3·1(3·0 to 3·3) | 4(4 to 5) | 4(3 to 5) | -0·2(-0·3 to 0·0) |
| Chronic kidney disease due to glomerulonephritis | 11,485(8,771 to 14,968) | 17,390(12,363 to 23,823) | 1·4(1·2 to 1·7) | 1(1 to 2) | 1(1 to 1) | -1·0(-1·2 to -0·8) |
| Chronic kidney disease due to other and unspecified causes | 19,800(15,463 to 25,628) | 33,043(23,283 to 45,006) | 1·8(1·6 to 1·9) | 2(2 to 3) | 2(1 to 3) | -0·8(-1·0 to -0·6) |
| Chronic kidney disease due to diabetes mellitus type 1 | 11,183(7,847 to 14,946) | 12,679(8,458 to 18,334) | 0·4(0·2 to 0·7) | 1(1 to 1) | 1(0 to 1) | -1·7(-2·0 to -1·4) |
| Chronic kidney disease due to diabetes mellitus type 2 | 27,413(21,889 to 33,263) | 63,354(49,787 to 77,280) | 2·9(2·8 to 3·1) | 4(3 to 5) | 4(3 to 4) | -0·3(-0·4 to -0·1) |
| **High LDL cholesterol** |  |  |  |  |  |  |
| Ischemic stroke | 67,806(33,489 to 130,613) | 182,689(71,552 to 383,129) | 3·5(3·2 to 3·7) | 11(4 to 23) | 11(4 to 24) | 0·0(-0·3 to 0·3) |

AAPC, average annual percent changes; DALYs, disability-adjusted life years; UI, uncertainty interval; CI, confidence interval.

**sTable 4.** Trend in number and age-standardized rate of DALYs and deaths for the most detailed causes attributable to six metabolic risk factors in China, 1990-2019

|  | Number | | | Age standardized rate per 100,000 population | | |
| --- | --- | --- | --- | --- | --- | --- |
|  | 1990 | 2019 | AAPC (95% CI) | 1990 | 2019 | AAPC (95% CI) |
| **DALYs** |  |  |  |  |  |  |
| **High fasting plasma glucose** |  |  |  |  |  |  |
| Tracheal, bronchus, and lung cancer | 345,569(72,605 to 813,014) | 1,107,654(242,107 to 2,548,449) | 4·1(3·8 to 4·3) | 40(9 to 94) | 192(100 to 354) | 1·0(0·8 to 1·2) |
| Breast cancer | 55,063(10,119 to 128,018) | 146,739(26,655 to 348,416) | 3·4(3·1 to 3·7) | 6(1 to 14) | 176(111 to 266) | 0·4(0·1 to 0·7) |
| Colon and rectum cancer | 104,239(24,054 to 240,404) | 395,340(85,260 to 915,610) | 4·7(4·4 to 5·0) | 12(3 to 29) | 18(11 to 27) | 1·5(1·2 to 1·8) |
| Pancreatic cancer | 36,617(7,793 to 85,001) | 175,495(38,289 to 404,020) | 5·5(5·2 to 5·8) | 4(1 to 10) | 0(0 to 1) | 2·4(2·1 to 2·7) |
| Ovarian cancer | 10,399(1,883 to 27,641) | 44,615(7,595 to 113,363) | 5·0(4·6 to 5·4) | 1(0 to 3) | 4(1 to 10) | 2·0(1·6 to 2·4) |
| Bladder cancer | 21,474(4,273 to 49,136) | 55,650(10,510 to 124,278) | 3·2(2·7 to 3·8) | 3(1 to 7) | 6(3 to 9) | -0·1(-0·6 to 0·4) |
| Ischemic heart disease | 2,072,260(1,277,153 to 3,222,225) | 6,014,932(3,774,590 to 9,306,629) | 3·6(3·0 to 4·3) | 277(171 to 428) | 0(0 to 1) | 0·5(-0·1 to 1·1) |
| Ischemic stroke | 1,352,615(724,692 to 2,398,297) | 3,667,314(1,936,682 to 6,510,542) | 3·4(2·8 to 4·0) | 183(95 to 341) | 0(0 to 0) | 0·1(-0·5 to 0·6) |
| Intracerebral hemorrhage | 2,504,782(1,535,000 to 3,700,369) | 3,506,649(2,231,722 to 5,211,237) | 1·0(0·5 to 1·5) | 308(191 to 455) | 0(0 to 0) | -2·1(-2·6 to -1·7) |
| Subarachnoid hemorrhage | 713,226(384,912 to 1,112,571) | 359,268(220,875 to 533,972) | -2·5(-3·0 to -1·9) | 86(46 to 135) | 18(15 to 22) | -5·4(-5·9 to -4·9) |
| Peripheral artery disease | 14,589(7,937 to 24,584) | 37,686(21,305 to 62,767) | 3·2(2·9 to 3·6) | 2(1 to 3) | 477(376 to 589) | -0·2(-0·5 to 0·2) |
| Alzheimer's disease and other dementias | 124,438(18,782 to 445,419) | 442,695(73,488 to 1,425,147) | 4·4(4·2 to 4·6) | 24(4 to 85) | 1(0 to 1) | 0·4(0·2 to 0·4) |
| Glaucoma | 4,432(1,018 to 10,381) | 8,225(1,874 to 19,370) | 2·2(1·8 to 2·5) | 1(0 to 2) | 26(18 to 35) | -1·5(-1·7 to -1·2) |
| Cataract | 27,247(5,656 to 66,204) | 76,689(15,737 to 182,771) | 3·7(3·5 to 3·9) | 4(1 to 10) | 83(67 to 99) | 0·0(-0·2 to 0·2) |
| Drug-susceptible tuberculosis | 367,193(221,840 to 545,717) | 115,440(67,663 to 171,861) | -4·1(-4·5 to -3·6) | 40(24 to 58) | 1(0 to 2) | -6·5(-6·9 to -6·1) |
| Multidrug-resistant tuberculosis without extensive drug resistance | 22,075(4,543 to 64,377) | 8,589(1,406 to 26,962) | -3·4(-4·1 to -2·7) | 2(0 to 7) | 564(428 to 706) | -5·9(-6·6 to -5·3) |
| Extensively drug-resistant tuberculosis | 0(0 to 0) | 1,509(232 to 4,684) | - | 0(0 to 0) | 648(503 to 794) | - |
| Latent tuberculosis infection | 0(0 to 0) | 0(0 to 0) | - | 0(0 to 0) | 68(50 to 89) | - |
| Diabetes mellitus type 1 | 346,007(293,386 to 401,853) | 296,776(245,577 to 362,231) | -0·5(-1·0 to -0·1) | 30(25 to 34) | 88(73 to 104) | -1·7(-2·0 to -1·3) |
| Diabetes mellitus type 2 | 3,914,033(3,129,815 to 4,846,143) | 9,603,363(7,588,721 to 11,904,723) | 3·0(2·8 to 3·2) | 436(351 to 536) | 12(8 to 18) | 0·2(0·0 to 0·5) |
| Liver cancer due to NASH | 11,514(2,559 to 26,444) | 13,748(3,220 to 30,821) | 0·5(0·1 to 0·9) | 1(0 to 3) | 27(18 to 38) | -2·4(-2·8 to -2·0) |
| Chronic kidney disease due to diabetes mellitus type 1 | 508,303(362,795 to 673,710) | 493,681(334,382 to 689,359) | -0·1(-0·3 to 0·2) | 45(32 to 60) | 5(3 to 7) | -1·9(-2·1 to -1·6) |
| Chronic kidney disease due to diabetes mellitus type 2 | 849,044(663,737 to 1,032,537) | 1,640,410(1,312,349 to 1,979,868) | 2·4(2·0 to 2·7) | 97(78 to 116) | 2(1 to 2) | -0·5(-0·7 to -0·2) |
| Liver cancer due to other causes | 18,561(4,059 to 43,169) | 15,973(3,654 to 35,838) | -0·6(-0·9 to -0·3) | 2(0 to 5) | 9(5 to 13) | -3·3(-3·7 to -3·0) |
| Total burden related to Non-alcoholic fatty liver disease (NAFLD) | 11,514(2,559 to 26,444) | 13,748(3,220 to 30,821) | 0·5(0·1 to 0·9) | 1(0 to 3) | 37(25 to 50) | -2·4(-2·8 to -2·0) |
| Total cancers | 603,435(152,503 to 1,309,632) | 1,955,214(496,063 to 4,292,456) | 4·1(3·9 to 4·3) | 70(18 to 151) | 20(6 to 44) | 1·0(0·8 to 1·3) |
| **High systolic blood pressure** |  |  |  |  |  |  |
| Rheumatic heart disease | 742,250(462,441 to 1,155,465) | 450,558(294,885 to 694,341) | -1·7(-2·0 to -1·5) | 83(52 to 137) | 3(1 to 7) | -4·3(-4·5 to -4·1) |
| Ischemic heart disease | 6,332,425(4,950,620 to 7,809,007) | 18,552,713(14,741,741 to 22,628,942) | 3·8(3·5 to 4·0) | 797(628 to 988) | 2(1 to 5) | 0·7(0·5 to 1·0) |
| Ischemic stroke | 3,816,004(2,860,358 to 4,858,373) | 10,896,777(8,214,341 to 13,591,421) | 3·7(3·5 to 3·9) | 497(373 to 639) | 132(59 to 224) | 0·4(0·3 to 0·6) |
| Intracerebral hemorrhage | 8,827,091(6,605,936 to 11,547,297) | 12,918,978(9,954,247 to 15,882,085) | 1·4(1·0 to 1·7) | 1,060(805 to 1,369) | 237(114 to 385) | -1·7(-2·0 to -1·4) |
| Subarachnoid hemorrhage | 2,546,734(1,505,588 to 3,356,638) | 1,360,501(1,008,391 to 1,780,303) | -2·2(-2·4 to -2·0) | 299(178 to 392) | 29(14 to 49) | -5·0(-5·4 to -4·6) |
| Hypertensive heart disease | 5,042,174(3,661,268 to 5,792,671) | 5,594,910(3,877,275 to 6,532,987) | 0·4(0·1 to 0·7) | 693(526 to 786) | 19(7 to 36) | -2·7(-3·0 to -2·4) |
| Atrial fibrillation and flutter | 217,091(150,778 to 300,060) | 700,754(494,049 to 953,254) | 4·1(4·0 to 4·2) | 32(22 to 44) | 7(2 to 14) | 0·6(0·5 to 0·7) |
| Aortic aneurysm | 52,450(35,374 to 75,857) | 141,700(108,068 to 182,227) | 3·5(3·3 to 3·6) | 6(4 to 9) | 14(5 to 25) | 0·6(0·4 to 0·7) |
| Peripheral artery disease | 15,956(8,181 to 28,066) | 47,540(25,117 to 84,157) | 3·8(3·8 to 3·9) | 2(1 to 4) | 3(1 to 5) | 0·3(0·3 to 0·4) |
| Endocarditis | 22,296(13,987 to 31,119) | 38,546(27,170 to 52,764) | 1·9(1·7 to 2·2) | 2(2 to 3) | 1(0 to 2) | -0·6(-1·0 to -0·3) |
| Chronic kidney disease due to hypertension | 885,572(738,270 to 1,057,438) | 1,692,128(1,393,406 to 2,014,210) | 2·3(2·0 to 2·6) | 101(85 to 119) | 0(0 to 1) | -0·4(-0·7 to -0·2) |
| Chronic kidney disease due to glomerulonephritis | 108,153(65,110 to 159,672) | 242,949(158,056 to 356,225) | 2·9(2·5 to 3·3) | 11(7 to 17) | 1(0 to 1) | 0·3(0·1 to 0·6) |
| Chronic kidney disease due to other and unspecified causes | 206,293(126,425 to 301,866) | 519,888(334,337 to 723,973) | 3·3(3·0 to 3·6) | 25(16 to 36) | 0(0 to 0) | 0·4(0·0 to 0·8) |
| Other cardiomyopathy | 40,000(23,271 to 72,690) | 96,399(65,355 to 132,169) | 3·1(2·8 to 3·3) | 4(3 to 8) | 3(1 to 5) | 0·5(0·2 to 0·7) |
| Non-rheumatic calcific aortic valve disease | 12,614(7,225 to 19,072) | 30,921(22,429 to 41,095) | 3·1(2·9 to 3·3) | 1(1 to 2) | 182(94 to 286) | 0·4(0·2 to 0·7) |
| Chronic kidney disease due to diabetes mellitus type 1 | 105,476(59,390 to 164,430) | 175,781(100,547 to 275,035) | 1·8(1·5 to 2·1) | 10(6 to 16) | 19(7 to 34) | -0·5(-0·8 to -0·2) |
| Chronic kidney disease due to diabetes mellitus type 2 | 273,874(176,991 to 384,732) | 741,688(496,494 to 1,000,436) | 3·6(3·3 to 3·9) | 34(22 to 46) | 0(0 to 1) | 0·4(0·1 to 0·7) |
| Other cardiovascular and circulatory diseases | 125,952(94,847 to 167,652) | 238,883(185,268 to 297,014) | 2·2(2·1 to 2·4) | 15(11 to 20) | 26(8 to 62) | -0·6(-0·8 to -0·4) |
| **High body-mass index** |  |  |  |  |  |  |
| Esophageal cancer | 306,709(49,525 to 827,903) | 859,654(221,497 to 1,875,382) | 3·6(3·4 to 3·8) | 34(6 to 93) | 41(11 to 89) | 0·6(0·4 to 0·8) |
| Liver cancer due to hepatitis B | 254,188(47,127 to 668,425) | 419,201(132,985 to 891,905) | 1·7(1·3 to 2·2) | 25(5 to 66) | 20(6 to 44) | -0·7(-1·2 to -0·3) |
| Liver cancer due to hepatitis C | 39,471(6,900 to 107,001) | 69,719(22,038 to 147,260) | 2·0(1·6 to 2·3) | 4(1 to 12) | 3(1 to 7) | -1·0(-1·4 to -0·6) |
| Liver cancer due to alcohol use | 24,332(4,535 to 68,555) | 49,888(16,025 to 108,856) | 2·6(2·0 to 3·1) | 3(0 to 7) | 2(1 to 5) | -0·3(-0·8 to 0·2) |
| Breast cancer | 62,298(11,394 to 150,487) | 336,583(93,779 to 680,214) | 5·9(5·5 to 6·3) | 7(1 to 17) | 15(4 to 31) | 2·8(2·6 to 3·1) |
| Uterine cancer | 38,378(9,764 to 83,099) | 90,829(40,885 to 166,472) | 3·0(2·8 to 3·3) | 4(1 to 9) | 4(2 to 8) | 0·3(0·1 to 0·4) |
| Colon and rectum cancer | 53,125(11,759 to 127,189) | 376,133(149,260 to 689,708) | 7·0(6·8 to 7·1) | 6(1 to 14) | 18(7 to 34) | 4·1(4·0 to 4·3) |
| Gallbladder and biliary tract cancer | 16,054(3,730 to 40,845) | 81,473(31,251 to 157,725) | 5·8(5·5 to 6·0) | 2(0 to 5) | 4(2 to 8) | 2·7(2·5 to 2·9) |
| Pancreatic cancer | 12,709(2,179 to 34,463) | 106,354(27,250 to 240,617) | 7·6(7·4 to 7·7) | 1(0 to 4) | 5(1 to 12) | 4·6(4·4 to 4·8) |
| Ovarian cancer | 2,352(-53 to 7,379) | 16,701(-398 to 43,257) | 7·0(6·9 to 7·1) | 0(0 to 1) | 1(0 to 2) | 4·2(4·1 to 4·3) |
| Kidney cancer | 8,117(1,906 to 18,424) | 70,544(29,312 to 127,229) | 7·7(7·6 to 7·9) | 1(0 to 2) | 3(1 to 6) | 4·9(4·7 to 5·0) |
| Thyroid cancer | 3,227(682 to 7,943) | 14,811(4,839 to 30,476) | 5·3(5·1 to 5·6) | 0(0 to 1) | 1(0 to 2) | 2·7(2·5 to 2·8) |
| Non-Hodgkin lymphoma | 7,344(1,462 to 19,495) | 47,354(16,139 to 99,617) | 6·7(6·3 to 7·0) | 1(0 to 2) | 2(1 to 5) | 4·1(3·7 to 4·4) |
| Multiple myeloma | 2,828(524 to 7,430) | 14,687(4,528 to 30,414) | 5·8(5·6 to 6·0) | 0(0 to 1) | 1(0 to 1) | 3·0(2·8 to 3·2) |
| Ischemic heart disease | 1,183,844(283,868 to 2,643,152) | 5,073,254(2,233,964 to 8,744,122) | 5·2(5·0 to 5·4) | 128(30 to 286) | 258(112 to 447) | 2·5(2·3 to 2·6) |
| Ischemic stroke | 647,483(161,830 to 1,391,820) | 2,722,593(1,234,050 to 4,594,923) | 5·1(4·8 to 5·3) | 71(18 to 154) | 132(59 to 224) | 2·1(1·9 to 2·4) |
| Intracerebral hemorrhage | 2,102,130(528,378 to 4,511,073) | 4,878,281(2,332,841 to 7,902,616) | 3·0(2·7 to 3·2) | 217(54 to 470) | 237(114 to 385) | 0·3(0·0 to 0·6) |
| Subarachnoid hemorrhage | 626,761(150,418 to 1,372,994) | 587,428(276,406 to 977,359) | -0·2(-0·4 to 0·0) | 63(15 to 140) | 29(14 to 49) | -2·6(-2·9 to -2·3) |
| Hypertensive heart disease | 727,467(176,938 to 1,575,574) | 1,514,532(673,366 to 2,626,321) | 2·5(2·0 to 3·0) | 92(21 to 208) | 80(34 to 145) | -0·5(-1·0 to 0·0) |
| Atrial fibrillation and flutter | 40,270(8,841 to 97,700) | 228,050(88,027 to 445,218) | 6·2(6·1 to 6·2) | 6(1 to 15) | 12(5 to 24) | 2·5(2·4 to 2·6) |
| Asthma | 98,457(25,467 to 228,184) | 175,325(75,052 to 320,742) | 2·1(1·9 to 2·2) | 11(3 to 26) | 10(5 to 19) | -0·1(-0·3 to 0·2) |
| Gallbladder and biliary diseases | 139,950(32,648 to 342,137) | 384,258(158,489 to 746,027) | 3·5(3·4 to 3·6) | 15(4 to 37) | 19(8 to 37) | 0·8(0·7 to 0·9) |
| Alzheimer's disease and other dementias | 72,969(8,730 to 252,707) | 477,209(101,059 to 1,388,766) | 6·7(6·6 to 6·8) | 13(2 to 46) | 28(6 to 82) | 2·7(2·6 to 2·8) |
| Chronic kidney disease due to hypertension | 93,106(20,145 to 231,398) | 390,661(141,875 to 725,463) | 5·1(4·9 to 5·4) | 10(2 to 26) | 19(7 to 36) | 2·2(1·9 to 2·4) |
| Chronic kidney disease due to glomerulonephritis | 39,114(6,515 to 107,957) | 139,972(42,497 to 283,703) | 4·6(4·3 to 4·8) | 4(1 to 11) | 7(2 to 14) | 2·0(1·7 to 2·3) |
| Chronic kidney disease due to other and unspecified causes | 69,183(12,392 to 177,973) | 271,682(92,921 to 507,727) | 4·9(4·6 to 5·2) | 8(1 to 19) | 14(5 to 25) | 2·1(1·8 to 2·5) |
| Low back pain | 155,486(33,389 to 376,821) | 460,685(184,490 to 899,388) | 3·8(3·7 to 3·9) | 15(3 to 37) | 23(9 to 46) | 1·4(1·3 to 1·6) |
| Gout | 18,680(4,003 to 47,523) | 115,104(44,197 to 231,850) | 6·5(6·3 to 6·7) | 2(0 to 5) | 6(2 to 11) | 3·8(3·6 to 4·0) |
| Cataract | 9,122(1,861 to 23,630) | 47,688(15,902 to 101,631) | 5·9(5·7 to 6·2) | 1(0 to 3) | 3(1 to 5) | 2·4(2·2 to 2·5) |
| Acute lymphoid leukemia | 2,986(515 to 8,579) | 16,453(5,695 to 32,771) | 6·1(5·7 to 6·4) | 0(0 to 1) | 1(0 to 2) | 4·3(4·0 to 4·6) |
| Chronic lymphoid leukemia | 1,010(185 to 2,654) | 6,947(2,545 to 13,361) | 6·9(6·5 to 7·3) | 0(0 to 0) | 0(0 to 1) | 4·6(4·3 to 5·0) |
| Acute myeloid leukemia | 2,094(387 to 5,377) | 10,713(3,928 to 20,825) | 5·8(5·6 to 6·0) | 0(0 to 0) | 1(0 to 1) | 3·8(3·6 to 3·9) |
| Chronic myeloid leukemia | 854(162 to 2,249) | 1,719(626 to 3,326) | 2·5(2·1 to 2·8) | 0(0 to 0) | 0(0 to 0) | 0·3(0·0 to 0·6) |
| Other leukemia | 25,575(4,790 to 65,934) | 48,470(17,263 to 96,262) | 2·2(2·0 to 2·5) | 2(0 to 6) | 3(1 to 5) | 0·3(0·0 to 0·6) |
| Diabetes mellitus type 2 | 771,802(209,708 to 1,609,362) | 3,737,576(1,913,966 to 5,903,926) | 5·6(5·5 to 5·7) | 80(21 to 167) | 182(94 to 286) | 2·8(2·7 to 3·0) |
| Chronic kidney disease due to diabetes mellitus type 2 | 95,878(20,284 to 228,095) | 398,042(144,632 to 718,643) | 5·1(4·8 to 5·5) | 11(2 to 25) | 19(7 to 34) | 2·1(1·8 to 2·4) |
| Osteoarthritis hip | 1,115(180 to 3,521) | 7,939(2,173 to 21,603) | 7·1(6·9 to 7·2) | 0(0 to 0) | 0(0 to 1) | 4·0(3·8 to 4·1) |
| Osteoarthritis knee | 98,082(16,592 to 275,584) | 549,964(162,522 to 1,325,608) | 6·1(5·9 to 6·3) | 11(2 to 30) | 26(8 to 62) | 3·0(2·8 to 3·2) |
| Liver cancer due to other causes | 21,798(3,984 to 58,879) | 31,564(10,007 to 65,887) | 1·3(1·0 to 1·7) | 2(0 to 6) | 2(0 to 3) | -1·1(-1·4 to -0·8) |
| Total burden related to hepatitis B | 254,188(47,127 to 668,425) | 419,201(132,985 to 891,905) | 1·7(1·3 to 2·2) | 25(5 to 66) | 20(6 to 44) | -0·7(-1·2 to -0·3) |
| Total burden related to hepatitis C | 39,471(6,900 to 107,001) | 69,719(22,038 to 147,260) | 2·0(1·6 to 2·3) | 4(1 to 12) | 3(1 to 7) | -1·0(-1·4 to -0·6) |
| Total cancers | 885,448(194,534 to 2,154,883) | 2,669,799(1,094,208 to 4,839,179) | 3·9(3·7 to 4·1) | 94(21 to 227) | 128(53 to 233) | 1·1(0·9 to 1·2) |
| **Low bone mineral density** |  |  |  |  |  |  |
| Pedestrian road injuries | 305,195(231,492 to 423,287) | 557,620(443,427 to 663,965) | 2·2(1·7 to 2·6) | 34(26 to 46) | 28(22 to 33) | -0·6(-0·8 to -0·5) |
| Cyclist road injuries | 48,129(34,091 to 63,813) | 145,167(112,878 to 177,574) | 3·9(3·7 to 4·0) | 5(4 to 7) | 7(5 to 9) | 1·0(0·8 to 1·2) |
| Motorcyclist road injuries | 99,307(68,094 to 139,408) | 277,780(212,708 to 343,845) | 3·6(3·5 to 3·7) | 10(7 to 14) | 13(10 to 17) | 0·9(0·7 to 1·1) |
| Motor vehicle road injuries | 79,704(54,672 to 128,572) | 197,633(154,041 to 245,556) | 3·2(2·9 to 3·5) | 8(6 to 13) | 10(7 to 12) | 0·5(0·3 to 0·6) |
| Other road injuries | 5,235(3,946 to 6,695) | 17,659(13,632 to 22,391) | 4·3(4·0 to 4·6) | 1(0 to 1) | 1(1 to 1) | 1·2(1·0 to 1·5) |
| Other transport injuries | 80,275(64,852 to 103,780) | 78,651(63,197 to 94,695) | -0·1(-0·6 to 0·3) | 9(7 to 11) | 4(3 to 5) | -2·9(-3·3 to -2·5) |
| Falls | 620,852(503,823 to 753,230) | 1,839,375(1,346,044 to 2,316,329) | 3·8(3·7 to 4·0) | 90(74 to 109) | 105(76 to 131) | 0·5(0·4 to 0·7) |
| Other exposure to mechanical forces | 63,174(47,142 to 92,266) | 164,611(116,916 to 210,564) | 3·4(2·9 to 3·9) | 7(5 to 10) | 8(6 to 10) | 0·5(0·0 to 0·9) |
| Non-venomous animal contact | 15,552(11,217 to 19,921) | 11,695(8,385 to 17,040) | -1·0(-1·1 to -0·8) | 2(1 to 2) | 1(0 to 1) | -3·9(-4·0 to -3·7) |
| Physical violence by other means | 22,113(17,657 to 27,191) | 30,084(22,865 to 38,560) | 1·1(0·9 to 1·2) | 3(2 to 3) | 1(1 to 2) | -1·9(-2·0 to -1·8) |
| **Kidney dysfunction** |  |  |  |  |  |  |
| Ischemic heart disease | 1,405,191(931,472 to 1,946,778) | 3,776,976(2,431,295 to 5,253,210) | 3·5(3·0 to 4·0) | 182(117 to 254) | 209(134 to 291) | 0·5(0·0 to 1·0) |
| Ischemic stroke | 767,687(588,365 to 988,497) | 1,846,012(1,353,662 to 2,370,502) | 3·1(2·9 to 3·3) | 95(70 to 125) | 93(66 to 122) | 0·0(-0·3 to 0·2) |
| Intracerebral hemorrhage | 1,485,834(1,170,187 to 1,904,681) | 1,826,738(1,405,981 to 2,283,554) | 0·7(0·5 to 0·9) | 173(135 to 219) | 91(71 to 114) | -2·1(-2·4 to -1·9) |
| Peripheral artery disease | 10,458(5,983 to 17,378) | 25,816(14,645 to 44,192) | 3·2(3·0 to 3·4) | 1(1 to 2) | 1(1 to 2) | 0·0(0·0 to 0·1) |
| Chronic kidney disease due to hypertension | 885,572(738,270 to 1,057,438) | 1,692,128(1,393,406 to 2,014,210) | 2·3(2·0 to 2·6) | 101(85 to 119) | 88(73 to 104) | -0·4(-0·7 to -0·2) |
| Chronic kidney disease due to glomerulonephritis | 604,415(469,778 to 751,574) | 670,007(522,610 to 839,076) | 0·4(0·1 to 0·7) | 54(43 to 67) | 37(30 to 47) | -1·2(-1·6 to -0·9) |
| Chronic kidney disease due to other and unspecified causes | 1,149,692(949,829 to 1,352,843) | 1,335,618(1,055,379 to 1,664,341) | 0·6(0·3 to 0·9) | 112(93 to 131) | 77(62 to 94) | -1·2(-1·5 to -0·9) |
| Gout | 12,654(7,655 to 18,640) | 46,523(28,108 to 68,591) | 4·6(4·5 to 4·8) | 2(1 to 2) | 2(1 to 4) | 1·3(1·1 to 1·5) |
| Chronic kidney disease due to diabetes mellitus type 1 | 508,303(362,795 to 673,710) | 493,681(334,382 to 689,359) | -0·1(-0·3 to 0·2) | 45(32 to 60) | 26(18 to 35) | -1·9(-2·1 to -1·6) |
| Chronic kidney disease due to diabetes mellitus type 2 | 849,044(663,737 to 1,032,537) | 1,640,410(1,312,349 to 1,979,868) | 2·4(2·0 to 2·7) | 97(78 to 116) | 83(67 to 99) | -0·5(-0·7 to -0·2) |
| **High LDL cholesterol** |  |  |  |  |  |  |
| Ischemic heart disease | 6,941,572(5,615,925 to 8,542,968) | 15,386,309(11,920,599 to 19,336,537) | 2·8(2·6 to 2·9) | 807(637 to 1,003) | 819(629 to 1,041) | 0·1(-0·1 to 0·3) |
| Ischemic stroke | 1,947,527(1,229,161 to 3,174,413) | 4,427,653(2,418,020 to 7,710,339) | 2·9(2·7 to 3·1) | 239(136 to 416) | 233(120 to 416) | -0·1(-0·3 to 0·1) |
| **deaths** |  |  |  |  |  |  |
| **High fasting plasma glucose** |  |  |  |  |  |  |
| Tracheal, bronchus, and lung cancer | 14,427(3,113 to 33,824) | 53,005(11,785 to 121,265) | 4·6(4·3 to 4·8) | 2(0 to 4) | 3(1 to 6) | 1·3(1·1 to 1·6) |
| Breast cancer | 1,931(362 to 4,510) | 5,453(1,003 to 12,882) | 3·6(3·3 to 3·9) | 0(0 to 1) | 0(0 to 1) | 0·4(0·1 to 0·8) |
| Colon and rectum cancer | 4,423(1,025 to 10,166) | 18,161(4,030 to 41,451) | 5·0(4·6 to 5·3) | 1(0 to 1) | 1(0 to 2) | 1·5(1·2 to 1·9) |
| Pancreatic cancer | 1,523(323 to 3,532) | 8,046(1,759 to 18,320) | 5·9(5·6 to 6·2) | 0(0 to 0) | 0(0 to 1) | 2·5(2·2 to 2·9) |
| Ovarian cancer | 385(70 to 1,024) | 1,758(299 to 4,429) | 5·2(4·9 to 5·6) | 0(0 to 0) | 0(0 to 0) | 2·0(1·6 to 2·4) |
| Bladder cancer | 1,061(213 to 2,421) | 2,970(565 to 6,595) | 3·5(3·0 to 4·0) | 0(0 to 0) | 0(0 to 0) | -0·1(-0·6 to 0·4) |
| Ischemic heart disease | 95,190(58,410 to 149,663) | 341,686(193,747 to 557,999) | 4·4(3·8 to 5·0) | 16(9 to 26) | 21(11 to 35) | 0·7(0·2 to 1·2) |
| Ischemic stroke | 60,372(30,238 to 112,528) | 174,277(86,292 to 345,963) | 3·6(3·0 to 4·2) | 10(5 to 21) | 10(5 to 21) | 0·0(-0·6 to 0·6) |
| Intracerebral hemorrhage | 111,476(67,082 to 169,216) | 169,412(102,590 to 267,036) | 1·3(0·7 to 1·8) | 16(9 to 26) | 9(6 to 15) | -2·1(-2·6 to -1·5) |
| Subarachnoid hemorrhage | 30,891(16,153 to 49,391) | 14,492(8,426 to 23,059) | -2·7(-3·4 to -2·1) | 4(2 to 7) | 1(0 to 1) | -6·0(-6·6 to -5·3) |
| Peripheral artery disease | 123(94 to 175) | 474(372 to 606) | 4·7(4·0 to 5·3) | 0(0 to 0) | 0(0 to 0) | 0·9(0·4 to 1·4) |
| Alzheimer's disease and other dementias | 6,521(629 to 26,296) | 23,988(2,536 to 94,088) | 4·6(4·3 to 4·8) | 2(0 to 7) | 2(0 to 7) | 0·1(-0·1 to 0·3) |
| Drug-susceptible tuberculosis | 11,915(6,753 to 18,099) | 3,307(1,833 to 5,112) | -4·4(-4·9 to -3·9) | 1(1 to 2) | 0(0 to 0) | -7·2(-7·6 to -6·7) |
| Multidrug-resistant tuberculosis without extensive drug resistance | 750(149 to 2,200) | 284(43 to 947) | -3·5(-4·2 to -2·7) | 0(0 to 0) | 0(0 to 0) | -6·3(-7·0 to -5·5) |
| Extensively drug-resistant tuberculosis | 0(0 to 0) | 54(8 to 184) | - | 0(0 to 0) | 0(0 to 0) | - |
| Diabetes mellitus type 1 | 6,005(4,960 to 7,041) | 4,504(3,786 to 5,339) | -0·9(-1·2 to -0·7) | 1(0 to 1) | 0(0 to 0) | -2·6(-2·8 to -2·3) |
| Diabetes mellitus type 2 | 64,084(56,365 to 73,219) | 168,388(143,233 to 194,030) | 3·4(3·3 to 3·6) | 9(8 to 10) | 9(8 to 11) | 0·2(0·1 to 0·4) |
| Liver cancer due to NASH | 471(104 to 1,084) | 629(147 to 1,423) | 0·9(0·5 to 1·3) | 0(0 to 0) | 0(0 to 0) | -2·2(-2·6 to -1·8) |
| Chronic kidney disease due to diabetes mellitus type 1 | 11,183(7,847 to 14,946) | 12,679(8,458 to 18,334) | 0·4(0·2 to 0·7) | 1(1 to 1) | 1(0 to 1) | -1·7(-2·0 to -1·4) |
| Chronic kidney disease due to diabetes mellitus type 2 | 27,413(21,889 to 33,263) | 63,354(49,787 to 77,280) | 2·9(2·8 to 3·1) | 4(3 to 5) | 4(3 to 4) | -0·3(-0·4 to -0·1) |
| Liver cancer due to other causes | 667(149 to 1,527) | 632(145 to 1,424) | -0·3(-0·6 to 0·1) | 0(0 to 0) | 0(0 to 0) | -3·2(-3·6 to -2·8) |
| Total burden related to Non-alcoholic fatty liver disease (NAFLD) | 471(104 to 1,084) | 629(147 to 1,423) | 0·9(0·5 to 1·3) | 0(0 to 0) | 0(0 to 0) | -2·2(-2·6 to -1·8) |
| Total cancers | 24,887(6,343 to 53,939) | 90,655(23,078 to 197,161) | 4·5(4·3 to 4·8) | 3(1 to 7) | 5(1 to 10) | 1·2(1·0 to 1·5) |
| **High systolic blood pressure** |  |  |  |  |  |  |
| Rheumatic heart disease | 27,138(16,393 to 46,065) | 18,226(11,270 to 29,323) | -1·4(-1·6 to -1·2) | 4(2 to 7) | 1(1 to 2) | -4·4(-4·7 to -4·1) |
| Ischemic heart disease | 265,786(206,604 to 329,317) | 969,221(741,700 to 1,217,123) | 4·5(4·3 to 4·8) | 43(32 to 55) | 59(43 to 75) | 1·1(0·8 to 1·3) |
| Ischemic stroke | 159,269(116,034 to 208,274) | 486,235(349,990 to 629,761) | 3·9(3·6 to 4·2) | 25(18 to 34) | 28(20 to 37) | 0·4(0·1 to 0·6) |
| Intracerebral hemorrhage | 363,840(269,464 to 480,919) | 588,994(448,853 to 733,707) | 1·7(1·4 to 2·0) | 53(38 to 71) | 32(24 to 41) | -1·7(-2·0 to -1·3) |
| Subarachnoid hemorrhage | 101,544(58,437 to 134,297) | 50,809(36,228 to 68,369) | -2·4(-2·7 to -2·2) | 14(8 to 19 ) | 3(2 to 4 ) | -5·6(-5·8 to -5·4) |
| Hypertensive heart disease | 241,298(180,926 to 275,423) | 320,090(201,652 to 373,927) | 1·0(0·6 to 1·4) | 42(34 to 48 ) | 21(13 to 24 ) | -2·4(-2·8 to -2·0) |
| Atrial fibrillation and flutter | 5,013(3,642 to 6,634) | 17,513(13,026 to 23,149) | 4·4(4·1 to 4·6) | 1(1 to 2 ) | 1(1 to 2 ) | 0·2(0·1 to 0·4) |
| Aortic aneurysm | 2,075(1,408 to 2,971) | 6,083(4,584 to 7,789) | 3·7(3·6 to 3·9) | 0(0 to 0 ) | 0(0 to 0 ) | 0·5(0·3 to 0·6) |
| Peripheral artery disease | 132(86 to 197) | 570(395 to 797) | 5·2(5·0 to 5·4) | 0(0 to 0 ) | 0(0 to 0 ) | 1·3(1·0 to 1·5) |
| Endocarditis | 734(472 to 983) | 1,461(1,023 to 1,998) | 2·4(2·1 to 2·7) | 0(0 to 0 ) | 0(0 to 0 ) | -0·7(-1·0 to -0·4) |
| Chronic kidney disease due to hypertension | 28,727(23,475 to 34,466) | 70,260(56,866 to 84,481) | 3·1(3·0 to 3·3) | 4(4 to 5 ) | 4(3 to 5 ) | -0·2(-0·3 to 0·0) |
| Chronic kidney disease due to glomerulonephritis | 2,921(1,782 to 4,473) | 7,607(4,813 to 11,455) | 3·4(3·2 to 3·5) | 0(0 to 1 ) | 0(0 to 1 ) | 0·3(0·2 to 0·4) |
| Chronic kidney disease due to other and unspecified causes | 5,077(2,967 to 7,792) | 14,978(9,063 to 22,542) | 3·8(3·7 to 4·0) | 1(0 to 1 ) | 1(1 to 1 ) | 0·4(0·3 to 0·6) |
| Other cardiomyopathy | 1,327(764 to 2,484) | 3,786(2,520 to 5,284) | 3·7(3·4 to 3·9) | 0(0 to 0 ) | 0(0 to 0 ) | 0·7(0·5 to 0·9) |
| Non-rheumatic calcific aortic valve disease | 433(248 to 653) | 1,057(755 to 1,418) | 3·1(2·8 to 3·3) | 0(0 to 0 ) | 0(0 to 0 ) | 0·1(-0·2 to 0·3) |
| Chronic kidney disease due to diabetes mellitus type 1 | 2,696(1,520 to 4,208) | 4,991(2,819 to 7,840) | 2·2(1·9 to 2·4) | 0(0 to 0 ) | 0(0 to 0 ) | -0·4(-0·6 to -0·2) |
| Chronic kidney disease due to diabetes mellitus type 2 | 9,894(6,579 to 13,814) | 30,124(20,159 to 41,346) | 3·9(3·8 to 4·1) | 2(1 to 2 ) | 2(1 to 2 ) | 0·4(0·3 to 0·6) |
| Other cardiovascular and circulatory diseases | 4,290(3,241 to 6,106) | 7,875(6,154 to 9,818) | 2·1(1·8 to 2·3) | 1(0 to 1 ) | 0(0 to 1 ) | -1·2(-1·4 to -0·9) |
| **High body-mass index** |  |  |  |  |  |  |
| Esophageal cancer | 11,783(1,922 to 32,264) | 36,181(9,426 to 79,606) | 3·9(3·8 to 4·1) | 1(0 to 4) | 2(0 to 4) | 0·8(0·5 to 1·0) |
| Liver cancer due to hepatitis B | 7,347(1,368 to 19,193) | 13,091(4,110 to 28,002) | 2·0(1·6 to 2·4) | 1(0 to 2) | 1(0 to 1) | -0·7(-1·1 to -0·3) |
| Liver cancer due to hepatitis C | 1,552(257 to 4,191) | 3,002(890 to 6,450) | 2·3(1·9 to 2·7) | 0(0 to 1) | 0(0 to 0) | -0·9(-1·3 to -0·5) |
| Liver cancer due to alcohol use | 821(153 to 2,304) | 1,832(586 to 4,043) | 2·9(2·4 to 3·4) | 0(0 to 0) | 0(0 to 0) | -0·1(-0·6 to 0·4) |
| Breast cancer | 2,191(392 to 5,325) | 12,059(3,393 to 25,190) | 6·1(5·7 to 6·4) | 0(0 to 1) | 1(0 to 1) | 2·8(2·4 to 3·2) |
| Uterine cancer | 1,222(316 to 2,618) | 2,967(1,306 to 5,508) | 3·1(2·9 to 3·3) | 0(0 to 0) | 0(0 to 0) | 0·1(-0·1 to 0·3) |
| Colon and rectum cancer | 1,818(400 to 4,351) | 14,147(5,480 to 26,648) | 7·3(7·1 to 7·5) | 0(0 to 1) | 1(0 to 1) | 4·1(3·9 to 4·3) |
| Gallbladder and biliary tract cancer | 625(143 to 1,593) | 3,518(1,330 to 6,901) | 6·1(5·9 to 6·4) | 0(0 to 0) | 0(0 to 0) | 2·8(2·6 to 3·0) |
| Pancreatic cancer | 456(78 to 1,231) | 4,236(1,108 to 9,602) | 8·0(7·8 to 8·2) | 0(0 to 0) | 0(0 to 0) | 4·7(4·5 to 4·9) |
| Ovarian cancer | 71(-2 to 222) | 571(-13 to 1,496) | 7·4(7·3 to 7·6) | 0(0 to 0) | 0(0 to 0) | 4·3(4·2 to 4·5) |
| Kidney cancer | 276(65 to 627) | 2,589(1,082 to 4,699) | 8·0(7·8 to 8·2) | 0(0 to 0) | 0(0 to 0) | 4·8(4·6 to 5·0) |
| Thyroid cancer | 107(22 to 264) | 536(174 to 1,120) | 5·7(5·5 to 6·0) | 0(0 to 0) | 0(0 to 0) | 2·6(2·4 to 2·8) |
| Non-Hodgkin lymphoma | 226(45 to 599) | 1,593(528 to 3,315) | 7·0(6·6 to 7·3) | 0(0 to 0) | 0(0 to 0) | 4·0(3·8 to 4·2) |
| Multiple myeloma | 101(18 to 262) | 544(169 to 1,135) | 6·0(5·8 to 6·2) | 0(0 to 0) | 0(0 to 0) | 2·9(2·7 to 3·0) |
| Ischemic heart disease | 39,406(9,214 to 89,039) | 203,609(84,285 to 367,441) | 5·8(5·6 to 6·0) | 5(1 to 12) | 11(5 to 21) | 2·7(2·5 to 2·9) |
| Ischemic stroke | 20,443(4,897 to 44,480) | 87,594(37,019 to 154,794) | 5·1(4·9 to 5·4) | 3(1 to 6) | 4(2 to 8) | 1·9(1·7 to 2·2) |
| Intracerebral hemorrhage | 67,093(16,290 to 147,424) | 162,957(72,331 to 276,642) | 3·1(2·8 to 3·4) | 8(2 to 17) | 8(4 to 14) | 0·2(-0·1 to 0·5) |
| Subarachnoid hemorrhage | 19,128(4,463 to 42,922) | 16,205(7,015 to 28,640) | -0·6(-0·8 to -0·4) | 2(0 to 5) | 1(0 to 1) | -3·3(-3·5 to -3·1) |
| Hypertensive heart disease | 31,035(7,085 to 70,851) | 73,300(28,352 to 142,308) | 2·9(2·4 to 3·5) | 5(1 to 12) | 4(2 to 9) | -0·4(-0·9 to 0·0) |
| Atrial fibrillation and flutter | 976(209 to 2,389) | 5,876(2,152 to 11,857) | 6·4(6·1 to 6·6) | 0(0 to 1) | 0(0 to 1) | 2·1(1·9 to 2·3) |
| Asthma | 2,514(549 to 6,524) | 3,270(1,235 to 6,227) | 0·9(0·5 to 1·3) | 0(0 to 1) | 0(0 to 0) | -2·4(-2·7 to -2·2) |
| Gallbladder and biliary diseases | 1,846(428 to 4,213) | 3,053(1,235 to 5,627) | 1·7(1·3 to 2·1) | 0(0 to 1) | 0(0 to 0) | -1·4(-1·8 to -1·1) |
| Alzheimer's disease and other dementias | 3,531(290 to 13,782) | 23,787(3,264 to 78,659) | 6·8(6·7 to 6·9) | 1(0 to 3) | 2(0 to 6) | 2·3(2·2 to 2·5) |
| Chronic kidney disease due to hypertension | 3,019(645 to 7,702) | 14,049(4,767 to 27,581) | 5·4(5·2 to 5·6) | 0(0 to 1) | 1(0 to 2) | 2·1(1·9 to 2·4) |
| Chronic kidney disease due to glomerulonephritis | 951(179 to 2,440) | 3,601(1,176 to 7,307) | 4·7(4·5 to 4·9) | 0(0 to 0) | 0(0 to 0) | 1·7(1·5 to 2·0) |
| Chronic kidney disease due to other and unspecified causes | 1,501(263 to 3,972) | 6,306(2,066 to 12,869) | 5·0(4·8 to 5·3) | 0(0 to 0) | 0(0 to 1) | 1·9(1·7 to 2·2) |
| Acute lymphoid leukemia | 74(13 to 206) | 430(150 to 859) | 6·3(6·1 to 6·5) | 0(0 to 0) | 0(0 to 0) | 4·0(3·8 to 4·1) |
| Chronic lymphoid leukemia | 28(5 to 73) | 216(77 to 420) | 7·3(7·0 to 7·6) | 0(0 to 0) | 0(0 to 0) | 4·5(4·1 to 4·9) |
| Acute myeloid leukemia | 55(10 to 142) | 331(120 to 648) | 6·4(6·2 to 6·5) | 0(0 to 0) | 0(0 to 0) | 3·8(3·7 to 4·0) |
| Chronic myeloid leukemia | 24(5 to 61) | 53(19 to 103) | 2·8(2·5 to 3·1) | 0(0 to 0) | 0(0 to 0) | 0·2(-0·1 to 0·5) |
| Other leukemia | 650(124 to 1,651) | 1,505(537 to 3,002) | 2·9(2·7 to 3·2) | 0(0 to 0) | 0(0 to 0) | 0·5(0·3 to 0·7) |
| Diabetes mellitus type 2 | 10,510(2,938 to 21,514) | 47,530(22,514 to 76,632) | 5·3(5·2 to 5·5) | 1(0 to 3) | 2(1 to 4) | 2·3(2·1 to 2·5) |
| Chronic kidney disease due to diabetes mellitus type 2 | 2,979(655 to 7,076) | 13,122(4,496 to 24,541) | 5·2(5·0 to 5·5) | 0(0 to 1) | 1(0 to 1) | 2·0(1·7 to 2·3) |
| Liver cancer due to other causes | 642(115 to 1,706) | 1,040(325 to 2,200) | 1·7(1·3 to 2·1) | 0(0 to 0) | 0(0 to 0) | -1·0(-1·3 to -0·6) |
| Total burden related to hepatitis B | 7,347(1,368 to 19,193) | 13,091(4,110 to 28,002) | 2·0(1·6 to 2·4) | 1(0 to 2) | 1(0 to 1) | -0·7(-1·1 to -0·3) |
| Total burden related to hepatitis C | 1,552(257 to 4,191) | 3,002(890 to 6,450) | 2·3(1·9 to 2·7) | 0(0 to 1) | 0(0 to 0) | -0·9(-1·3 to -0·5) |
| Total cancers | 30,067(6,567 to 73,656) | 100,442(41,168 to 185,335) | 4·3(4·2 to 4·4) | 4(1 to 9) | 5(2 to 9) | 1·2(0·9 to 1·5) |
| **Low bone mineral density** |  |  |  |  |  |  |
| Pedestrian road injuries | 8,676(6,571 to 12,625) | 16,282(12,850 to 19,527) | 2·2(2·0 to 2·4) | 1(1 to 2) | 1(1 to 1) | -0·7(-0·9 to -0·5) |
| Cyclist road injuries | 882(522 to 1,343) | 2,439(1,888 to 3,057) | 3·6(3·5 to 3·7) | 0(0 to 0) | 0(0 to 0) | 0·7(0·5 to 0·9) |
| Motorcyclist road injuries | 1,767(1,083 to 2,802) | 4,128(2,891 to 5,402) | 3·0(2·7 to 3·2) | 0(0 to 0) | 0(0 to 0) | 0·3(0·0 to 0·5) |
| Motor vehicle road injuries | 1,912(1,222 to 3,410) | 4,457(3,280 to 5,731) | 2·9(2·6 to 3·3) | 0(0 to 0) | 0(0 to 0) | 0·2(0·0 to 0·4) |
| Other road injuries | 79(52 to 115) | 218(170 to 270) | 3·5(3·2 to 3·8) | 0(0 to 0) | 0(0 to 0) | 0·5(0·3 to 0·8) |
| Other transport injuries | 1,934(1,550 to 2,649) | 1,709(1,356 to 2,073) | -0·5(-0·9 to 0·0) | 0(0 to 0) | 0(0 to 0) | -3·3(-3·8 to -2·9) |
| Falls | 14,512(11,965 to 18,810) | 56,639(30,514 to 71,875) | 4·8(4·4 to 5·2) | 3(3 to 4) | 4(2 to 5) | 0·9(0·6 to 1·1) |
| Other exposure to mechanical forces | 1,446(1,043 to 2,338) | 3,436(2,092 to 4,376) | 3·1(2·7 to 3·5) | 0(0 to 0) | 0(0 to 0) | 0·1(-0·4 to 0·6) |
| Non-venomous animal contact | 344(221 to 412) | 184(145 to 238) | -2·1(-2·4 to -1·9) | 0(0 to 0) | 0(0 to 0) | -5·0(-5·2 to -4·8) |
| Physical violence by other means | 449(367 to 527) | 366(296 to 447) | -0·7(-0·9 to -0·5) | 0(0 to 0) | 0(0 to 0) | -3·7(-3·9 to -3·6) |
| **Kidney dysfunction** |  |  |  |  |  |  |
| Ischemic heart disease | 61,221(39,573 to 85,649) | 218,411(137,830 to 306,684) | 4·5(4·0 to 5·0) | 10(6 to 15) | 14(9 to 20) | 1·0(0·6 to 1·5) |
| Ischemic stroke | 30,537(22,140 to 40,871) | 77,268(51,264 to 104,740) | 3·3(3·0 to 3·6) | 4(3 to 6) | 4(3 to 6) | -0·1(-0·4 to 0·2) |
| Intracerebral hemorrhage | 57,893(44,649 to 74,893) | 80,860(59,370 to 103,804) | 1·2(0·9 to 1·4) | 8(6 to 11) | 4(3 to 6) | -2·0(-2·3 to -1·7) |
| Peripheral artery disease | 86(60 to 125) | 272(188 to 374) | 4·2(3·9 to 4·4) | 0(0 to 0) | 0(0 to 0) | 0·9(0·7 to 1·1) |
| Chronic kidney disease due to hypertension | 28,727(23,475 to 34,466) | 70,260(56,866 to 84,481) | 3·1(3·0 to 3·3) | 4(4 to 5) | 4(3 to 5) | -0·2(-0·3 to 0·0) |
| Chronic kidney disease due to glomerulonephritis | 11,485(8,771 to 14,968) | 17,390(12,363 to 23,823) | 1·4(1·2 to 1·7) | 1(1 to 2) | 1(1 to 1) | -1·0(-1·2 to -0·8) |
| Chronic kidney disease due to other and unspecified causes | 19,800(15,463 to 25,628) | 33,043(23,283 to 45,006) | 1·8(1·6 to 1·9) | 2(2 to 3) | 2(1 to 3) | -0·8(-1·0 to -0·6) |
| Chronic kidney disease due to diabetes mellitus type 1 | 11,183(7,847 to 14,946) | 12,679(8,458 to 18,334) | 0·4(0·2 to 0·7) | 1(1 to 1) | 1(0 to 1) | -1·7(-2·0 to -1·4) |
| Chronic kidney disease due to diabetes mellitus type 2 | 27,413(21,889 to 33,263) | 63,354(49,787 to 77,280) | 2·9(2·8 to 3·1) | 4(3 to 5) | 4(3 to 4) | -0·3(-0·4 to -0·1) |
| **High LDL cholesterol** |  |  |  |  |  |  |
| Ischemic heart disease | 249,254(194,105 to 314,056) | 733,295(520,883 to 982,246) | 3·8(3·5 to 4·0) | 38(27 to 51) | 45(30 to 62) | 0·6(0·4 to 0·8) |
| Ischemic stroke | 67,806(33,489 to 130,613) | 182,689(71,552 to 383,129) | 3·5(3·2 to 3·7) | 11(4 to 23) | 11(4 to 24) | 0·0(-0·3 to 0·3) |

AAPC, average annual percent changes; DALYs, disability-adjusted life years; UI, uncertainty interval; CI, confidence interval.

**sTable 5**. Population attributable fractions (PAFs) in DALYs, YLDs, YLLs and deaths of level 2 causes attributable to six metabolic risk factors in China, 2019

|  | **Age standardized attributable burden (per 100,000)** | | | |
| --- | --- | --- | --- | --- |
|  | YLDs (%, 95%CI) | DALYs (%, 95%CI) | YLL (%, 95%CI) | Death (%, 95%CI) |
| **High body-mass index** |  |  |  |  |
| Cardiovascular diseases | 15·3(7·4 to 24·3) | 15·1(7·3 to 24·6) | 15·1(7·2 to 24·5) | 10·5(4·7 to 18·2) |
| Chronic respiratory diseases | 2·0(0·9 to 3·4) | 0·8(0·4 to 1·4) | 0·4(0·1 to 0·7) | 0·3(0·1 to 0·5) |
| Diabetes and kidney diseases | 35·7(19·7 to 51·9) | 29·4(15·8 to 43·5) | 23·1(11·8 to 36·2) | 20·5(9·9 to 33·4) |
| Digestive diseases | 9·1(4·1 to 16·0) | 3·6(1·5 to 6·5) | 0·8(0·3 to 1·5) | 1·3(0·5 to 2·3) |
| Musculoskeletal disorders | 3·5(1·5 to 6·4) | 3·5(1·4 to 6·3) | - | - |
| Neoplasms | 4·1(1·8 to 7·2) | 3·7(1·6 to 6·7) | 3·7(1·6 to 6·7) | 3·5(1·5 to 6·4) |
| Neurological disorders | 1·4(0·3 to 3·8) | 2·6(0·6 to 6·8) | 4·7(1·1 to 11·4) | 5·3(1·4 to 12·5) |
| Sense organ diseases | 0·3(0·1 to 0·6) | 0·3(0·1 to 0·6) | - | - |
| **High fasting plasma glucose** |  |  |  |  |
| Cardiovascular diseases | 12·3(8·6 to 18·6) | 14·5(10·6 to 20·2) | 14·7(10·8 to 20·5) | 14·9(10·3 to 21·9) |
| Diabetes and kidney diseases | 83·5(79·1 to 87·5) | 73·8(70·4 to 77·5) | 64·4(61·3 to 67·3) | 64·8(61·7 to 67·7) |
| Neoplasms | 2·6(0·7 to 5·4) | 2·8(0·7 to 5·9) | 2·8(0·7 to 5·9) | 3·3(0·9 to 7·0) |
| Neurological disorders | 1·4(0·2 to 4·0) | 2·5(0·4 to 7·0) | 4·5(0·8 to 11·4) | 5·4(1·1 to 13·2) |
| Respiratory infections and tuberculosis | 1·4(0·7 to 2·3) | 1·2(0·7 to 1·7) | 1·2(0·7 to 1·7) | 1·2(0·7 to 1·8) |
| Sense organ diseases | 0·5(0·1 to 1·2) | 0·5(0·1 to 1·2) | - | - |
| **High LDL cholesterol** |  |  |  |  |
| Cardiovascular diseases | 18·5(13·5 to 25·4) | 21·3(16·7 to 26·9) | 21·7(17·1 to 27·0) | 20·2(14·3 to 27·4) |
| **High systolic blood pressure** |  |  |  |  |
| Cardiovascular diseases | 51·3(44·0 to 57·7) | 54·1(47·6 to 60·5) | 54·4(47·8 to 60·9) | 52·7(45·1 to 60·7) |
| Diabetes and kidney diseases | 11·0(8·0 to 14·7) | 21·4(17·8 to 24·9) | 31·6(27·9 to 34·8) | 34·8(31·1 to 37·8) |
| **Kidney dysfunction** |  |  |  |  |
| Cardiovascular diseases | 6·9(5·6 to 8·2) | 8·0(6·4 to 9·7) | 8·1(6·4 to 9·9) | 8·2(6·2 to 10·2) |
| Diabetes and kidney diseases | 21·9(16·3 to 27·7) | 38·3(33·2 to 42·9) | 54·3(53·2 to 55·5) | 53·3(52·1 to 54·6) |
| Musculoskeletal disorders | 0·2(0·1 to 0·2) | 0·2(0·1 to 0·2) | - | - |
| **Low bone mineral density** |  |  |  |  |
| Self-harm and interpersonal violence | 1·4(1·0 to 2·0) | 0·4(0·3 to 0·5) | 0·2(0·1 to 0·2) | 0·2(0·2 to 0·3) |
| Transport injuries | 13·9(12·0 to 14·9) | 7·2(6·1 to 7·9) | 5·7(4·8 to 6·2) | 9·8(8·4 to 10·6) |
| Unintentional injuries | 20·3(17·7 to 22·4) | 10·3(8·6 to 11·7) | 6·5(4·4 to 7·8) | 19·9(14·0 to 23·4) |

DALYs, disability-adjusted life years; YLDs, years lived with disability; YLLs, years of life lost; CI, confidence interval.

**sTable 6.** Correlation between SDI and DALYs, YLLs, YLDs and deaths from 1990 to 2019

|  | **Number** | | | | **Age standardized rate (per 100,000)** | | | |
| --- | --- | --- | --- | --- | --- | --- | --- | --- |
|  | DALYs | YLDs | Deaths | YLLs | DALYs | YLDs | Deaths | YLLs |
| High fasting plasma glucose | 0·945* | 0·991* | 0·931* | 0·899* | -0·099 | 0·430* | 0·016 | -0·343* |
| High systolic blood pressure | 0·995* | 1·000* | 0·995* | 0·995* | -0·789* | 0·982* | -0·591* | -0·811* |
| High body-mass index | 1·000* | 1·000* | 1·000* | 1·000* | 0·982* | 1·000* | 0·968* | 0·945* |
| Low bone mineral density | 1·000* | 0·890* | 1·000* | 0·811* | 0·411* | 0·099 | 0·232 | 0·090 |
| Kidney dysfunction | 0·972* | 0·982* | 0·991* | 0·972* | -0·255* | 0·678* | 0·269* | -0·503* |
| High LDL cholesterol | 1·000* | 1·000* | 1·000* | 1·000* | 0·402* | 1·000* | 0·508* | 0·366* |

DALYs, disability-adjusted life years; YLDs, years lived with disability; YLLs, years of life lost, *denotes P<0·05.

**sTable 7**. The value and ranking in the number and age standardized rate of DALYs and deaths attributable to six metabolic risk factors in 19 countries of Group 20 except European Union

| Country | Number | | | | Age standardized rate (per 100,000) | | | |
| --- | --- | --- | --- | --- | --- | --- | --- | --- |
|  | Death | rank | DALYs | rank | Death | rank | DALYs | rank |
| **High fasting plasma glucose** | | | | | | | | |
| China | 1,067,554 | 2 | 28,228,439 | 2 | 62 | 12 | 3,270 | 4 |
| Indonesia | 259,251 | 4 | 7,541,726 | 4 | 142 | 3 | 2,871 | 5 |
| Russian Federation | 196,912 | 5 | 4,311,463 | 7 | 84 | 7 | 2,074 | 6 |
| Japan | 101,141 | 9 | 2,322,033 | 9 | 21 | 19 | 3,765 | 2 |
| Republic of Korea | 39,832 | 15 | 1,035,582 | 14 | 47 | 15 | 2,024 | 7 |
| Australia | 17,650 | 19 | 381,856 | 19 | 37 | 17 | 3,888 | 1 |
| France | 40,295 | 14 | 797,290 | 16 | 23 | 18 | 2,003 | 8 |
| Germany | 150,713 | 8 | 2,937,451 | 8 | 67 | 11 | 1,018 | 16 |
| Italy | 95,753 | 10 | 1,848,809 | 10 | 53 | 14 | 1,764 | 10 |
| United Kingdom | 75,454 | 11 | 1,739,312 | 12 | 53 | 13 | 1,473 | 12 |
| Argentina | 39,517 | 16 | 945,775 | 15 | 71 | 10 | 1,292 | 14 |
| Canada | 30,727 | 17 | 686,456 | 18 | 41 | 16 | 1,559 | 11 |
| United States of America | 439,379 | 3 | 10,827,167 | 3 | 73 | 9 | 600 | 19 |
| Mexico | 157,908 | 7 | 4,617,529 | 6 | 143 | 2 | 920 | 17 |
| Brazil | 174,198 | 6 | 4,778,226 | 5 | 77 | 8 | 1,185 | 15 |
| Saudi Arabia | 19,543 | 18 | 743,503 | 17 | 148 | 1 | 722 | 18 |
| Turkey | 72,369 | 12 | 1,797,315 | 11 | 89 | 6 | 1,879 | 9 |
| India | 1,121,914 | 1 | 33,097,681 | 1 | 111 | 5 | 3,406 | 3 |
| South Africa | 54,459 | 13 | 1,446,008 | 13 | 140 | 4 | 1,452 | 13 |
| **High systolic blood pressure** | | | | | | | | |
| China | 2,599,879 | 1 | 54,441,615 | 1 | 153 | 5 | 2,844 | 6 |
| Indonesia | 477,723 | 5 | 12,244,423 | 3 | 274 | 1 | 5,690 | 1 |
| Russian Federation | 557,781 | 3 | 10,916,126 | 4 | 239 | 2 | 4,770 | 2 |
| Japan | 196,385 | 8 | 3,122,870 | 8 | 42 | 19 | 924 | 17 |
| Republic of Korea | 37,142 | 17 | 688,992 | 18 | 45 | 18 | 796 | 19 |
| Australia | 25,498 | 19 | 406,445 | 19 | 54 | 16 | 966 | 16 |
| France | 89,170 | 12 | 1,274,057 | 14 | 49 | 17 | 903 | 18 |
| Germany | 224,494 | 7 | 3,129,401 | 7 | 98 | 11 | 1,563 | 12 |
| Italy | 123,001 | 10 | 1,661,664 | 11 | 66 | 13 | 1,082 | 14 |
| United Kingdom | 87,762 | 13 | 1,441,912 | 12 | 63 | 14 | 1,177 | 13 |
| Argentina | 59,080 | 15 | 1,092,243 | 15 | 107 | 9 | 2,037 | 10 |
| Canada | 41,799 | 16 | 696,317 | 17 | 54 | 15 | 1,013 | 15 |
| United States of America | 495,201 | 4 | 9,102,668 | 5 | 82 | 12 | 1,684 | 11 |
| Mexico | 129,019 | 9 | 2,876,435 | 9 | 118 | 8 | 2,456 | 8 |
| Brazil | 239,416 | 6 | 5,436,513 | 6 | 105 | 10 | 2,283 | 9 |
| Saudi Arabia | 28,995 | 18 | 892,166 | 16 | 210 | 3 | 4,431 | 3 |
| Turkey | 109,162 | 11 | 2,165,550 | 10 | 134 | 7 | 2,503 | 7 |
| India | 1,471,889 | 2 | 37,059,855 | 2 | 144 | 6 | 3,194 | 5 |
| South Africa | 61,473 | 14 | 1,424,097 | 13 | 162 | 4 | 3,209 | 4 |
| **High body-mass index** | | | | | | | | |
| China | 764,698 | 1 | 24,830,041 | 1 | 41 | 15 | 1,231 | 15 |
| Indonesia | 186,657 | 5 | 7,008,297 | 5 | 80 | 6 | 2,700 | 5 |
| Russian Federation | 283,793 | 4 | 7,346,876 | 4 | 123 | 3 | 3,263 | 4 |
| Japan | 51,822 | 13 | 1,361,819 | 13 | 13 | 19 | 503 | 19 |
| Republic of Korea | 19,333 | 18 | 645,697 | 18 | 23 | 18 | 752 | 18 |
| Australia | 18,713 | 19 | 525,135 | 19 | 42 | 13 | 1,388 | 13 |
| France | 46,898 | 14 | 1,096,913 | 15 | 29 | 17 | 920 | 17 |
| Germany | 106,143 | 8 | 2,493,051 | 8 | 50 | 11 | 1,479 | 12 |
| Italy | 64,136 | 10 | 1,455,854 | 12 | 38 | 16 | 1,134 | 16 |
| United Kingdom | 56,216 | 11 | 1,721,261 | 10 | 42 | 12 | 1,601 | 11 |
| Argentina | 36,676 | 15 | 1,022,339 | 16 | 67 | 9 | 1,953 | 9 |
| Canada | 29,814 | 16 | 833,879 | 17 | 41 | 14 | 1,332 | 14 |
| United States of America | 393,859 | 3 | 12,536,102 | 3 | 69 | 8 | 2,498 | 7 |
| Mexico | 125,591 | 7 | 4,340,212 | 7 | 110 | 4 | 3,555 | 3 |
| Brazil | 177,940 | 6 | 5,817,939 | 6 | 76 | 7 | 2,405 | 8 |
| Saudi Arabia | 28,039 | 17 | 1,183,820 | 14 | 161 | 1 | 4,772 | 1 |
| Turkey | 80,118 | 9 | 2,381,067 | 9 | 95 | 5 | 2,663 | 6 |
| India | 579,108 | 2 | 21,017,844 | 2 | 52 | 10 | 1,698 | 10 |
| South Africa | 55,365 | 12 | 1,685,698 | 11 | 135 | 2 | 3,609 | 2 |
| **Low bone mineral density** | | | | | | | | |
| China | 89,857 | 2 | 3,320,275 | 2 | 6 | 4 | 177 | 10 |
| Indonesia | 10,053 | 5 | 349,904 | 7 | 7 | 3 | 175 | 11 |
| Russian Federation | 6,163 | 10 | 508,498 | 4 | 3 | 18 | 227 | 4 |
| Japan | 8,921 | 8 | 445,612 | 5 | 2 | 19 | 126 | 19 |
| Republic of Korea | 3,658 | 14 | 166,831 | 13 | 4 | 10 | 191 | 8 |
| Australia | 2,184 | 17 | 98,550 | 17 | 4 | 11 | 232 | 3 |
| France | 9,997 | 6 | 320,699 | 9 | 5 | 6 | 214 | 5 |
| Germany | 9,281 | 7 | 345,962 | 8 | 4 | 14 | 173 | 12 |
| Italy | 7,590 | 9 | 238,109 | 10 | 4 | 15 | 154 | 16 |
| United Kingdom | 4,962 | 12 | 199,963 | 11 | 3 | 16 | 156 | 14 |
| Argentina | 1,572 | 19 | 76,354 | 18 | 3 | 17 | 144 | 17 |
| Canada | 3,967 | 13 | 137,821 | 14 | 5 | 7 | 193 | 7 |
| United States of America | 27,681 | 3 | 1,172,666 | 3 | 4 | 9 | 210 | 6 |
| Mexico | 5,002 | 11 | 198,854 | 12 | 5 | 8 | 169 | 13 |
| Brazil | 12,135 | 4 | 438,928 | 6 | 5 | 5 | 186 | 9 |
| Saudi Arabia | 2,335 | 16 | 108,870 | 16 | 12 | 2 | 434 | 1 |
| Turkey | 3,178 | 15 | 109,110 | 15 | 4 | 13 | 126 | 18 |
| India | 118,682 | 1 | 3,988,261 | 1 | 14 | 1 | 382 | 2 |
| South Africa | 1,821 | 18 | 73,712 | 19 | 4 | 12 | 155 | 15 |
| **Kidney dysfunction** | | | | | | | | |
| China | 573,537 | 1 | 13,353,909 | 2 | 34 | 10 | 709 | 11 |
| Indonesia | 106,271 | 5 | 3,328,918 | 4 | 56 | 3 | 1,438 | 3 |
| Russian Federation | 111,327 | 4 | 2,330,434 | 6 | 48 | 6 | 1,037 | 7 |
| Japan | 78,418 | 7 | 1,245,500 | 8 | 16 | 17 | 348 | 15 |
| Republic of Korea | 14,303 | 17 | 276,537 | 17 | 17 | 16 | 325 | 16 |
| Australia | 10,346 | 19 | 153,080 | 19 | 21 | 13 | 355 | 14 |
| France | 24,539 | 12 | 339,163 | 16 | 13 | 19 | 230 | 19 |
| Germany | 67,422 | 9 | 896,339 | 10 | 28 | 12 | 430 | 12 |
| Italy | 34,569 | 11 | 462,429 | 12 | 18 | 15 | 297 | 17 |
| United Kingdom | 21,965 | 14 | 351,216 | 15 | 15 | 18 | 280 | 18 |
| Argentina | 23,809 | 13 | 451,598 | 13 | 43 | 8 | 846 | 8 |
| Canada | 15,582 | 16 | 249,850 | 18 | 20 | 14 | 366 | 13 |
| United States of America | 214,741 | 3 | 3,934,538 | 3 | 35 | 9 | 725 | 10 |
| Mexico | 100,009 | 6 | 2,677,877 | 5 | 89 | 2 | 2,231 | 2 |
| Brazil | 73,294 | 8 | 1,828,988 | 7 | 32 | 11 | 778 | 9 |
| Saudi Arabia | 14,298 | 18 | 450,378 | 14 | 113 | 1 | 2,370 | 1 |
| Turkey | 43,971 | 10 | 912,927 | 9 | 54 | 4 | 1,075 | 6 |
| India | 469,419 | 2 | 13,411,730 | 1 | 45 | 7 | 1,126 | 4 |
| South Africa | 18,830 | 15 | 508,798 | 11 | 48 | 5 | 1,094 | 5 |
| **High LDL cholesterol** | | | | | | | | |
| China | 915,983 | 1 | 19,813,962 | 1 | 56 | 5 | 1,052 | 6 |
| Indonesia | 112,173 | 5 | 3,254,386 | 5 | 60 | 3 | 1,379 | 4 |
| Russian Federation | 332,217 | 3 | 6,388,323 | 3 | 142 | 1 | 2,806 | 1 |
| Japan | 75,782 | 8 | 1,098,335 | 9 | 16 | 19 | 348 | 18 |
| Republic of Korea | 15,522 | 17 | 286,052 | 18 | 19 | 18 | 337 | 19 |
| Australia | 14,013 | 19 | 212,393 | 19 | 30 | 14 | 525 | 15 |
| France | 37,831 | 13 | 535,426 | 14 | 22 | 17 | 403 | 17 |
| Germany | 94,572 | 7 | 1,365,147 | 7 | 43 | 9 | 726 | 12 |
| Italy | 53,140 | 9 | 712,073 | 12 | 29 | 16 | 495 | 16 |
| United Kingdom | 48,028 | 11 | 777,929 | 11 | 35 | 13 | 663 | 13 |
| Argentina | 22,649 | 14 | 430,443 | 15 | 41 | 10 | 818 | 9 |
| Canada | 22,409 | 15 | 373,965 | 16 | 30 | 15 | 573 | 14 |
| United States of America | 226,343 | 4 | 4,167,272 | 4 | 38 | 12 | 805 | 10 |
| Mexico | 52,139 | 10 | 1,140,364 | 8 | 48 | 7 | 961 | 8 |
| Brazil | 99,375 | 6 | 2,363,141 | 6 | 43 | 8 | 981 | 7 |
| Saudi Arabia | 17,926 | 16 | 609,556 | 13 | 103 | 2 | 2,406 | 2 |
| Turkey | 44,344 | 12 | 942,752 | 10 | 53 | 6 | 1,061 | 5 |
| India | 630,093 | 2 | 17,679,116 | 2 | 60 | 4 | 1,461 | 3 |
| South Africa | 14,727 | 18 | 358,968 | 17 | 38 | 11 | 786 | 11 |

DALYs, disability-adjusted life years.
